# Evolutionary structure and timing of major habitat shifts in Crocodylomorpha

**DOI:** 10.1038/s41598-018-36795-1

**Published:** 2019-01-24

**Authors:** Eric W. Wilberg, Alan H. Turner, Christopher A. Brochu

**Affiliations:** 1 Department of Anatomical Sciences, Stony Brook University, Stony Brook, NY 11794 USA; 2 Department of Earth and Environmental Sciences, University of Iowa, Iowa City, IA 52242 USA

## Abstract

Extant crocodylomorphs are semiaquatic ambush predators largely restricted to freshwater or estuarine environments, but the group is ancestrally terrestrial and inhabited a variety of ecosystems in the past. Despite its rich ecological history, little effort has focused on elucidating the historical pattern of ecological transitions in the group. Traditional views suggested a single shift from terrestrial to aquatic in the Early Jurassic. However, new fossil discoveries and phylogenetic analyses tend to imply a multiple-shift model. Here we estimate ancestral habitats across a comprehensive phylogeny and show at least three independent shifts from terrestrial to aquatic and numerous other habitat transitions. Neosuchians first invade freshwater habitats in the Jurassic, with up to four subsequent shifts into the marine realm. Thalattosuchians first appear in marine habitats in the Early Jurassic. Freshwater semiaquatic mahajangasuchids are derived from otherwise terrestrial notosuchians. Within nearly all marine groups, some species return to freshwater environments. Only twice have crocodylomorphs reverted from aquatic to terrestrial habitats, both within the crown group. All living non-alligatorid crocodylians have a keratinised tongue with salt-excreting glands, but the lack of osteological correlates for these adaptations complicates pinpointing their evolutionary origin or loss. Based on the pattern of transitions to the marine realm, our analysis suggests at least four independent origins of saltwater tolerance in Crocodylomorpha.

## Introduction

Living crocodylomorphs (crown-group Crocodylia) are semiaquatic ambush predators largely restricted to freshwater or estuarine environments. However, the group is ancestrally terrestrial^1^ and has filled a wide variety of ecological niches in the past, in some ways comparable to Cenozoic mammals. Extinct crocodylomorphs include large fully marine taxa analogous to modern toothed whales (e.g., refs^2,3^), small terrestrial herbivores (e.g., refs^4–6^), and large terrestrial apex predators (e.g., refs^7–9^). Despite this ecological diversity, little is known about the timing or pattern of major habitat shifts within Crocodylomorpha.

**Figure 1:**
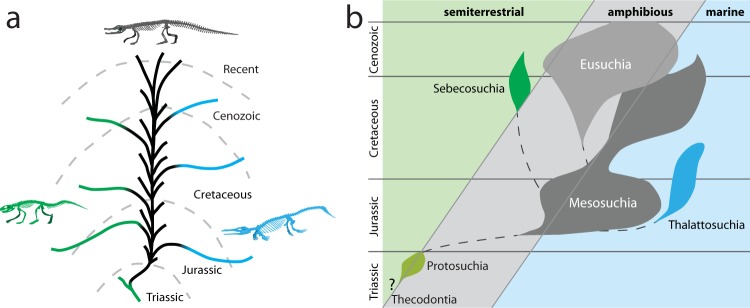
Traditional hypotheses of crocodylomorph habitat transition underpinning the single-shift model. (**a**) The “fertile Stamm” – a freshwater, semiaquatic phylogenetic core from which multiple lineages independently shift to terrestrial or marine ecosystems (adapted from ref.^11^). (**b**) Spindle diagram of Langston^12^, depicting a single shift to the aquatic realm from “Protosuchia” to “Mesosuchia” in the Early Jurassic (adapted from ref.^12^). Green represents terrestrial habitats, gray/black represents freshwater semiaquatic, blue represents marine.

Traditional views on crocodylomorph evolution posit a terrestrial origin, followed by a shift to a freshwater semiaquatic habitat early in the history of the group (e.g., refs^10–13^). From this freshwater semiaquatic phylogenetic core – what von Huene^11^ called the “fertile Stamm” – numerous crocodylomorph lineages moved into marine realms or shifted back onto land (Fig. 1). This traditional view established a single-shift model, with the transition from a terrestrial to an aquatic mode of life occurring early in the group’s history. For the purpose of this study our use of the term “aquatic” refers to taxa inhabiting either freshwater and marine habitats.

Decades of fossil discoveries and modern phylogenetic hypotheses call the single-shift model into question. While some disagreement among phylogenetic hypotheses remains, nearly all imply more than one transition from terrestrial to aquatic. The highly marine-adapted clade Thalattosuchia may either be deeply nested among other marine neosuchians or represent a group of very early diverging crocodylomorphs^14–18^. If thalattosuchians diverged early, this suggests at least two separate shifts from terrestrial to aquatic ecologies. Additionally, a number of Cretaceous Gondwanan crocodyliforms (e.g., *Mahajangasuchus*, *Kaprosuchus*, *Stolokrosuchus*) exhibit cranial adaptations typically found in definitively semiaquatic taxa such as elongate platyrostral or tube-like snouts, orbits located dorsally on the skull, and/or dorsally-facing external nares^19–21^. While the exact placement of these taxa can vary among competing phylogenetic hypotheses, they are generally recovered as part of the largely terrestrial notosuchian radiation^22–26^. These advances collectively suggest that a multiple-shift model better explains the pattern and timing of ecological shifts within Crocodylomorpha.

In addition to shifts from terrestrial to aquatic, some crocodylomorphs nested within aquatic clades appear to have reverted to non-aquatic habitats, and shifts from freshwater to marine environments potentially occurred numerous times. The ability to utilize marine environments is predicated on adaptations for saltwater tolerance. All living non-alligatorid crocodylians have a keratinised tongue with salt-excreting glands^27–29^, facilitating the use of marine habitats (even if some extant species are entirely restricted to freshwater environments today). Unfortunately, these adaptations in extant taxa do not leave osteological correlates, confounding attempts to pinpoint their origin in the fossil record. However, the persistent presence of certain fossil crocodylomorph taxa in marine sediments implies some type of adaptation for salt tolerance. Estimating where in crocodylomorph phylogeny these marine transitions occurred will help constrain when adaptations related to saltwater tolerance evolved.

Modern, large-scale analyses of crocodylomorph phylogeny are becoming increasingly common (e.g., refs^18,23,30–33^). By combining comprehensive phylogenetic hypotheses with habitat data, we reconstruct the evolutionary sequence of habitat shifts within Crocodylomorpha. Understanding this sequence of transitions is an important first step towards understanding the related morphological adaptations that accompany transitions to novel environments.

## Methods

### Tree Building and Time Calibration

We performed a new phylogenetic analysis based on the dataset of Wilberg^34^. Taxon sampling was increased to 100 taxa (8 outgroup and 92 ingroup) and character sampling increased to 407 (the phylogenetic data matrix, character descriptions, and materials referenced for character scoring are available in the Supplementary Information). The dataset was analyzed with TNT v1.5^35,36^ using equally weighted parsimony. A heuristic search included 1000 replicates of Wagner trees using random addition sequences followed by TBR (tree bisection and reconnection) branch swapping holding 10 trees per replication. The shortest trees obtained from these replicates were subjected to a final round of TBR branch swapping to ensure all minimum length trees were discovered. Zero-length branches were collapsed if they lacked support under any of the minimal length trees (Rule 1 of Coddington and Scharff^37^).

To increase our coverage of Eusuchia for the ancestral habitat reconstruction analysis, we constructed an informal supertree by grafting of the topology of Eusuchia (based on refs^31,38^) onto the appropriate portion of the tree, resulting in a tree with 144 terminal taxa (including 49 crown-group taxa). To estimate branch lengths, we first compiled geological ages for all ingroup taxa. Ages for fossil occurrences were either set at stratigraphic midpoint values (typically at Stage level) or taken from absolute values of radioisotopic dates if available. These were obtained from the literature and/or via the Paleobiology Database (www.paleodb.org; stratigraphic data utilized in this study are available in Supplementary Information). Boundary ages follow Gradstein *et al*.^39^. The phylogeny was time-calibrated and branch lengths were generated using the geological age of the terminal taxa and temporally smoothed ghost lineage analysis, as outlined in Ruta *et al*.^40^ and subsequently used by various authors^41–44^. Time-calibrated trees were generated using the R^45^ software library ‘paleotree’^46^ with the “equal” option in the function TimePaleoPhy. Under the “equal” option, a value (vartime) must be provided that controls for the amount of time added to the root that will be used to smooth time along the unconstrained internodes. We chose a value corresponding to 5 million years. Choice of “vartime” value had little to no effect on reconstructed node ages. Our time-calibrated tree file is available in the Supplementary Information.

### Ancestral Habitat Reconstruction

Each crocodylomorph species was assigned one of three broad habitat categories: terrestrial, freshwater semiaquatic, or marine. Most extant crocodylians are restricted to freshwater habitats. While some species of *Crocodylus* frequent marine environments (e.g. *C*. *porosus*), they inhabit predominantly freshwater and brackish environments. Thus, we chose to classify all extant crocodylians as freshwater. Habitat assignments of fossil crocodylomorphs were based on a combination of the depositional environment in which the fossils were found and their morphology. Taxa from terrestrial deposits (e.g. fluvial; lacustrine) were assigned as freshwater if they showed gross morphological similarity to extant semiaquatic crocodylians, or terrestrial if they possessed morphological features posited to correlate with terrestriality (e.g., dorsoventrally tall cranium, elongate limbs, well-developed 4^th^ trochanter of the femur; refs^9,22,47–50^). Taxa found exclusively in marine sediments were assigned to the marine habitat category. Within the marine category, some taxa show adaptations for a fully pelagic lifestyle (e.g. hypocercal tail fin, paddle-like limbs; refs^2,3,18^). However, for this study we have chosen to group these taxa with semiaquatic coastal marine species. Ancestral states were reconstructed in Mesquite v3.10^51^ using maximum likelihood (ML) under a Mk model.

### Sensitivity Analyses

Assignment of habitat to the Early Jurassic *Calsoyasuchus valliceps* is somewhat difficult. *Calsoyasuchus* is found in fluvial deposits of the Kayenta Formation of Arizona, which contain both terrestrial and river-dwelling vertebrates^52^. The skull of *Calsoyasuchus* is broadly similar to those of freshwater semiaquatic crocodyliforms (elongate snout, orbits relatively dorsally positioned), yet it also possesses several features characteristic of terrestrial forms, such as a dorsoventrally tall cranium and serrated dentition. To test the impact of the habitat assignment of this taxon on our results we ran separate analyses with *Calsoyasuchus* designated as terrestrial and as freshwater semiaquatic.

To test the effect of phylogenetic uncertainty on the pattern of habitat shifts, we also performed a number of analyses based on alternative tree topologies. Our phylogenetic analysis recovered *Calsoyasuchus* as the sister taxon to *Hsisosuchus* – to our knowledge, a novel topology. All prior published analyses recovered *Calsoyasuchus* as a goniopholidid. Thus, we also reran our analysis with *Calsoyasuchus* as the basal-most member of Goniopholididae (as in refs^30,32^). As noted earlier, the phylogenetic position of Thalattosuchia is contentious. To test the robustness of our results to the phylogenetic position of Thalattosuchia, we also performed our analyses on trees in which Thalattosuchia is sister to other marine neosuchians (Tethysuchia), or as basal mesoeucrocodylians (sister-group to Metasuchia as in refs^3,21^). *Stolokrosuchus* is sometimes recovered as more closely related to Neosuchia than to Notosuchia. Thus, we ran our analyses on a tree in which *Stolokrosuchus* is the sister-taxon to Neosuchia (as in ref.^20^). Finally, molecular analyses of crocodylian phylogeny consistently recover *Gavialis* as sister to *Tomistoma*, rather than as the basal-most lineage of the group. To test the effect of the molecular hypothesis on our results, we ran analyses in which *Gavialis* and *Tomistoma* are extant sister taxa, one excluding thoracosaurs and another including them. Exclusion of thoracosaurs reflects the temporal results of most molecular analyses; Late Cretaceous through Paleocene thoracosaurs predate the divergence time between *Gavialis* and *Tomistoma* supported by most molecular analyses (e.g., refs^53–55^). Combined analyses of molecular and morphological data (e.g., refs^56,57^) support a gavialoid affinity for thoracosaurs. Time calibrated tree files for sensitivity analyses are available in the Supplementary Information.

## Results

For ancestral state reconstructions, only the most likely state will be noted unless its likelihood is less than 90%. See Supplementary Information for node by node likelihood scores.

### Ancestral habitat reconstruction

**Figure 2:**
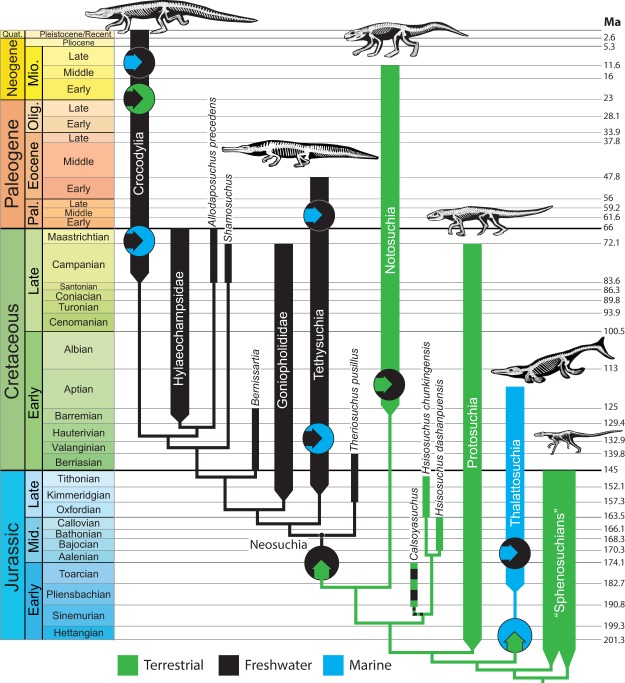
Phylogenetic and temporal pattern of habitat shifts within Crocodylomorpha. Colored arrows within circles indicate habitat transitions. Horizontal arrows indicate habitat transitions that occurred within collapsed groups. Combined green and black coloring of *Calsoyasuchus* represents uncertainty regarding its assigned habitat. Note that internal branches are scaled to make the figure legible, not according to the chronograms we employed in the analysis.

Reconstructing ancestral habitats across the tree results in a large number of independent transitions between environments (Fig. 2). Crocodylomorphs made the transition from terrestrial to aquatic habitats at least three times: (1) at the origin of Thalattosuchia in the Early Jurassic; (2) at the origin of Neosuchia in the Middle/Late Jurassic; (3) at the origin of the clade *Stolokrosuchus* + Mahajangasuchidae (within the otherwise terrestrial clade Notosuchia) in the Cretaceous. If *Calsoyasuchus* is, in fact, freshwater semiaquatic, this would require a fourth transition from terrestrial to freshwater in the Early Jurassic.

**Figure 3:**
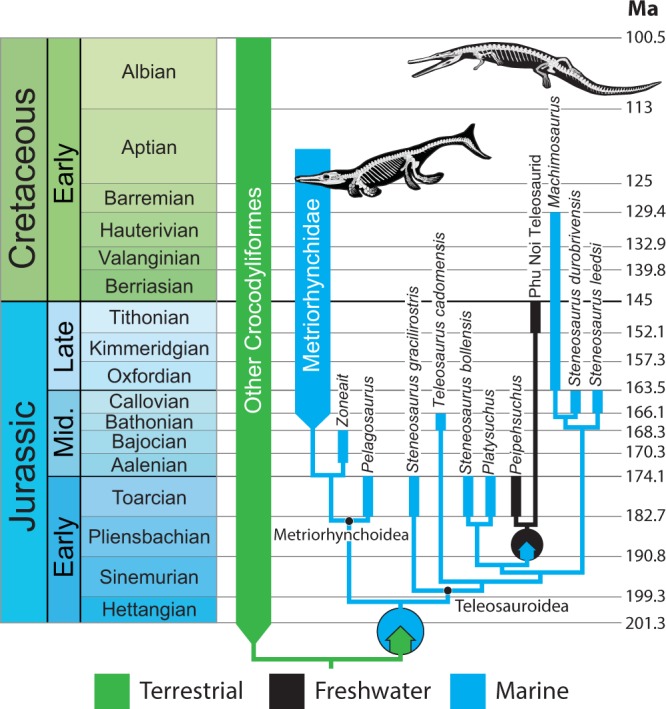
Phylogenetic and temporal pattern of habitat shifts within Thalattosuchia. Colored arrows within circles indicate habitat transitions. Ancestral state reconstructions at all nodes >0.9 in favor of one habitat. Note that internal branches are scaled to make the figure legible, not according to the chronograms we employed in the analysis.

At least nine shifts between freshwater and marine habitats occur throughout Crocodylomorpha (five from freshwater to marine; four from marine to freshwater; Fig. 2). These will be discussed on a clade-by-clade basis. Thalattosuchia is reconstructed with a habitat shift from terrestrial to marine at its origin (Fig. 3). A later shift from marine to freshwater occurred in the common ancestor of the *Peipehsuchus* + Phu Noi teleosaurid clade in the Early Jurassic.

**Figure 4:**
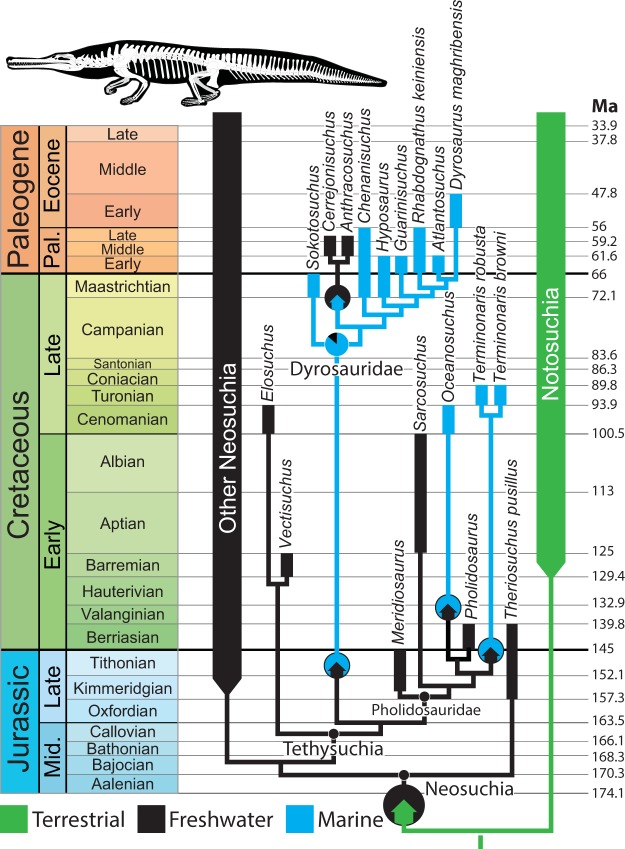
Phylogenetic and temporal pattern of habitat shifts within Tethysuchia. Colored arrows within circles indicate habitat transitions. Pie chart at Dyrosauridae node indicates relative support for each habitat (shown only for nodes where ancestral state is reconstructed with a likelihood <0.9). Note that internal branches are scaled to make the figure legible, not according to the chronograms we employed in the analysis.

The pattern of transitions within Tethysuchia is more complex, with multiple shifts from freshwater to marine habitats and one shift from marine back to freshwater (Fig. 4). Tethysuchia is reconstructed as ancestrally freshwater, with the common ancestor of Dyrosauridae reconstructed as making the transition to a marine habitat (86.0% marine; 13.3% freshwater). Within Dyrosauridae, a clade of South American taxa (*Cerrejonisuchus* + *Anthracosuchus*) reverted to a freshwater habitat. Pholidosaurids are ancestrally freshwater, and the two marine taxa (*Oceanosuchus*, *Terminonaris*) represent independent invasions of the marine realm (Fig. 4).

**Figure 5:**
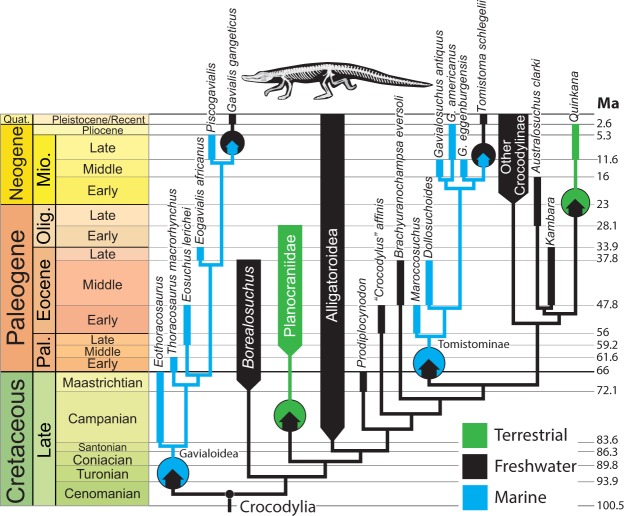
Phylogenetic and temporal pattern of habitat shifts within Crocodylia. Colored arrows within circles indicate habitat transitions. Ancestral state reconstructions at all nodes >0.9 in favor of one habitat. Note that internal branches are scaled to make the figure legible, not according to the chronograms we employed in the analysis.

Within Crocodylia, two independent transitions from freshwater to marine habitats occur: one at the base of Gavialoidea in the Late Cretaceous and one at the base of Tomistominae in the Eocene (Fig. 5). The two living representatives of these clades, *Gavialis gangeticus* and *Tomistoma schlegelii*, are limited to freshwater, representing separate reversions from marine environments.

Only twice have crocodylomorphs reverted from an aquatic to a highly terrestrial habit, both instances occurring within the crown group (Fig. 5). The planocraniids appear to have become terrestrial predators in the early Paleogene (though our phylogenetic hypothesis requires them to diverge from other crocodylian lineages in the Late Cretaceous). The second shift back to the terrestrial realm involves the Miocene mekosuchine *Quinkana*. Interestingly, we see no shifts from terrestrial back to aquatic within Neosuchia.

**Figure 6:**
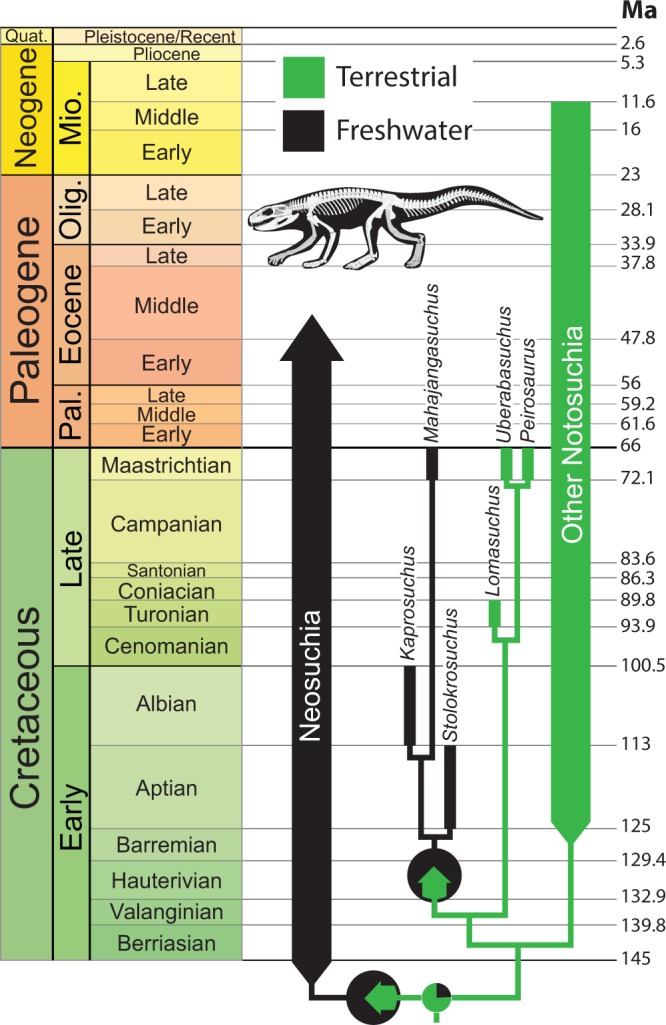
Phylogenetic and temporal pattern of habitat shifts within Notosuchia. Colored arrows within circles indicate habitat transitions. Pie chart at basal node indicates relative support for each habitat (shown only for nodes where ancestral state is reconstructed with a likelihood <0.9). Note that internal branches are scaled to make the figure legible, not according to the chronograms we employed in the analysis.

The terrestriality of Notosuchia is reconstructed as an ancestral characteristic retained from earlier crocodylomorphs (75.9% terrestrial; 23.9% freshwater; Figs 2, 6). Within Notosuchia, a single transition to freshwater occurred (at the base of the Mahajangasuchidae + *Stolokrosuchus* clade) in the Early Cretaceous (Aptian).

### Sensitivity Analyses

Results of the sensitivity analyses support the multiple-shift model (i.e. multiple shifts from terrestrial to aquatic). The alternative phylogenetic hypotheses tested result in slight differences in the timing and pattern of shifts, but only one supports the single-shift model (while implying additional aquatic to terrestrial shifts). For full results of the topological sensitivity analyses, including node-by-node likelihood scores, see the Supplementary Information.

To test the impact of habitat assignment for *Calsoyasuchus*, we ran separate analyses for terrestrial and freshwater. Scoring *Calsoyasuchus* as freshwater semiaquatic impacted some nearby nodes. The node bounding Mesoeucrocodylia + *Calsoyasuchus*/*Hsisosuchus* was still reconstructed as terrestrial, but with lower likelihood (78.8% terrestrial; 21.1% freshwater). The freshwater semiaquatic habitat assignment of *Calsoyasuchus* also affected the node bounding Neosuchia + Notosuchia, reducing the previously strong level of confidence in the inference of terrestriality for the node, but maintaining terrestrial as most likely (58.7% terrestrial; 41.1% freshwater).

Placing *Calsoyasuchus* as a goniopholidid results in only one major change – an additional transition from freshwater semiaquatic to terrestrial (if *Calsoyasuchus* is considered terrestrial). This would represent the only transition from freshwater to terrestrial outside of the crown group. This placement of *Calsoyasuchus* also reduces confidence in the reconstruction of a freshwater habitat at the base of Neosuchia (30.7% terrestrial; 67.3% freshwater), at the basal node of Goniopholididae (29.8% terrestrial; 70.2% freshwater), and at the node joining Goniopholididae with other neosuchians (27.6% terrestrial; 72. 4% freshwater).

If thalattosuchians are basal mesoeucrocodylians (sister to Notosuchia + Neosuchia), the number of transitions from terrestrial to aquatic is reduced to two: once at the base of the clade containing Thalattosuchia + other mesoeucrocodlyians (reconstructed as a terrestrial to marine transition) and once in mahajangasuchids. This renders habitat reconstruction at the node bounding Notosuchia + Neosuchia ambiguous, with a slight lean towards freshwater (25.7% terrestrial; 43.9% freshwater; 30.4% marine). Placing thalattosuchians as sister to other marine neosuchians (Tethysuchia) also reduces the number of terrestrial to aquatic transitions to two (one at the origin of Neosuchia and one at the origin of the *Stolokrosuchus* + Mahajangasuchidae clade). The habitat reconstruction for the node bounding Notosuchia + Neosuchia is ambiguous between terrestrial and freshwater (47.8% terrestrial; 51.8% freshwater), and confidence in the terrestriality of the common ancestor of Notosuchia is also reduced (67.8% terrestrial; 32.1% freshwater). If the common ancestor of Notosuchia inhabited freshwater, this would make the freshwater habitat of *Stolokrosuchus* + Mahajangasuchidae a retention of the ancestral notosuchian habitat, which would require the peirosaurids to make an independent transition from freshwater to terrestrial. This scenario reduces the overall number of transitions from terrestrial to freshwater to one and increases the number of aquatic to terrestrial transitions to three (one outside the crown group).

Positioning *Stolokrosuchus* as a basal neosuchian, rather than as sister to Mahajangasuchidae, has little impact on our results. The terrestriality of Notosuchia is even more strongly reconstructed as the retention of an ancestral habitat preference (88.4% terrestrial; 21.5% freshwater), and mahajangasuchids are still recovered as making an independent transition from terrestrial to freshwater.

Trees in which tomistomines and thoracosaurs are gavialoids reduce the number of freshwater to marine transitions in the crown group to one, but have no significant impact on any other reconstructed transitions. If tomistomines are gavialoids but thoracosaurs are not, we still reconstruct two independent transitions from freshwater to marine within or near the crown group (one in thoracosaurs and one at the root of Gavialidae). Under both scenarios, the freshwater habitat of modern *Gavialis* and *Tomistoma* represents the result of two independent transitions.

## Discussion

Crocodylomorphs are well known for exhibiting extensive convergent evolution, especially with respect to the cranium^11–13,34,58^. Unsurprisingly, much of this convergence accompanies ecological shifts, when relatively distantly related lineages adapt to the same habitats. Repeated invasion of the aquatic realm produced broadly convergent skull and body shapes – though each group elaborated or reduced features independently. When ancestrally semi-aquatic crocodylians (e.g., Planocraniidae) shifted back into the terrestrial realm, they developed cranial adaptations reminiscent of the large, predatory notosuchians (e.g., Baurusuchidae, Sebecidae) such as dorsoventrally tall snouts, mediolaterally compressed, serrated dentitions, and enlarged caniniform teeth.

The five crocodylomorph groups that independently transitioned into the marine realm (Thalattosuchia, Dyrosauridae, Pholidosauridae, Gavialoidea, and Tomistominae) share similar morphological adaptations primarily related to snout shape (i.e. an elongate slender snout). Interestingly, these groups are involved in two of the largest phylogenetic controversies in Crocodylomorpha: the *Gavialis/Tomistoma* debate^57,59–61^ and the “longirostrine problem” involving the phylogenetic relationship between thalattosuchians and tethysuchians^15,17,18,60,62^. This suggests potential morphological convergence related to marine adaptation may be obscuring phylogenetic signal.

Within each of the marine groups (with the possible exception of Pholidosauridae) some species returned to freshwater environments. This includes the extant *Gavialis gangeticus* and *Tomistoma schlegelii*. Although found only in freshwater settings today, both are derived independently from ancestors found in marginal marine deposits (Fig. 5). This is true whether they are distantly related (e.g., refs^59,63,64^) or living sister lineages, as supported by molecular and some morphometric data (e.g., refs^57,61,65^). Both gavialoids and tomistomines have geographic distributions that only make sense if marine barriers were crossed multiple times, including a crossing of the Atlantic during the late Paleogene by close *Gavialis* relatives^58,66^. Gavialoids may have made the transition from marine to freshwater twice, as *Gavialis* and some Neotropical gharials related to *Gryposuchus* (not included in our analysis) must have been separately derived from marginal marine ancestors^67^.

Only twice in crocodylomorph history have groups reverted from an aquatic to a terrestrial habit, both occurring within the crown group - the planocraniids and the mekosuchine *Quinkana*. The planocraniid excursion to the terrestrial realm occurs in the early Cenozoic, possibly in response to open niches provided by the end-Cretaceous mass extinction. *Quinkana* inhabited Australia and persisted until the early Quaternary in the absence of terrestrial placental predators.

Among notosuchians, only one major habitat shift occurred (terrestrial to freshwater semiaquatic). However, an additional major ecological transition occurred in terrestrial members of this clade in the absence of a habitat shift – a transition from terrestrial carnivore to omnivore/herbivore. A number of notosuchian taxa exhibit tightly occluding heterodont dentitions reminiscent of mammals (e.g., *Pakasuchus*^5^, sphagesaurids^6^). Others possessed dentitions mimicking herbivorous reptiles (e.g., *Simosuchus*^4,68^). These excursions into omnivory and herbivory occurred in the Cretaceous of Gondwana and may represent the filling of vacant ecological niches due to the relative paucity of mammals and small ornithischian dinosaurs in Gondwana during this time period^6^.

While we can infer saltwater tolerance in fossil lineages found in marine and estuarine deposits, the lack of good osteological correlates makes the assignment of said adaptations to a particular node of the tree difficult. The exception to this may be Thalattosuchia. Some metriorhynchids preserve three-dimensional endocasts of large antorbital structures interpreted as hypertrophied salt glands^69–73^. Osteological correlates related to these putative antorbital salt glands include an enlarged internal carotid system and a preorbital foramen, presumably for drainage of the gland^72,73^. Enlarged internal carotid systems exist in some form across Thalattosuchia, suggesting that salt tolerance is ancestral for the clade^74,75^, though a preorbital foramen is unique to derived metriorhynchoids (see^76^ for additional discussion of this topic).

Only two published thalattosuchians are known from freshwater deposits: *Peipehsuchus* and an unnamed taxon from Phu Noi, Thailand. Martin *et al*.^77^ performed strontium isotope ratio analyses on the undescribed fossils from Phu Noi to test the habitat of these teleosaurids. The Phu Noi teleosaurids fell within the range of other continental vertebrates, suggesting that they were long-term residents of continental freshwater environments and did not migrate between marine and freshwater habitats. *Peipehsuchus*, another Asian teleosaurid, is also reported from freshwater lacustrine deposits^78^. In our analysis, these two taxa form a clade nested well within Teleosauridae, and are thus derived from a marine ancestor (Fig. 3). This nested position of freshwater forms suggests that thalattosuchians are either ancestrally marine, skipping over an intermediate freshwater semiaquatic stage during their transition from the terrestrial to marine realm, or that specimens preserving a freshwater stage exist, but are yet to be recovered.

Our results indicate that salinity tolerance likely evolved independently in dyrosaurids and the marine pholidosaurids *Oceanosuchus* and *Terminonaris* (Fig. 4). However, it is also possible that salinity tolerance may have arisen once in their last common ancestor and that basal pholidosaurids, like modern crocodylids, possessed adaptations for salt tolerance but generally preferred freshwater habitats.

Within Crocodylia, all living non-alligatorids, including the gharials, have a keratinised tongue with salt-excreting glands. Alligatorid historical biogeography suggests a much smaller number of dispersal events across marine barriers than occurred in other crocodylian groups, consistent with the diminished salinity tolerance observed in living forms^79^. That close outgroups to Crocodylia tend to be geographically restricted^80^ suggests that the alligatorid condition is plesiomorphic. If true, this requires that salinity tolerance evolved at least twice within the crown group – once in the common ancestor of Gavialoidea and once along the stem of Crocodyloidea. Unfortunately, the lack of osteological correlates for the keratinised tongue and lingual salt glands precludes determination of whether freshwater semiaquatic stem crocodyloid taxa such as *Prodiplocynodon* or *Brachyuranochampsa* possessed these features. Salt tolerance must have evolved at least by the common ancestor of tomistomines and crocodylines.

## Conclusions

The long evolutionary history of Crocodylomorpha includes a number of independent major transitions between terrestrial, freshwater, and marine habitats. These transitions are not unidirectional. With the exception of marine to terrestrial, all possible transitions occurred at least once in the group’s history. Elucidating where these transitions occurred in the phylogenetic history of the group represents an important first step to investigating the associated phenotypic changes that accompany major habitat shifts.

## Electronic supplementary material

Supplementary Info

Supplementary data

## Data Availability

All data generated or analysed during this study are included in this published article (and its Supplementary Information files).
