# Supplementary material for: Evolutionary structure and timing of major habitat shifts in Crocodylomorpha
Source: Sci Rep. 2019 Jan 24;9:514. doi: 10.1038/s41598-018-36795-1 (PMC6346023; doi:10.1038/s41598-018-36795-1)
Supplement: Supplementary file 1 — Supplementary Info [file 41598_2018_36795_MOESM1_ESM.pdf]

Supplementary Information for:

Evolutionary structure and timing of major habitat shifts in Crocodylomorpha

Eric W. Wilberg[1*], Alan H. Turner[1], and Christopher A. Brochu[2]

[1] Department of Anatomical Sciences, Stony Brook University, Stony Brook, NY 11794

[2] Department of Earth and Environmental Sciences, University of Iowa, Iowa City, IA 52242

*eric.wilberg@stonybrook.edu

CONTENTS

I) PRIMARY ANALYSIS – Ancestral state reconstruction likelihoods
II) SENSITIVITY ANALYSES – Ancestral state reconstruction likelihoods
   a. *Calsoyasuchus* as goniopholidid
   b. *Stolokrosuchus* as basal neosuchian
   c. Thalattosuchia as sister to Mesoeucrocodylia
   d. Thalattosuchia as sister to Tethysuchia ("longirostrine clade")
   e. *Gavialis* and *Tomistoma* as living sister taxa (including thoracosaurs)
   f. *Gavialis* and *Tomistoma* as living sister taxa (excluding thoracosaurs)
III) Stratigraphic ranges for study taxa
IV) Material and references used for phylogenetic character scoring
V) Character list and state descriptions of the data matrix used in the phylogenetic analysis
VI) Supplemental References

I) PRIMARY ANALYSIS – Ancestral state reconstruction likelihoods

Primary analysis:

Reference tree:

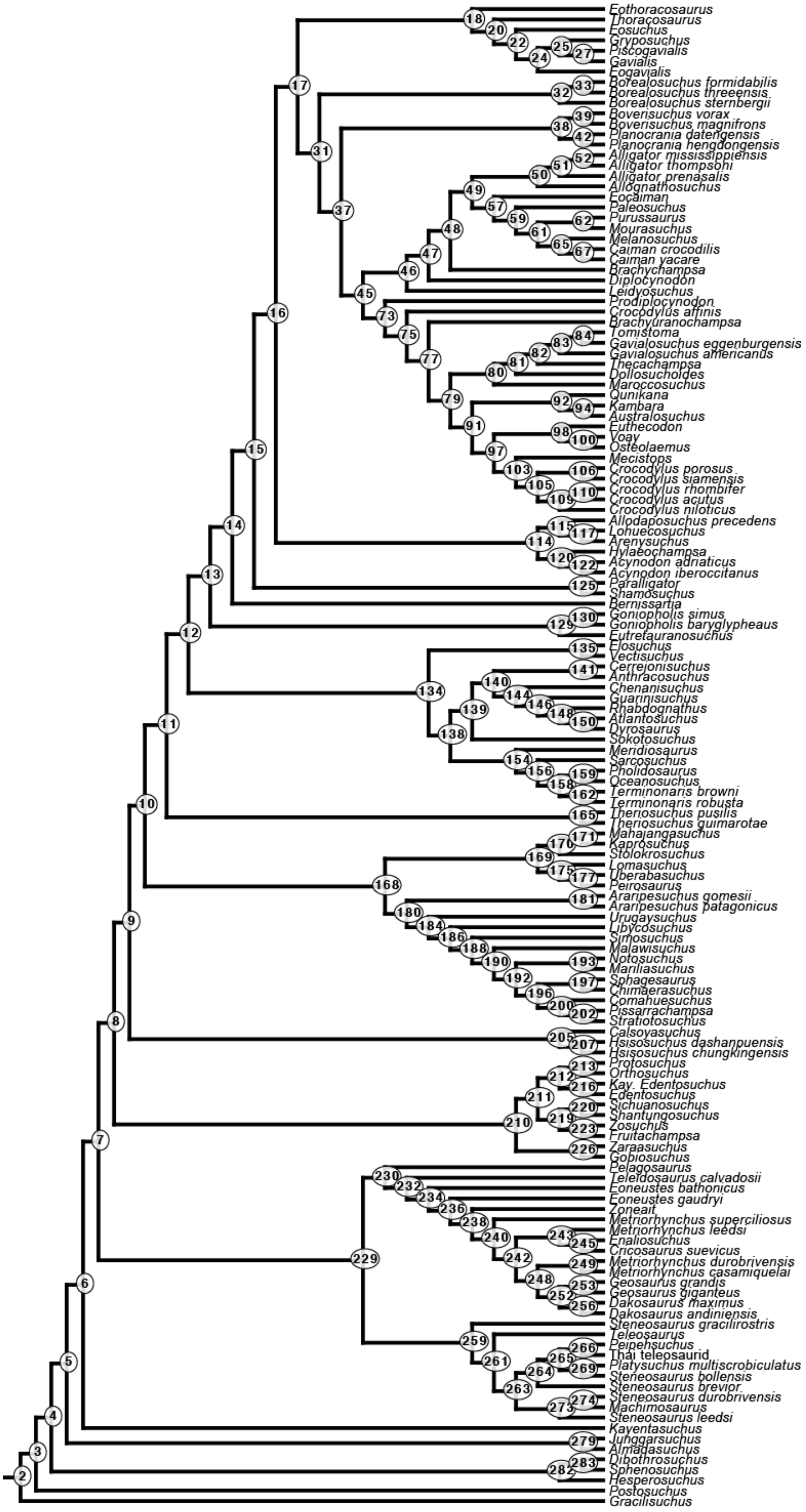

Character: habitat
Marginal prob. Recon. With model Mk1 (est.) [rate 0.00208887 [est.]] -log L.:64.53822494 (Opt.: width 0.0) Reporting likelihoods as Proportional Likelihoods; Threshold when decisions made: 2 Calc. by Maximum likelihood reconstruct (Generic categorical) (id# 1062)

node 2: 0: 0.99965248*, 1: 0.00017376, 2: 0.00017376
node 3: 0: 0.99995856*, 1: 0.00002071, 2: 0.00002072
node 4: 0: 0.99998018*, 1: 0.00000987, 2: 0.00000996
node 5: 0: 0.99997362*, 1: 0.00000738, 2: 0.000019
node 6: 0: 0.99996492*, 1: 0.00000178, 2: 0.00003329
node 7: 0: 0.9994879*, 1: 0.00001353, 2: 0.00049857
node 8: 0: 0.99998704*, 1: 0.00000285, 2: 0.00001012
node 9: 0: 0.99891708*, 1: 0.00103044, 2: 0.00005248
node 10: 0: 0.75920771*, 1: 0.23858958*, 2: 0.00220271
node 11: 0: 0.02169665, 1: 0.9780007*, 2: 0.00030265
node 12: 0: 0.0003056, 1: 0.99968233*, 2: 0.00001208
node 13: 0: 0.00001566, 1: 0.99997925*, 2: 0.00000509
node 14: 0: 0.00005058, 1: 0.99989917*, 2: 0.00005025
node 15: 0: 0.00000761, 1: 0.99998264*, 2: 0.00000976
node 16: 0: 0.00000149, 1: 0.9999903*, 2: 0.00000821
node 17: 0: 0.00075329, 1: 0.9899456*, 2: 0.00930111
node 18: 0: 0.00309794, 1: 0.0572552, 2: 0.93964686*
Eothoracosaurus: 2: 1
node 20: 0: 0.00002208, 1: 0.00013245, 2: 0.99984547*
Thoracosaurus: 2: 1
node 22: 0: 0.00000464, 1: 0.00000626, 2: 0.99998909*
Eosuchus: 2: 1
node 24: 0: 0.00002003, 1: 0.00002373, 2: 0.99995624*
node 25: 0: 0.00004719, 1: 0.0003782, 2: 0.99957462*
Gryposuchus: 2: 1
node 27: 0: 0.00025168, 1: 0.01182274, 2: 0.98792558*
Piscogavialis: 2: 1
Gavialis: 1: 1
Eogavialis: 2: 1
node 31: 0: 0.00050281, 1: 0.99882438*, 2: 0.00067281
node 32: 0: 0.00010063, 1: 0.99978807*, 2: 0.0001113
node 33: 0: 0.00004997, 1: 0.9998996*, 2: 0.00005043
Borealosuchus formidabilis: 1: 1
Borealosuchus threeensis: 1: 1
Borealosuchus sternbergii: 1: 1
node 37: 0: 0.00501645, 1: 0.99438472*, 2: 0.00059883
node 38: 0: 0.99104332*, 1: 0.00839933, 2: 0.00055735
node 39: 0: 0.99993098*, 1: 0.00005492, 2: 0.0000141
Boverisuchus vorax: 0: 1
Boverisuchus magnifrons: 0: 1
node 42: 0: 0.99984653*, 1: 0.00013968, 2: 0.00001378
Planocrania datengensis: 0: 1
Planocrania hengdongensis: 0: 1
node 45: 0: 0.00014855, 1: 0.99982399*, 2: 0.00002746
node 46: 0: 0.00001052, 1: 0.99998176*, 2: 0.00000771
node 47: 0: 0.00001141, 1: 0.99997724*, 2: 0.00001135

node 48: 0: 0.0000013, 1: 0.99999741*, 2: 0.0000013
node 49: 0: 0.00001392, 1: 0.99997215*, 2: 0.00001392
node 50: 0: 0.00000065, 1: 0.9999987*, 2: 0.00000065
node 51: 0: 0.00000213, 1: 0.99999574*, 2: 0.00000213
node 52: 0: 0.00002782, 1: 0.99994435*, 2: 0.00002782
Alligator mississippiensis: 1: 1
Alligator thompsoni: 1: 1
Alligator prenasalis: 1: 1
Allognathosuchus: 1: 1
node 57: 0: 0.00001034, 1: 0.99997931*, 2: 0.00001034
Eocaiman: 1: 1
node 59: 0: 0.00005183, 1: 0.99989634*, 2: 0.00005183
Paleosuchus: 1: 1
node 61: 0: 0.00001689, 1: 0.99996621*, 2: 0.00001689
node 62: 0: 0.00000694, 1: 0.99998612*, 2: 0.00000694
Purussaurus: 1: 1
Mourasuchus: 1: 1
node 65: 0: 0.00000498, 1: 0.99999005*, 2: 0.00000498
Melanosuchus: 1: 1
node 67: 0: 0.00000045, 1: 0.9999991*, 2: 0.00000045
Caiman crocodilis: 1: 1
Caiman yacare: 1: 1
Brachychampsa: 1: 1
Diplocynodon: 1: 1
Leidyosuchus: 1: 1
node 73: 0: 0.00003023, 1: 0.99994059*, 2: 0.00002918
Prodiplocynodon: 1: 1
node 75: 0: 0.00001169, 1: 0.99984116*, 2: 0.00014714
Crocodylus affinis: 1: 1
node 77: 0: 0.00001489, 1: 0.99909259*, 2: 0.00089252
Brachyuranochampsa: 1: 1
node 79: 0: 0.00015138, 1: 0.97994672*, 2: 0.0199019
node 80: 0: 0.00010089, 1: 0.01459404, 2: 0.98530507*
node 81: 0: 0.00000823, 1: 0.00022654, 2: 0.99976523*
node 82: 0: 0.00001449, 1: 0.00002094, 2: 0.99996457*
node 83: 0: 0.00000978, 1: 0.00010976, 2: 0.99988046*
node 84: 0: 0.00003214, 1: 0.00072867, 2: 0.99923919*
Tomistoma: 1: 1
Gavialosuchus eggenburgensis: 2: 1
Gavialosuchus americanus: 2: 1
Thecachampsa: 2: 1
Dollosuchoides: 2: 1
Maroccosuchus: 2: 1
node 91: 0: 0.00011172, 1: 0.99960602*, 2: 0.00028225
node 92: 0: 0.00071998, 1: 0.99910827*, 2: 0.00017174
Qunikana: 0: 1
node 94: 0: 0.00003358, 1: 0.99995708*, 2: 0.00000935
Kambara: 1: 1
Australosuchus: 1: 1
node 97: 0: 0.00002976, 1: 0.99993201*, 2: 0.00003823
node 98: 0: 0.00002011, 1: 0.99995944*, 2: 0.00002045

Euthecodon: 1: 1
node 100: 0: 0.00001799, 1: 0.99996401*, 2: 0.000018
Voay: 1: 1
Osteolaemus: 1: 1
node 103: 0: 0.00001417, 1: 0.99997163*, 2: 0.00001421
Mecistops: 1: 1
node 105: 0: 0.00000103, 1: 0.99999795*, 2: 0.00000103
node 106: 0: 0.00000187, 1: 0.99999626*, 2: 0.00000187
Crocodylus porosus: 1: 1
Crocodylus siamensis: 1: 1
node 109: 0: 0.00000112, 1: 0.99999775*, 2: 0.00000112
node 110: 0: 0.00000056, 1: 0.99999889*, 2: 0.00000056
Crocodylus rhombifer: 1: 1
Crocodylus acutus: 1: 1
Crocodylus niloticus: 1: 1
node 114: 0: 0.00000092, 1: 0.9999978*, 2: 0.00000128
node 115: 0: 0.00017606, 1: 0.99964785*, 2: 0.00017609
Allodaposuchus precedens: 1: 1
node 117: 0: 0.00007662, 1: 0.99984677*, 2: 0.00007662
Lohuecosuchus: 1: 1
Arenysuchus: 1: 1
node 120: 0: 0.00000099, 1: 0.999998*, 2: 0.00000101
Hylaeochampsa: 1: 1
node 122: 0: 0.00022237, 1: 0.99955526*, 2: 0.00022237
Acynodon adriaticus: 1: 1
Acynodon iberoccitanus: 1: 1
node 125: 0: 0.00013679, 1: 0.99972626*, 2: 0.00013694
Paralligator: 1: 1
Shamosuchus: 1: 1
Bernissartia: 1: 1
node 129: 0: 0.00000308, 1: 0.99999408*, 2: 0.00000285
node 130: 0: 0.00000407, 1: 0.99999186*, 2: 0.00000407
Goniopholis simus: 1: 1
Goniopholis baryglypheaus: 1: 1
Eutretauranosuchus: 1: 1
node 134: 0: 0.00004395, 1: 0.99988199*, 2: 0.00007406
node 135: 0: 0.00022471, 1: 0.99954775*, 2: 0.00022754
Elosuchus: 1: 1
Vectisuchus: 1: 1
node 138: 0: 0.00016678, 1: 0.9985408*, 2: 0.00129242
node 139: 0: 0.00769593, 1: 0.13271231*, 2: 0.85959176*
node 140: 0: 0.00019401, 1: 0.08296186, 2: 0.91684413*
node 141: 0: 0.00008345, 1: 0.98983588*, 2: 0.01008067
Cerrejonisuchus: 1: 1
Anthracosuchus: 1: 1
node 144: 0: 0.0000024, 1: 0.00073853, 2: 0.99925907*
Chenanisuchus: 2: 1
node 146: 0: 0.00000003, 1: 0.00000486, 2: 0.99999511*
Guarinisuchus: 2: 1
node 148: 0: 2.8660472E-9, 1: 0.00000002, 2: 0.99999998*
Rhabdognathus: 2: 1

node 150: 0: 0.00000021, 1: 0.00000021, 2: 0.99999957*
Atlantosuchus: 2: 1
Dyrosaurus: 2: 1
Sokotosuchus: 2: 1
node 154: 0: 0.00000624, 1: 0.99988567*, 2: 0.00010809
Meridiosaurus: 1: 1
node 156: 0: 0.00002336, 1: 0.99884471*, 2: 0.00113193
Sarcosuchus: 1: 1
node 158: 0: 0.00004623, 1: 0.99605007*, 2: 0.0039037
node 159: 0: 0.00004055, 1: 0.99626765*, 2: 0.00369181
Pholidosaurus: 1: 1
Oceanosuchus: 2: 1
node 162: 0: 0.00325708, 1: 0.05523294, 2: 0.94150998*
Terminonaris browni: 2: 1
Terminonaris robusta: 2: 1
node 165: 0: 0.00107128, 1: 0.9988485*, 2: 0.00008022
Theriosuchus pusilis: 1: 1
Theriosuchus guimarotae: 1: 1
node 168: 0: 0.94820757*, 1: 0.05143099, 2: 0.00036144
node 169: 0: 0.90164117*, 1: 0.09696332, 2: 0.00139551
node 170: 0: 0.03741714, 1: 0.96157873*, 2: 0.00100412
node 171: 0: 0.00116937, 1: 0.99870163*, 2: 0.000129
Mahajangasuchus: 1: 1
Kaprosuchus: 1: 1
Stolokrosuchus: 1: 1
node 175: 0: 0.99258078*, 1: 0.0068294, 2: 0.00058981
Lomasuchus: 0: 1
node 177: 0: 0.99986658*, 1: 0.00008727, 2: 0.00004616
Uberabasuchus: 0: 1
Peirosaurus: 0: 1
node 180: 0: 0.99798316*, 1: 0.00197871, 2: 0.00003814
node 181: 0: 0.99971771*, 1: 0.00019802, 2: 0.00008427
Araripesuchus gomesii: 0: 1
Araripesuchus patagonicus: 0: 1
node 184: 0: 0.99983511*, 1: 0.00015237, 2: 0.00001252
Urugaysuchus: 0: 1
node 186: 0: 0.99996724*, 1: 0.0000221, 2: 0.00001066
Libycosuchus: 0: 1
node 188: 0: 0.99996762*, 1: 0.00001686, 2: 0.00001552
Simosuchus: 0: 1
node 190: 0: 0.99999665*, 1: 0.00000169, 2: 0.00000167
Malawisuchus: 0: 1
node 192: 0: 0.99999653*, 1: 0.00000174, 2: 0.00000174
node 193: 0: 0.99987129*, 1: 0.00006435, 2: 0.00006435
Notosuchus: 0: 1
Mariliasuchus: 0: 1
node 196: 0: 0.99999905*, 1: 0.00000047, 2: 0.00000047
node 197: 0: 0.99999814*, 1: 0.00000093, 2: 0.00000093
Sphagesaurus: 0: 1
Chimaerasuchus: 0: 1
node 200: 0: 0.9999208*, 1: 0.0000396, 2: 0.0000396

Comahuesuchus: 0: 1
node 202: 0: 0.99999352*, 1: 0.00000324, 2: 0.00000324
Pissarrachampsa: 0: 1
Stratiotosuchus: 0: 1
node 205: 0: 0.99994807*, 1: 0.00004385, 2: 0.00000808
Calsoyasuchus: 0: 1
node 207: 0: 0.99990128*, 1: 0.00004995, 2: 0.00004878
Hsisosuchus dashanpuensis: 0: 1
Hsisosuchus chungkingensis: 0: 1
node 210: 0: 0.99999425*, 1: 0.00000235, 2: 0.0000034
node 211: 0: 0.99999877*, 1: 0.00000059, 2: 0.00000064
node 212: 0: 0.99999966*, 1: 0.00000017, 2: 0.00000017
node 213: 0: 0.99999975*, 1: 0.00000012, 2: 0.00000012
Protosuchus: 0: 1
Orthosuchus: 0: 1
node 216: 0: 0.99994631*, 1: 0.00002684, 2: 0.00002684
Kay. *Edentosuchus*: 0: 1
Edentosuchus: 0: 1
node 219: 0: 0.9996234*, 1: 0.0001883, 2: 0.0001883
node 220: 0: 0.99980643*, 1: 0.00009678, 2: 0.00009678
Sichuanosuchus: 0: 1
Shantungosuchus: 0: 1
node 223: 0: 0.99935586*, 1: 0.00032207, 2: 0.00032207
Zosuchus: 0: 1
Fruitachampsa: 0: 1
node 226: 0: 0.99443031*, 1: 0.00278479, 2: 0.0027849
Zaraasuchus: 0: 1
Gobiosuchus: 0: 1
node 229: 0: 0.00280105, 1: 0.00006953, 2: 0.99712942*
node 230: 0: 0.00005902, 1: 0.00000677, 2: 0.99993421*
Pelagosaurus: 2: 1
node 232: 0: 0.00000265, 1: 0.00000235, 2: 0.999995*
Teleidosaurus calvadosii: 2: 1
node 234: 0: 0.00000057, 1: 0.00000057, 2: 0.99999886*
Eoneustes bathonicus: 2: 1
node 236: 0: 0.00000042, 1: 0.00000042, 2: 0.99999915*
Eoneustes gaudryi: 2: 1
node 238: 0: 0.00000016, 1: 0.00000016, 2: 0.99999967*
Zoneait: 2: 1
node 240: 0: 0.00000083, 1: 0.00000083, 2: 0.99999834*
Metriorhynchus superciliosus: 2: 1
node 242: 0: 0.00000018, 1: 0.00000018, 2: 0.99999965*
node 243: 0: 0.00000201, 1: 0.00000201, 2: 0.99999599*
Metriorhynchus leedsi: 2: 1
node 245: 0: 0.00000233, 1: 0.00000233, 2: 0.99999534*
Enaliosuchus: 2: 1
Cricosaurus suevicus: 2: 1
node 248: 0: 0.00000026, 1: 0.00000026, 2: 0.99999947*
node 249: 0: 0.00000006, 1: 0.00000006, 2: 0.99999989*
Metriorhynchus durobrivensis: 2: 1
Metriorhynchus casamiquelai: 2: 1

node 252: 0: 0.000002, 1: 0.000002, 2: 0.999996*
node 253: 0: 0.00000113, 1: 0.00000113, 2: 0.99999774*
Geosaurus grandis: 2: 1
Geosaurus giganteus: 2: 1
node 256: 0: 0.00000236, 1: 0.00000236, 2: 0.99999528*
Dakosaurus maximus: 2: 1
Dakosaurus andiniensis: 2: 1
node 259: 0: 0.00009692, 1: 0.00000348, 2: 0.9998996*
Steneosaurus gracilirostris: 2: 1
node 261: 0: 0.00000806, 1: 0.00000169, 2: 0.99999025*
Teleosaurus: 2: 1
node 263: 0: 0.00000056, 1: 0.00000083, 2: 0.99999861*
node 264: 0: 0.00000059, 1: 0.00001911, 2: 0.99998031*
node 265: 0: 0.00001906, 1: 0.00143251, 2: 0.99854843*
node 266: 0: 0.00028819, 1: 0.977589*, 2: 0.02212282
Peipehsuchus: 1: 1
Thai teleosaurid: 1: 1
node 269: 0: 0.00000019, 1: 0.00000626, 2: 0.99999355*
Platysuchus multiscrobiculatus: 2: 1
Steneosaurus bollensis: 2: 1
Steneosaurus brevior: 2: 1
node 273: 0: 0.00001574, 1: 0.00001575, 2: 0.99996851*
node 274: 0: 0.00001607, 1: 0.00001607, 2: 0.99996786*
Steneosaurus durobrivensis: 2: 1
Machimosaurus: 2: 1
Steneosaurus leedsi: 2: 1
Kayentasuchus: 0: 1
node 279: 0: 0.99965644*, 1: 0.00017146, 2: 0.0001721
Junggarsuchus: 0: 1
Almadasuchus: 0: 1
node 282: 0: 0.9999817*, 1: 0.00000915, 2: 0.00000915
node 283: 0: 0.99996625*, 1: 0.00001687, 2: 0.00001687
Dibothrosuchus: 0: 1
Sphenosuchus: 0: 1
Hesperosuchus: 0: 1
Postosuchus: 0: 1
Gracilisuchus: 0: 1

II) SENSITIVITY ANALYSES – Ancestral state reconstruction likelihoods
a) *Calsoyasuchus* as goniopholidid:

Reference tree:

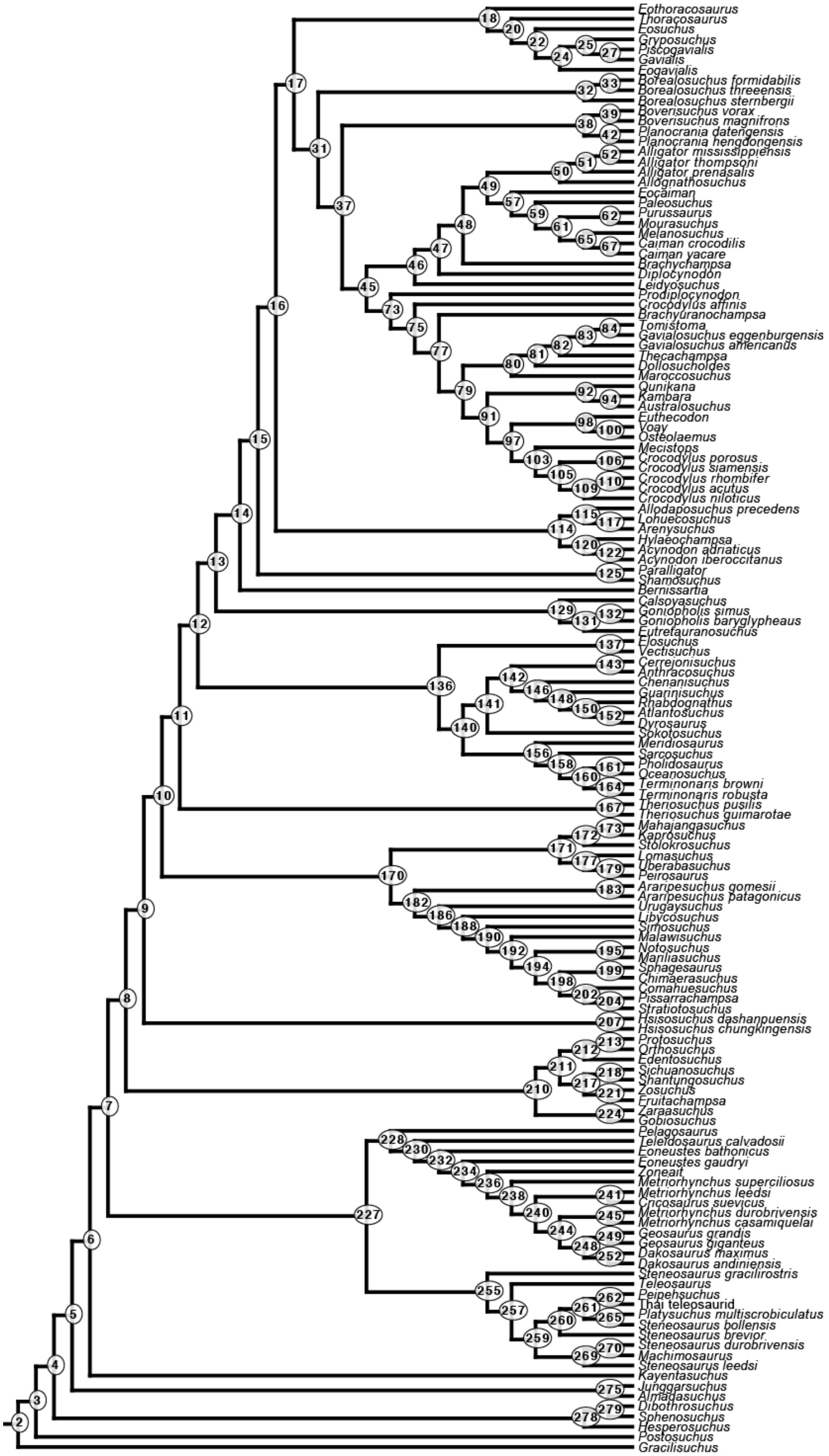

Character: habitat
Marginal prob. recon. with model Mk1 (est.) [rate 0.00217389 [est.]] -log L.:71.9053554 (Opt.: width 0.0) Reporting likelihoods as Proportional Likelihoods; Threshold when decisions made: 2
Calc. by Maximum likelihood reconstruct (Generic categorical) (id# 2303)

node 2: 0: 0.99962264*, 1: 0.00018868, 2: 0.00018868
node 3: 0: 0.99995315*, 1: 0.00002342, 2: 0.00002343
node 4: 0: 0.99997751*, 1: 0.00001119, 2: 0.0000113
node 5: 0: 0.99996955*, 1: 0.00000861, 2: 0.00002185
node 6: 0: 0.99995985*, 1: 0.0000026, 2: 0.00003755
node 7: 0: 0.99944449*, 1: 0.00002278, 2: 0.00053273
node 8: 0: 0.99995838*, 1: 0.00001976, 2: 0.00002186
node 9: 0: 0.98770459*, 1: 0.01223429, 2: 0.00006112
node 10: 0: 0.94118801*, 1: 0.05870573, 2: 0.00010627
node 11: 0: 0.30705826*, 1: 0.69282959*, 2: 0.00011214
node 12: 0: 0.27550807*, 1: 0.72446519*, 2: 0.00002675
node 13: 0: 0.27600012*, 1: 0.72397304*, 2: 0.00002684
node 14: 0: 0.01554328, 1: 0.98323939*, 2: 0.00121733
node 15: 0: 0.00006176, 1: 0.99991273*, 2: 0.00002551
node 16: 0: 0.00000521, 1: 0.99998171*, 2: 0.00001308
node 17: 0: 0.00081936, 1: 0.98949276*, 2: 0.00968789
node 18: 0: 0.00335379, 1: 0.05946583, 2: 0.93718037*
Eothoracosaurus: 2: 1
node 20: 0: 0.00002488, 1: 0.00014355, 2: 0.99983157*
Thoracosaurus: 2: 1
node 22: 0: 0.00000526, 1: 0.00000708, 2: 0.99998766*
Eosuchus: 2: 1
node 24: 0: 0.00002265, 1: 0.00002681, 2: 0.99995054*
node 25: 0: 0.00005327, 1: 0.0004113, 2: 0.99953543*
Gryposuchus: 2: 1
node 27: 0: 0.00027299, 1: 0.01231754, 2: 0.98740947*
Piscogavialis: 2: 1
Gavialis: 1: 1
Eogavialis: 2: 1
node 31: 0: 0.00054815, 1: 0.99872115*, 2: 0.00073071
node 32: 0: 0.00011404, 1: 0.99976001*, 2: 0.00012595
node 33: 0: 0.00005666, 1: 0.99988614*, 2: 0.0000572
Borealosuchus formidabilis: 1: 1
Borealosuchus threeensis: 1: 1
Borealosuchus sternbergii: 1: 1
node 37: 0: 0.00524677, 1: 0.99410296*, 2: 0.00065027
node 38: 0: 0.99065274*, 1: 0.00874262, 2: 0.00060464
node 39: 0: 0.99992419*, 1: 0.0000599, 2: 0.00001591
Boverisuchus vorax: 0: 1
Boverisuchus magnifrons: 0: 1
node 42: 0: 0.99983306*, 1: 0.0001514, 2: 0.00001555
Planocrania datengensis: 0: 1
Planocrania hengdongensis: 0: 1
node 45: 0: 0.00016234, 1: 0.99980657*, 2: 0.00003109
node 46: 0: 0.00001192, 1: 0.99997934*, 2: 0.00000875
node 47: 0: 0.0000129, 1: 0.99997425*, 2: 0.00001284

node 48: 0: 0.00000148, 1: 0.99999704*, 2: 0.00000148
node 49: 0: 0.00001574, 1: 0.99996851*, 2: 0.00001574
node 50: 0: 0.00000073, 1: 0.99999854*, 2: 0.00000073
node 51: 0: 0.00000241, 1: 0.99999518*, 2: 0.00000241
node 52: 0: 0.00003142, 1: 0.99993715*, 2: 0.00003142
Alligator mississippiensis: 1: 1
Alligator thompsoni: 1: 1
Alligator prenasalis: 1: 1
Allognathosuchus: 1: 1
node 57: 0: 0.00001172, 1: 0.99997656*, 2: 0.00001172
Eocaiman: 1: 1
node 59: 0: 0.00005868, 1: 0.99988265*, 2: 0.00005868
Paleosuchus: 1: 1
node 61: 0: 0.0000192, 1: 0.9999616*, 2: 0.0000192
node 62: 0: 0.00000785, 1: 0.9999843*, 2: 0.00000785
Purussaurus: 1: 1
Mourasuchus: 1: 1
node 65: 0: 0.00000562, 1: 0.99998876*, 2: 0.00000562
Melanosuchus: 1: 1
node 67: 0: 0.00000051, 1: 0.99999898*, 2: 0.00000051
Caiman crocodilis: 1: 1
Caiman yacare: 1: 1
Brachychampsa: 1: 1
Diplocynodon: 1: 1
Leidyosuchus: 1: 1
node 73: 0: 0.0000342, 1: 0.99993266*, 2: 0.00003315
Prodiplocynodon: 1: 1
node 75: 0: 0.00001336, 1: 0.99982037*, 2: 0.00016627
Crocodylus affinis: 1: 1
node 77: 0: 0.00001691, 1: 0.99900952*, 2: 0.00097357
Brachyuranochampsa: 1: 1
node 79: 0: 0.00016435, 1: 0.97908685*, 2: 0.0207488
node 80: 0: 0.00010933, 1: 0.01517955, 2: 0.98471113*
node 81: 0: 0.00000929, 1: 0.00024552, 2: 0.99974519*
node 82: 0: 0.00001638, 1: 0.00002363, 2: 0.99995999*
node 83: 0: 0.00001104, 1: 0.00011927, 2: 0.99986968*
node 84: 0: 0.00003503, 1: 0.00076261, 2: 0.99920236*
Tomistoma: 1: 1
Gavialosuchus eggenburgensis: 2: 1
Gavialosuchus americanus: 2: 1
Thecachampsa: 2: 1
Dollosuchoides: 2: 1
Maroccosuchus: 2: 1
node 91: 0: 0.00012176, 1: 0.99957096*, 2: 0.00030728
node 92: 0: 0.00075557, 1: 0.99905743*, 2: 0.00018699
Qunikana: 0: 1
node 94: 0: 0.00003669, 1: 0.99995273*, 2: 0.00001057
Kambara: 1: 1
Australosuchus: 1: 1
node 97: 0: 0.0000337, 1: 0.99992301*, 2: 0.00004329
node 98: 0: 0.00002277, 1: 0.99995406*, 2: 0.00002317

Euthecodon: 1: 1
node 100: 0: 0.00002032, 1: 0.99995935*, 2: 0.00002033
Voay: 1: 1
Osteolaemus: 1: 1
node 103: 0: 0.00001601, 1: 0.99996793*, 2: 0.00001606
Mecistops: 1: 1
node 105: 0: 0.00000117, 1: 0.99999767*, 2: 0.00000117
node 106: 0: 0.00000211, 1: 0.99999578*, 2: 0.00000211
Crocodylus porosus: 1: 1
Crocodylus siamensis: 1: 1
node 109: 0: 0.00000127, 1: 0.99999746*, 2: 0.00000127
node 110: 0: 0.00000063, 1: 0.99999874*, 2: 0.00000063
Crocodylus rhombifer: 1: 1
Crocodylus acutus: 1: 1
Crocodylus niloticus: 1: 1
node 114: 0: 0.00000125, 1: 0.99999707*, 2: 0.00000168
node 115: 0: 0.00019938, 1: 0.99960122*, 2: 0.0001994
Allodaposuchus precedens: 1: 1
node 117: 0: 0.0000869, 1: 0.9998262*, 2: 0.0000869
Lohuecosuchus: 1: 1
Arenysuchus: 1: 1
node 120: 0: 0.00000113, 1: 0.99999771*, 2: 0.00000116
Hylaeochampsa: 1: 1
node 122: 0: 0.00025139, 1: 0.99949722*, 2: 0.00025139
Acynodon adriaticus: 1: 1
Acynodon iberoccitanus: 1: 1
node 125: 0: 0.00015867, 1: 0.99968528*, 2: 0.00015605
Paralligator: 1: 1
Shamosuchus: 1: 1
Bernissartia: 1: 1
node 129: 0: 0.2979241*, 1: 0.70199717*, 2: 0.00007873
Calsoyasuchus: 0: 1
node 131: 0: 0.01455603, 1: 0.98497608*, 2: 0.00046789
node 132: 0: 0.00073095, 1: 0.99919262*, 2: 0.00007643
Goniopholis simus: 1: 1
Goniopholis baryglypheaus: 1: 1
Eutretauranosuchus: 1: 1
node 136: 0: 0.0253343, 1: 0.97156459*, 2: 0.00310111
node 137: 0: 0.00333169, 1: 0.9956977*, 2: 0.00097061
Elosuchus: 1: 1
Vectisuchus: 1: 1
node 140: 0: 0.01211214, 1: 0.98036892*, 2: 0.00751894
node 141: 0: 0.00909868, 1: 0.1168846, 2: 0.87401671*
node 142: 0: 0.00021048, 1: 0.07363549, 2: 0.92615403*
node 143: 0: 0.00009136, 1: 0.9893137*, 2: 0.01059494
Cerrejonisuchus: 1: 1
Anthracosuchus: 1: 1
node 146: 0: 0.0000026, 1: 0.00068224, 2: 0.99931516*
Chenanisuchus: 2: 1
node 148: 0: 0.00000003, 1: 0.00000467, 2: 0.9999953*
Guarinisuchus: 2: 1

node 150: 0: 3.23400856E-9, 1: 0.00000002, 2: 0.99999998*
Rhabdognathus: 2: 1
node 152: 0: 0.00000024, 1: 0.00000024, 2: 0.99999952*
Atlantosuchus: 2: 1
Dyrosaurus: 2: 1
Sokotosuchus: 2: 1
node 156: 0: 0.00035693, 1: 0.99896341*, 2: 0.00067965
Meridiosaurus: 1: 1
node 158: 0: 0.00005732, 1: 0.99779986*, 2: 0.00214282
Sarcosuchus: 1: 1
node 160: 0: 0.00006785, 1: 0.99502391*, 2: 0.00490824
node 161: 0: 0.00005303, 1: 0.99535717*, 2: 0.00458981
Pholidosaurus: 1: 1
Oceanosuchus: 2: 1
node 164: 0: 0.00352322, 1: 0.05733022, 2: 0.93914656*
Terminonaris browni: 2: 1
Terminonaris robusta: 2: 1
node 167: 0: 0.02018649, 1: 0.9787689*, 2: 0.00104461
Theriosuchus pusilis: 1: 1
Theriosuchus guimarotae: 1: 1
node 170: 0: 0.96950328*, 1: 0.03018178, 2: 0.00031494
node 171: 0: 0.89683877*, 1: 0.09923131, 2: 0.00392991
node 172: 0: 0.05773978, 1: 0.93912094*, 2: 0.00313929
node 173: 0: 0.00126616, 1: 0.99850196*, 2: 0.00023188
Mahajangasuchus: 1: 1
Kaprosuchus: 1: 1
Stolokrosuchus: 1: 1
node 177: 0: 0.98880974*, 1: 0.0096259, 2: 0.00156436
Lomasuchus: 0: 1
node 179: 0: 0.99981689*, 1: 0.00011193, 2: 0.00007118
Uberabasuchus: 0: 1
Peirosaurus: 0: 1
node 182: 0: 0.99753467*, 1: 0.00235027, 2: 0.00011506
node 183: 0: 0.99892461*, 1: 0.00063017, 2: 0.00044522
Araripesuchus gomesii: 0: 1
Araripesuchus patagonicus: 0: 1
node 186: 0: 0.99954878*, 1: 0.00036087, 2: 0.00009035
Urugaysuchus: 0: 1
node 188: 0: 0.99980571*, 1: 0.00011333, 2: 0.00008095
Libycosuchus: 0: 1
node 190: 0: 0.99980576*, 1: 0.00009939, 2: 0.00009485
Simosuchus: 0: 1
node 192: 0: 0.99996944*, 1: 0.00001534, 2: 0.00001523
Malawisuchus: 0: 1
node 194: 0: 0.99999278*, 1: 0.00000361, 2: 0.00000361
node 195: 0: 0.99985442*, 1: 0.00007279, 2: 0.00007279
Notosuchus: 0: 1
Mariliasuchus: 0: 1
node 198: 0: 0.9999988*, 1: 0.0000006, 2: 0.0000006
node 199: 0: 0.99999788*, 1: 0.00000106, 2: 0.00000106
Sphagesaurus: 0: 1

Chimaerasuchus: 0: 1
node 202: 0: 0.99991055*, 1: 0.00004473, 2: 0.00004473
Comahuesuchus: 0: 1
node 204: 0: 0.99999268*, 1: 0.00000366, 2: 0.00000366
Pissarrachampsa: 0: 1
Stratiotosuchus: 0: 1
node 207: 0: 0.99908194*, 1: 0.00081293, 2: 0.00010513
Hsisosuchus dashanpuensis: 0: 1
Hsisosuchus chungkingensis: 0: 1
node 210: 0: 0.9999892*, 1: 0.00000524, 2: 0.00000556
node 211: 0: 0.99999809*, 1: 0.00000095, 2: 0.00000096
node 212: 0: 0.99999436*, 1: 0.00000282, 2: 0.00000282
node 213: 0: 0.99999967*, 1: 0.00000017, 2: 0.00000017
Protosuchus: 0: 1
Orthosuchus: 0: 1
Edentosuchus: 0: 1
node 217: 0: 0.99957085*, 1: 0.00021457, 2: 0.00021458
node 218: 0: 0.99978107*, 1: 0.00010946, 2: 0.00010946
Sichuanosuchus: 0: 1
Shantungosuchus: 0: 1
node 221: 0: 0.9992685*, 1: 0.00036575, 2: 0.00036575
Zosuchus: 0: 1
Fruitachampsa: 0: 1
node 224: 0: 0.99369408*, 1: 0.00315294, 2: 0.00315298
Zaraasuchus: 0: 1
Gobiosuchus: 0: 1
node 227: 0: 0.00292121, 1: 0.00007552, 2: 0.99700326*
node 228: 0: 0.00006425, 1: 0.00000764, 2: 0.99992811*
Pelagosaurus: 2: 1
node 230: 0: 0.000003, 1: 0.00000266, 2: 0.99999435*
Teleidosaurus calvadosii: 2: 1
node 232: 0: 0.00000065, 1: 0.00000064, 2: 0.99999871*
Eoneustes bathonicus: 2: 1
node 234: 0: 0.00000048, 1: 0.00000048, 2: 0.99999904*
Eoneustes gaudryi: 2: 1
node 236: 0: 0.00000019, 1: 0.00000019, 2: 0.99999963*
Zoneait: 2: 1
node 238: 0: 0.00000094, 1: 0.00000094, 2: 0.99999813*
Metriorhynchus superciliosus: 2: 1
node 240: 0: 0.0000002, 1: 0.0000002, 2: 0.9999996*
node 241: 0: 0.00000292, 1: 0.00000292, 2: 0.99999417*
Metriorhynchus leedsi: 2: 1
Cricosaurus suevicus: 2: 1
node 244: 0: 0.0000003, 1: 0.0000003, 2: 0.9999994*
node 245: 0: 0.00000006, 1: 0.00000006, 2: 0.99999988*
Metriorhynchus durobrivensis: 2: 1
Metriorhynchus casamiquelai: 2: 1
node 248: 0: 0.00000226, 1: 0.00000226, 2: 0.99999549*
node 249: 0: 0.00000128, 1: 0.00000128, 2: 0.99999744*
Geosaurus grandis: 2: 1
Geosaurus giganteus: 2: 1

node 252: 0: 0.00000266, 1: 0.00000266, 2: 0.99999467*
Dakosaurus maximus: 2: 1
Dakosaurus andiniensis: 2: 1
node 255: 0: 0.00010544, 1: 0.00000394, 2: 0.99989063*
Steneosaurus gracilirostris: 2: 1
node 257: 0: 0.00000912, 1: 0.00000192, 2: 0.99998896*
Teleosaurus: 2: 1
node 259: 0: 0.00000064, 1: 0.00000094, 2: 0.99999841*
node 260: 0: 0.00000066, 1: 0.00002071, 2: 0.99997863*
node 261: 0: 0.00002066, 1: 0.00149029, 2: 0.99848905*
node 262: 0: 0.0003122, 1: 0.97666602*, 2: 0.02302178
Peipehsuchus: 1: 1
Thai teleosaurid: 1: 1
node 265: 0: 0.00000021, 1: 0.00000678, 2: 0.99999301*
Platysuchus multiscrobiculatus: 2: 1
Steneosaurus bollensis: 2: 1
Steneosaurus brevior: 2: 1
node 269: 0: 0.0000178, 1: 0.00001781, 2: 0.99996439*
node 270: 0: 0.00001817, 1: 0.00001817, 2: 0.99996366*
Steneosaurus durobrivensis: 2: 1
Machimosaurus: 2: 1
Steneosaurus leedsi: 2: 1
Kayentasuchus: 0: 1
node 275: 0: 0.99961157*, 1: 0.00019384, 2: 0.0001946
Junggarsuchus: 0: 1
Almadasuchus: 0: 1
node 278: 0: 0.9999793*, 1: 0.00001035, 2: 0.00001035
node 279: 0: 0.99996189*, 1: 0.00001905, 2: 0.00001905
Dibothrosuchus: 0: 1
Sphenosuchus: 0: 1
Hesperosuchus: 0: 1
Postosuchus: 0: 1
Gracilisuchus: 0: 1

b) *Stolokrosuchus* as basal neosuchian:

Reference tree:

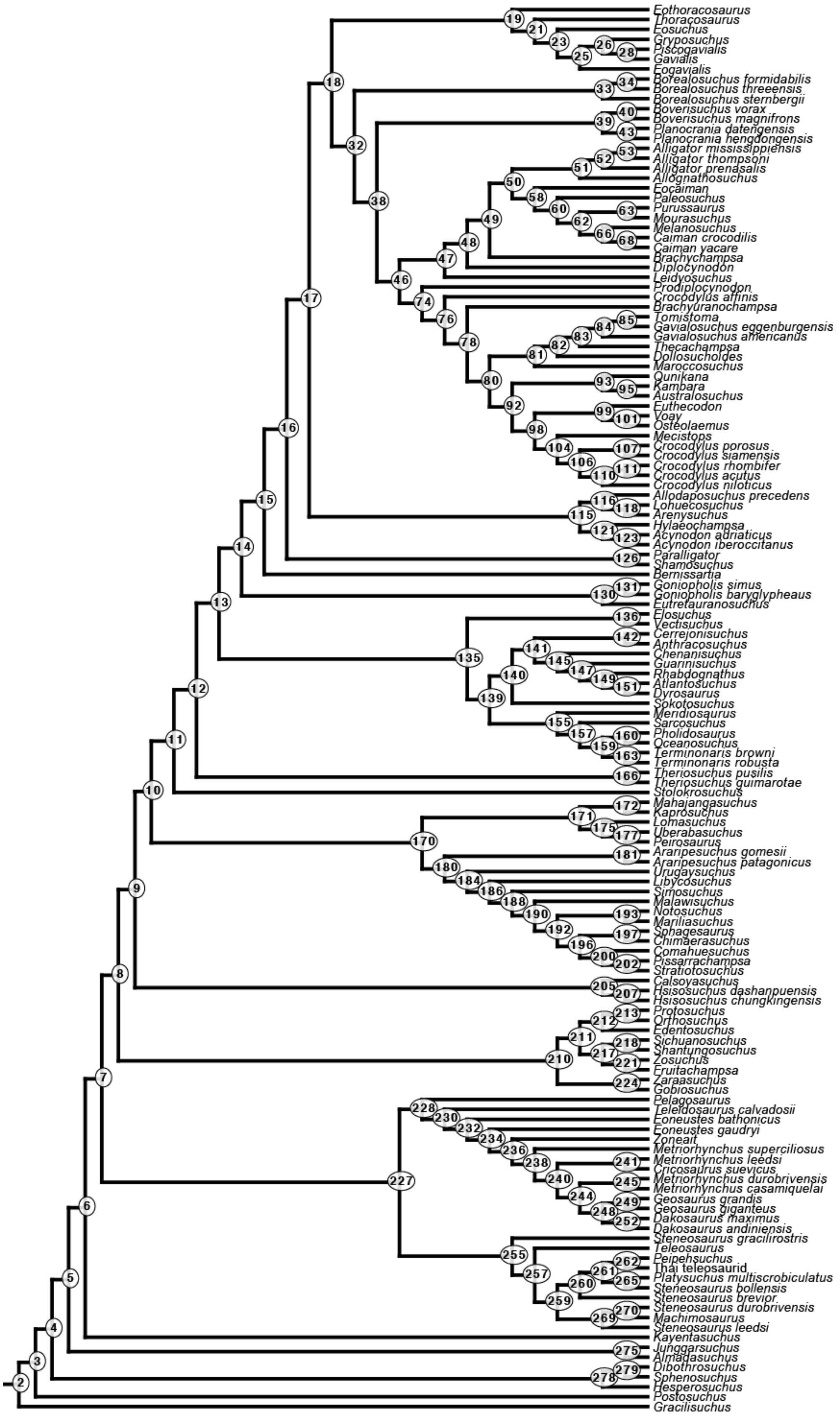

Character: habitat
Marginal prob. recon. with model Mk1 (est.) [rate 0.00201367 [est.]] -log L.:64.45339417 (Opt.: width 0.0) Reporting likelihoods as Proportional Likelihoods; Threshold when decisions made: 2
Calc. by Maximum likelihood reconstruct (Generic categorical) (id# 1578)

node 2: 0: 0.99967779*, 1: 0.0001611, 2: 0.00016111
node 3: 0: 0.99996299*, 1: 0.0000185, 2: 0.00001851
node 4: 0: 0.99998234*, 1: 0.00000879, 2: 0.00000887
node 5: 0: 0.99997655*, 1: 0.00000655, 2: 0.0000169
node 6: 0: 0.99996813*, 1: 0.00000154, 2: 0.00003033
node 7: 0: 0.99951063*, 1: 0.00001197, 2: 0.00047741
node 8: 0: 0.99999058*, 1: 0.00000148, 2: 0.00000794
node 9: 0: 0.99947455*, 1: 0.00050092, 2: 0.00002452
node 10: 0: 0.88406651*, 1: 0.11525548, 2: 0.00067801
node 11: 0: 0.11418813, 1: 0.8846689*, 2: 0.00114297
node 12: 0: 0.00274148, 1: 0.99720377*, 2: 0.00005475
node 13: 0: 0.00003354, 1: 0.99996388*, 2: 0.00000257
node 14: 0: 0.000004, 1: 0.99999301*, 2: 0.00000299
node 15: 0: 0.00003757, 1: 0.99992485*, 2: 0.00003758
node 16: 0: 0.00000638, 1: 0.99998542*, 2: 0.0000082
node 17: 0: 0.00000129, 1: 0.99999135*, 2: 0.00000736
node 18: 0: 0.00069845, 1: 0.99033874*, 2: 0.00896281
node 19: 0: 0.00287998, 1: 0.05529081, 2: 0.94182921*
Eothoracosaurus: 2: 1
node 21: 0: 0.00001979, 1: 0.000123, 2: 0.9998572*
Thoracosaurus: 2: 1
node 23: 0: 0.00000414, 1: 0.0000056, 2: 0.99999026*
Eosuchus: 2: 1
node 25: 0: 0.00001789, 1: 0.00002121, 2: 0.9999609*
node 26: 0: 0.00004221, 1: 0.00035017, 2: 0.99960762*
Gryposuchus: 2: 1
node 28: 0: 0.00023357, 1: 0.01138592, 2: 0.98838051*
Piscogavialis: 2: 1
Gavialis: 1: 1
Eogavialis: 2: 1
node 32: 0: 0.00046496, 1: 0.9989111*, 2: 0.00062394
node 33: 0: 0.00008974, 1: 0.99981089*, 2: 0.00009937
node 34: 0: 0.00004452, 1: 0.99991055*, 2: 0.00004493
Borealosuchus formidabilis: 1: 1
Borealosuchus threeensis: 1: 1
Borealosuchus sternbergii: 1: 1
node 38: 0: 0.00481498, 1: 0.99462971*, 2: 0.00055531
node 39: 0: 0.99138708*, 1: 0.00809573, 2: 0.00051719
node 40: 0: 0.99993666*, 1: 0.00005072, 2: 0.00001262
Boverisuchus vorax: 0: 1
Boverisuchus magnifrons: 0: 1
node 43: 0: 0.99985794*, 1: 0.00012972, 2: 0.00001234
Planocrania datengensis: 0: 1
Planocrania hengdongensis: 0: 1
node 46: 0: 0.00013696, 1: 0.99983853*, 2: 0.0000245
node 47: 0: 0.00000939, 1: 0.99998374*, 2: 0.00000687

node 48: 0: 0.00001019, 1: 0.99997967*, 2: 0.00001014
node 49: 0: 0.00000115, 1: 0.9999977*, 2: 0.00000115
node 50: 0: 0.00001244, 1: 0.99997512*, 2: 0.00001244
node 51: 0: 0.00000058, 1: 0.99999884*, 2: 0.00000058
node 52: 0: 0.00000191, 1: 0.99999619*, 2: 0.00000191
node 53: 0: 0.00002488, 1: 0.99995023*, 2: 0.00002488
Alligator mississippiensis: 1: 1
Alligator thompsoni: 1: 1
Alligator prenasalis: 1: 1
Allognathosuchus: 1: 1
node 58: 0: 0.00000922, 1: 0.99998155*, 2: 0.00000922
Eocaiman: 1: 1
node 60: 0: 0.00004625, 1: 0.99990751*, 2: 0.00004625
Paleosuchus: 1: 1
node 62: 0: 0.00001502, 1: 0.99996995*, 2: 0.00001502
node 63: 0: 0.0000062, 1: 0.99998761*, 2: 0.0000062
Purussaurus: 1: 1
Mourasuchus: 1: 1
node 66: 0: 0.00000445, 1: 0.9999911*, 2: 0.00000445
Melanosuchus: 1: 1
node 68: 0: 0.0000004, 1: 0.9999992*, 2: 0.0000004
Caiman crocodilis: 1: 1
Caiman yacare: 1: 1
Brachychampsa: 1: 1
Diplocynodon: 1: 1
Leidyosuchus: 1: 1
node 74: 0: 0.000027, 1: 0.99994705*, 2: 0.00002596
Prodiplocynodon: 1: 1
node 76: 0: 0.00001035, 1: 0.99985813*, 2: 0.00013152
Crocodylus affinis: 1: 1
node 78: 0: 0.00001325, 1: 0.99916257*, 2: 0.00082418
Brachyuranochampsa: 1: 1
node 80: 0: 0.00014037, 1: 0.9807041*, 2: 0.01915553
node 81: 0: 0.00009371, 1: 0.01407548, 2: 0.98583081*
node 82: 0: 0.00000737, 1: 0.00021039, 2: 0.99978224*
node 83: 0: 0.00001295, 1: 0.00001873, 2: 0.99996832*
node 84: 0: 0.00000874, 1: 0.0001017, 2: 0.99988956*
node 85: 0: 0.0000297, 1: 0.00069894, 2: 0.99927136*
Tomistoma: 1: 1
Gavialosuchus eggenburgensis: 2: 1
Gavialosuchus americanus: 2: 1
Thecachampsa: 2: 1
Dollosuchoides: 2: 1
Maroccosuchus: 2: 1
node 92: 0: 0.00010325, 1: 0.99963564*, 2: 0.0002611
node 93: 0: 0.00068895, 1: 0.99915219*, 2: 0.00015886
Qunikana: 0: 1
node 95: 0: 0.00003095, 1: 0.9999607*, 2: 0.00000834
Kambara: 1: 1
Australosuchus: 1: 1
node 98: 0: 0.00002655, 1: 0.99993934*, 2: 0.00003411

node 99: 0: 0.00001794, 1: 0.99996383*, 2: 0.00001823
Euthecodon: 1: 1
node 101: 0: 0.00001609, 1: 0.99996782*, 2: 0.00001609
Voay: 1: 1
Osteolaemus: 1: 1
node 104: 0: 0.00001266, 1: 0.99997464*, 2: 0.0000127
Mecistops: 1: 1
node 106: 0: 0.00000091, 1: 0.99999818*, 2: 0.00000091
node 107: 0: 0.00000167, 1: 0.99999665*, 2: 0.00000167
Crocodylus porosus: 1: 1
Crocodylus siamensis: 1: 1
node 110: 0: 0.00000101, 1: 0.99999799*, 2: 0.00000101
node 111: 0: 0.0000005, 1: 0.999999*, 2: 0.0000005
Crocodylus rhombifer: 1: 1
Crocodylus acutus: 1: 1
Crocodylus niloticus: 1: 1
node 115: 0: 0.00000082, 1: 0.99999806*, 2: 0.00000113
node 116: 0: 0.00015708, 1: 0.99968582*, 2: 0.0001571
Allodaposuchus precedens: 1: 1
node 118: 0: 0.00006825, 1: 0.99986349*, 2: 0.00006826
Lohuecosuchus: 1: 1
Arenysuchus: 1: 1
node 121: 0: 0.00000088, 1: 0.99999822*, 2: 0.0000009
Hylaeochampsa: 1: 1
node 123: 0: 0.00019867, 1: 0.99960265*, 2: 0.00019867
Acynodon adriaticus: 1: 1
Acynodon iberoccitanus: 1: 1
node 126: 0: 0.0001222, 1: 0.99975547*, 2: 0.00012232
Paralligator: 1: 1
Shamosuchus: 1: 1
Bernissartia: 1: 1
node 130: 0: 0.0000017, 1: 0.99999661*, 2: 0.00000168
node 131: 0: 0.00000265, 1: 0.99999469*, 2: 0.00000265
Goniopholis simus: 1: 1
Goniopholis baryglypheaus: 1: 1
Eutretauranosuchus: 1: 1
node 135: 0: 0.00001325, 1: 0.99993725*, 2: 0.00004951
node 136: 0: 0.00017625, 1: 0.99964425*, 2: 0.0001795
Elosuchus: 1: 1
Vectisuchus: 1: 1
node 139: 0: 0.00011299, 1: 0.99888737*, 2: 0.00099964
node 140: 0: 0.00713112, 1: 0.1339723*, 2: 0.85889658*
node 141: 0: 0.00018128, 1: 0.08454009, 2: 0.91527863*
node 142: 0: 0.00007736, 1: 0.99021946*, 2: 0.00970318
Cerrejonisuchus: 1: 1
Anthracosuchus: 1: 1
node 145: 0: 0.0000022, 1: 0.00072543, 2: 0.99927237*
Chenanisuchus: 2: 1
node 147: 0: 0.00000002, 1: 0.0000046, 2: 0.99999538*
Guarinisuchus: 2: 1
node 149: 0: 2.56192376E-9, 1: 0.00000002, 2: 0.99999998*

Rhabdognathus: 2: 1
node 151: 0: 0.00000019, 1: 0.00000019, 2: 0.99999962*
Atlantosuchus: 2: 1
Dyrosaurus: 2: 1
Sokotosuchus: 2: 1
node 155: 0: 0.00000389, 1: 0.99991494*, 2: 0.00008117
Meridiosaurus: 1: 1
node 157: 0: 0.00001919, 1: 0.998992*, 2: 0.00098881
Sarcosuchus: 1: 1
node 159: 0: 0.0000415, 1: 0.9961948*, 2: 0.0037637
node 160: 0: 0.00003692, 1: 0.99639143*, 2: 0.00357165
Pholidosaurus: 1: 1
Oceanosuchus: 2: 1
node 163: 0: 0.00302857, 1: 0.05332666, 2: 0.94364477*
Terminonaris browni: 2: 1
Terminonaris robusta: 2: 1
node 166: 0: 0.00014168, 1: 0.99982913*, 2: 0.00002919
Theriosuchus pusilis: 1: 1
Theriosuchus guimarotae: 1: 1
Stolokrosuchus: 1: 1
node 170: 0: 0.93720241*, 1: 0.06244971, 2: 0.00034789
node 171: 0: 0.88432713*, 1: 0.11214796, 2: 0.00352492
node 172: 0: 0.09142613, 1: 0.9032224*, 2: 0.00535147
Mahajangasuchus: 1: 1
Kaprosuchus: 1: 1
node 175: 0: 0.98982656*, 1: 0.00907289, 2: 0.00110055
Lomasuchus: 0: 1
node 177: 0: 0.99985471*, 1: 0.00009355, 2: 0.00005173
Uberabasuchus: 0: 1
Peirosaurus: 0: 1
node 180: 0: 0.99574974*, 1: 0.00412434, 2: 0.00012592
node 181: 0: 0.9990746*, 1: 0.00061258, 2: 0.00031283
Araripesuchus gomesii: 0: 1
Araripesuchus patagonicus: 0: 1
node 184: 0: 0.99943881*, 1: 0.00049391, 2: 0.00006728
Urugaysuchus: 0: 1
node 186: 0: 0.99984748*, 1: 0.00009903, 2: 0.00005349
Libycosuchus: 0: 1
node 188: 0: 0.99986755*, 1: 0.00006916, 2: 0.00006329
Simosuchus: 0: 1
node 190: 0: 0.9999803*, 1: 0.00000991, 2: 0.00000978
Malawisuchus: 0: 1
node 192: 0: 0.99999465*, 1: 0.00000267, 2: 0.00000267
node 193: 0: 0.99988481*, 1: 0.00005759, 2: 0.00005759
Notosuchus: 0: 1
Mariliasuchus: 0: 1
node 196: 0: 0.99999908*, 1: 0.00000046, 2: 0.00000046
node 197: 0: 0.99999833*, 1: 0.00000083, 2: 0.00000083
Sphagesaurus: 0: 1
Chimaerasuchus: 0: 1
node 200: 0: 0.99992917*, 1: 0.00003542, 2: 0.00003542

Comahuesuchus: 0: 1
node 202: 0: 0.9999942*, 1: 0.0000029, 2: 0.0000029
Pissarrachampsa: 0: 1
Stratiotosuchus: 0: 1
node 205: 0: 0.99997088*, 1: 0.00002296, 2: 0.00000616
Calsoyasuchus: 0: 1
node 207: 0: 0.99991237*, 1: 0.00004408, 2: 0.00004355
Hsisosuchus dashanpuensis: 0: 1
Hsisosuchus chungkingensis: 0: 1
node 210: 0: 0.99999522*, 1: 0.00000194, 2: 0.00000284
node 211: 0: 0.99999873*, 1: 0.00000061, 2: 0.00000065
node 212: 0: 0.99999559*, 1: 0.0000022, 2: 0.00000221
node 213: 0: 0.99999974*, 1: 0.00000013, 2: 0.00000013
Protosuchus: 0: 1
Orthosuchus: 0: 1
Edentosuchus: 0: 1
node 217: 0: 0.99966583*, 1: 0.00016708, 2: 0.00016709
node 218: 0: 0.99982713*, 1: 0.00008644, 2: 0.00008644
Sichuanosuchus: 0: 1
Shantungosuchus: 0: 1
node 221: 0: 0.99942682*, 1: 0.00028659, 2: 0.00028659
Zosuchus: 0: 1
Fruitachampsa: 0: 1
node 224: 0: 0.99503063*, 1: 0.00248464, 2: 0.00248473
Zaraasuchus: 0: 1
Gobiosuchus: 0: 1
node 227: 0: 0.00269515, 1: 0.00006446, 2: 0.99724039*
node 228: 0: 0.0000546, 1: 0.00000605, 2: 0.99993934*
Pelagosaurus: 2: 1
node 230: 0: 0.00000237, 1: 0.0000021, 2: 0.99999553*
Teleidosaurus calvadosii: 2: 1
node 232: 0: 0.00000051, 1: 0.0000005, 2: 0.99999899*
Eoneustes bathonicus: 2: 1
node 234: 0: 0.00000038, 1: 0.00000038, 2: 0.99999924*
Eoneustes gaudryi: 2: 1
node 236: 0: 0.00000015, 1: 0.00000015, 2: 0.99999971*
Zoneait: 2: 1
node 238: 0: 0.00000074, 1: 0.00000074, 2: 0.99999852*
Metriorhynchus superciliosus: 2: 1
node 240: 0: 0.00000016, 1: 0.00000016, 2: 0.99999969*
node 241: 0: 0.00000231, 1: 0.00000231, 2: 0.99999537*
Metriorhynchus leedsi: 2: 1
Cricosaurus suevicus: 2: 1
node 244: 0: 0.00000024, 1: 0.00000024, 2: 0.99999953*
node 245: 0: 0.00000005, 1: 0.00000005, 2: 0.9999999*
Metriorhynchus durobrivensis: 2: 1
Metriorhynchus casamiquelai: 2: 1
node 248: 0: 0.00000179, 1: 0.00000179, 2: 0.99999643*
node 249: 0: 0.00000101, 1: 0.00000101, 2: 0.99999797*
Geosaurus grandis: 2: 1
Geosaurus giganteus: 2: 1

node 252: 0: 0.00000211, 1: 0.00000211, 2: 0.99999578*
Dakosaurus maximus: 2: 1
Dakosaurus andiniensis: 2: 1
node 255: 0: 0.00008971, 1: 0.0000031, 2: 0.99990719*
Steneosaurus gracilirostris: 2: 1
node 257: 0: 0.0000072, 1: 0.0000015, 2: 0.9999913*
Teleosaurus: 2: 1
node 259: 0: 0.0000005, 1: 0.00000074, 2: 0.99999876*
node 260: 0: 0.00000052, 1: 0.00001774, 2: 0.99998174*
node 261: 0: 0.0000177, 1: 0.00138136, 2: 0.99860094*
node 262: 0: 0.00026775, 1: 0.97840499*, 2: 0.02132726
Peipehsuchus: 1: 1
Thai teleosaurid: 1: 1
node 265: 0: 0.00000017, 1: 0.00000582, 2: 0.99999401*
Platysuchus multiscrobiculatus: 2: 1
Steneosaurus bollensis: 2: 1
Steneosaurus brevior: 2: 1
node 269: 0: 0.00001406, 1: 0.00001407, 2: 0.99997187*
node 270: 0: 0.00001436, 1: 0.00001436, 2: 0.99997128*
Steneosaurus durobrivensis: 2: 1
Machimosaurus: 2: 1
Steneosaurus leedsi: 2: 1
Kayentasuchus: 0: 1
node 275: 0: 0.99969305*, 1: 0.0001532, 2: 0.00015375
Junggarsuchus: 0: 1
Almadasuchus: 0: 1
node 278: 0: 0.99998366*, 1: 0.00000817, 2: 0.00000817
node 279: 0: 0.99996982*, 1: 0.00001509, 2: 0.00001509
Dibothrosuchus: 0: 1
Sphenosuchus: 0: 1
Hesperosuchus: 0: 1
Postosuchus: 0: 1
Gracilisuchus: 0: 1

c) Thalattosuchia as sister to Mesoeucrocodylia:

Reference tree:

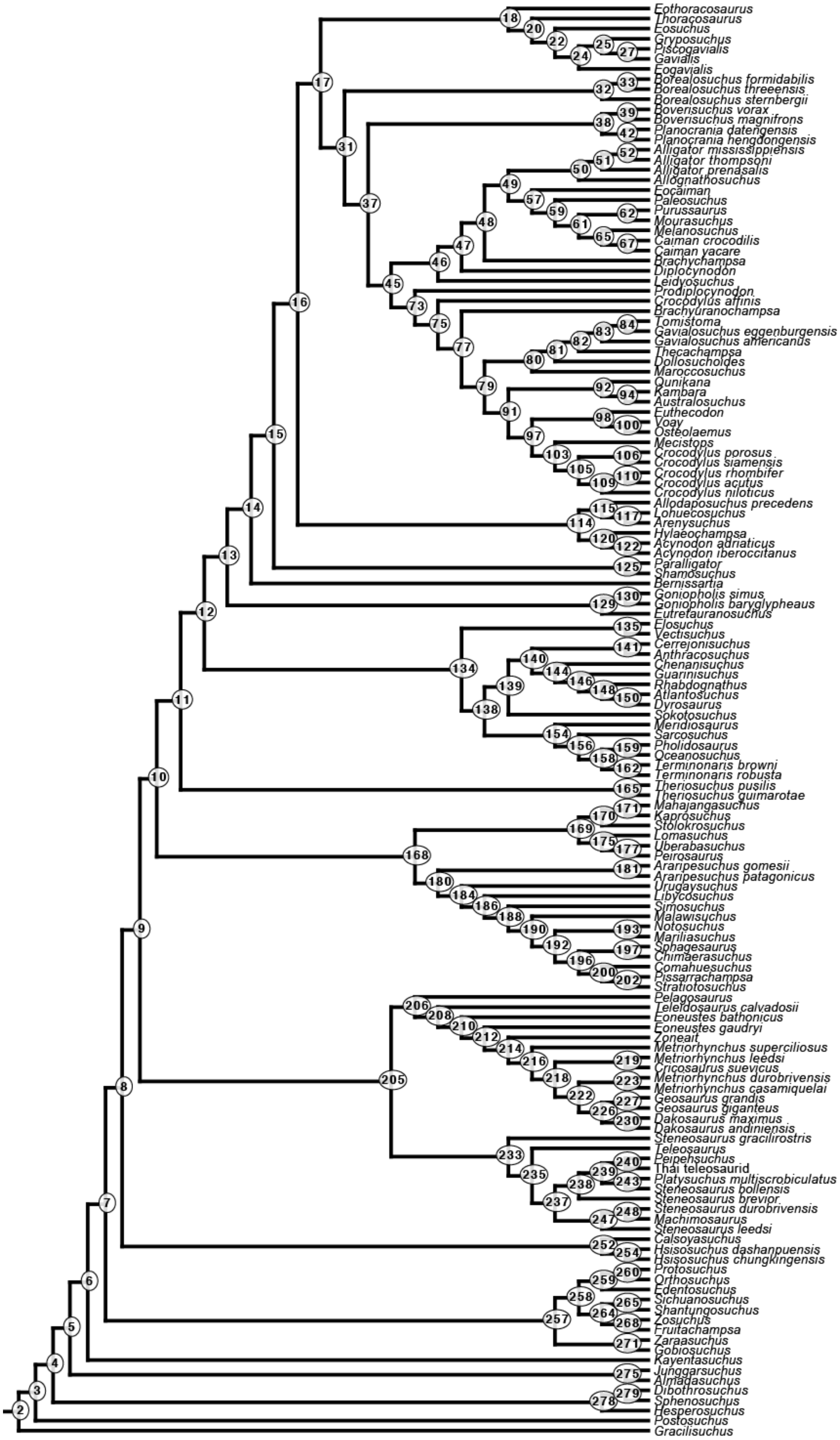

Character: habitat
Marginal prob. recon. with model Mk1 (est.) [rate 0.00223949 [est.]] -log L.:66.18342902 (Opt.: width 0.0) Reporting likelihoods as Proportional Likelihoods; Threshold when decisions made: 2
Calc. by Maximum likelihood reconstruct (Generic categorical) (id# 1757)

node 2: 0: 0.99959871*, 1: 0.00020064, 2: 0.00020064
node 3: 0: 0.99994863*, 1: 0.00002568, 2: 0.00002568
node 4: 0: 0.99997511*, 1: 0.00001245, 2: 0.00001245
node 5: 0: 0.99998008*, 1: 0.00000988, 2: 0.00001003
node 6: 0: 0.99999701*, 1: 0.00000128, 2: 0.00000171
node 7: 0: 0.99999132*, 1: 0.00000118, 2: 0.00000749
node 8: 0: 0.99476952*, 1: 0.00052112, 2: 0.00470935
node 9: 0: 0.23831421, 1: 0.05708918, 2: 0.7045966
node 10: 0: 0.25698753, 1: 0.43917298, 2: 0.30383949
node 11: 0: 0.00782873, 1: 0.9829109*, 2: 0.00926036
node 12: 0: 0.00011981, 1: 0.99969733*, 2: 0.00018286
node 13: 0: 0.00001003, 1: 0.99997752*, 2: 0.00001244
node 14: 0: 0.00006039, 1: 0.99987905*, 2: 0.00006056
node 15: 0: 0.00000943, 1: 0.9999785*, 2: 0.00001207
node 16: 0: 0.0000019, 1: 0.99998834*, 2: 0.00000976
node 17: 0: 0.00086974, 1: 0.98915089*, 2: 0.00997936
node 18: 0: 0.00355788, 1: 0.06116513, 2: 0.93527699*
Eothoracosaurus: 2: 1
node 20: 0: 0.00002718, 1: 0.00015244, 2: 0.99982038*
Thoracosaurus: 2: 1
node 22: 0: 0.00000578, 1: 0.00000775, 2: 0.99998647*
Eosuchus: 2: 1
node 24: 0: 0.00002483, 1: 0.00002936, 2: 0.99994581*
node 25: 0: 0.00005831, 1: 0.00043788, 2: 0.99950381*
Gryposuchus: 2: 1
node 27: 0: 0.00029006, 1: 0.01270007, 2: 0.98700988*
Piscogavialis: 2: 1
Gavialis: 1: 1
Eogavialis: 2: 1
node 31: 0: 0.00058366, 1: 0.99863977*, 2: 0.00077657
node 32: 0: 0.00012513, 1: 0.99973679*, 2: 0.00013808
node 33: 0: 0.00006224, 1: 0.99987492*, 2: 0.00006284
Borealosuchus formidabilis: 1: 1
Borealosuchus threeensis: 1: 1
Borealosuchus sternbergii: 1: 1
node 37: 0: 0.00542504, 1: 0.99388371*, 2: 0.00069125
node 38: 0: 0.99034992*, 1: 0.00900758, 2: 0.0006425
node 39: 0: 0.99991869*, 1: 0.00006391, 2: 0.00001741
Boverisuchus vorax: 0: 1
Boverisuchus magnifrons: 0: 1
node 42: 0: 0.99982223*, 1: 0.00016076, 2: 0.00001701
Planocrania datengensis: 0: 1
Planocrania hengdongensis: 0: 1
node 45: 0: 0.00017345, 1: 0.99979244*, 2: 0.00003411
node 46: 0: 0.00001307, 1: 0.99997732*, 2: 0.00000961
node 47: 0: 0.00001415, 1: 0.99997178*, 2: 0.00001408

node 48: 0: 0.00000163, 1: 0.99999674*, 2: 0.00000163
node 49: 0: 0.00001725, 1: 0.99996549*, 2: 0.00001725
node 50: 0: 0.0000008, 1: 0.9999984*, 2: 0.0000008
node 51: 0: 0.00000264, 1: 0.99999472*, 2: 0.00000264
node 52: 0: 0.00003441, 1: 0.99993119*, 2: 0.00003441
Alligator mississippiensis: 1: 1
Alligator thompsoni: 1: 1
Alligator prenasalis: 1: 1
Allognathosuchus: 1: 1
node 57: 0: 0.00001287, 1: 0.99997427*, 2: 0.00001287
Eocaiman: 1: 1
node 59: 0: 0.00006437, 1: 0.99987126*, 2: 0.00006437
Paleosuchus: 1: 1
node 61: 0: 0.00002112, 1: 0.99995775*, 2: 0.00002112
node 62: 0: 0.0000086, 1: 0.99998279*, 2: 0.0000086
Purussaurus: 1: 1
Mourasuchus: 1: 1
node 65: 0: 0.00000615, 1: 0.99998769*, 2: 0.00000615
Melanosuchus: 1: 1
node 67: 0: 0.00000056, 1: 0.99999888*, 2: 0.00000056
Caiman crocodilis: 1: 1
Caiman yacare: 1: 1
Brachychampsa: 1: 1
Diplocynodon: 1: 1
Leidyosuchus: 1: 1
node 73: 0: 0.00003749, 1: 0.99992605*, 2: 0.00003646
Prodiplocynodon: 1: 1
node 75: 0: 0.00001477, 1: 0.9998031*, 2: 0.00018213
Crocodylus affinis: 1: 1
node 77: 0: 0.00001859, 1: 0.99894251*, 2: 0.0010389
Brachyuranochampsa: 1: 1
node 79: 0: 0.00017475, 1: 0.97842062*, 2: 0.02140463
node 80: 0: 0.00011608, 1: 0.01563082, 2: 0.9842531*
node 81: 0: 0.00001016, 1: 0.0002607, 2: 0.99972914*
node 82: 0: 0.00001795, 1: 0.00002586, 2: 0.9999562*
node 83: 0: 0.00001209, 1: 0.00012691, 2: 0.999861*
node 84: 0: 0.00003736, 1: 0.00078904, 2: 0.9991736*
Tomistoma: 1: 1
Gavialosuchus eggenburgensis: 2: 1
Gavialosuchus americanus: 2: 1
Thecachampsa: 2: 1
Dollosuchoides: 2: 1
Maroccosuchus: 2: 1
node 91: 0: 0.00012985, 1: 0.99954274*, 2: 0.00032741
node 92: 0: 0.00078341, 1: 0.99901733*, 2: 0.00019926
Qunikana: 0: 1
node 94: 0: 0.00003921, 1: 0.9999492*, 2: 0.00001159
Kambara: 1: 1
Australosuchus: 1: 1
node 97: 0: 0.00003697, 1: 0.99991553*, 2: 0.0000475
node 98: 0: 0.00002499, 1: 0.99994958*, 2: 0.00002544

Euthecodon: 1: 1
node 100: 0: 0.00002225, 1: 0.99995549*, 2: 0.00002226
Voay: 1: 1
Osteolaemus: 1: 1
node 103: 0: 0.00001754, 1: 0.99996486*, 2: 0.0000176
Mecistops: 1: 1
node 105: 0: 0.00000128, 1: 0.99999743*, 2: 0.00000128
node 106: 0: 0.00000231, 1: 0.99999538*, 2: 0.00000231
Crocodylus porosus: 1: 1
Crocodylus siamensis: 1: 1
node 109: 0: 0.00000139, 1: 0.99999722*, 2: 0.00000139
node 110: 0: 0.00000069, 1: 0.99999863*, 2: 0.00000069
Crocodylus rhombifer: 1: 1
Crocodylus acutus: 1: 1
Crocodylus niloticus: 1: 1
node 114: 0: 0.00000116, 1: 0.99999723*, 2: 0.00000161
node 115: 0: 0.00021873, 1: 0.99956251*, 2: 0.00021876
Allodaposuchus precedens: 1: 1
node 117: 0: 0.00009546, 1: 0.99980907*, 2: 0.00009547
Lohuecosuchus: 1: 1
Arenysuchus: 1: 1
node 120: 0: 0.00000123, 1: 0.99999751*, 2: 0.00000126
Hylaeochampsa: 1: 1
node 122: 0: 0.00027547, 1: 0.99944905*, 2: 0.00027547
Acynodon adriaticus: 1: 1
Acynodon iberoccitanus: 1: 1
node 125: 0: 0.00016942, 1: 0.99966097*, 2: 0.00016961
Paralligator: 1: 1
Shamosuchus: 1: 1
Bernissartia: 1: 1
node 129: 0: 0.00000339, 1: 0.99999316*, 2: 0.00000345
node 130: 0: 0.00000477, 1: 0.99999046*, 2: 0.00000477
Goniopholis simus: 1: 1
Goniopholis baryglypheaus: 1: 1
Eutretauranosuchus: 1: 1
node 134: 0: 0.00003055, 1: 0.99983529*, 2: 0.00013416
node 135: 0: 0.00027097, 1: 0.99944773*, 2: 0.0002813
Elosuchus: 1: 1
Vectisuchus: 1: 1
node 138: 0: 0.00017648, 1: 0.99844469*, 2: 0.00137883
node 139: 0: 0.00882639, 1: 0.13672896*, 2: 0.85444464*
node 140: 0: 0.00022241, 1: 0.08342218, 2: 0.91635541*
node 141: 0: 0.00009603, 1: 0.98910678*, 2: 0.01079719
Cerrejonisuchus: 1: 1
Anthracosuchus: 1: 1
node 144: 0: 0.00000291, 1: 0.00079629, 2: 0.99920081*
Chenanisuchus: 2: 1
node 146: 0: 0.00000003, 1: 0.00000562, 2: 0.99999435*
Guarinisuchus: 2: 1
node 148: 0: 3.55016564E-9, 1: 0.00000002, 2: 0.99999997*
Rhabdognathus: 1

node 150: 0: 0.00000026, 1: 0.00000026, 2: 0.99999947*
Atlantosuchus: 2: 1
Dyrosaurus: 2: 1
Sokotosuchus: 2: 1
node 154: 0: 0.00000718, 1: 0.99987563*, 2: 0.00011719
Meridiosaurus: 1: 1
node 156: 0: 0.0000281, 1: 0.99877256*, 2: 0.00119935
Sarcosuchus: 1: 1
node 158: 0: 0.00005403, 1: 0.99598001*, 2: 0.00396596
node 159: 0: 0.00004707, 1: 0.9962191*, 2: 0.00373384
Pholidosaurus: 1: 1
Oceanosuchus: 2: 1
node 162: 0: 0.00373967, 1: 0.05904311, 2: 0.93721722*
Terminonaris browni: 2: 1
Terminonaris robusta: 2: 1
node 165: 0: 0.00045146, 1: 0.99902707*, 2: 0.00052147
Theriosuchus pusilis: 1: 1
Theriosuchus guimarotae: 1: 1
node 168: 0: 0.89316809*, 1: 0.10088956, 2: 0.00594235
node 169: 0: 0.84781974*, 1: 0.14833361*, 2: 0.00384665
node 170: 0: 0.03773979, 1: 0.96106295*, 2: 0.00119726
node 171: 0: 0.00127179, 1: 0.99857108*, 2: 0.00015713
Mahajangasuchus: 1: 1
Kaprosuchus: 1: 1
Stolokrosuchus: 1: 1
node 175: 0: 0.98784978*, 1: 0.01109731, 2: 0.00105291
Lomasuchus: 0: 1
node 177: 0: 0.99980566*, 1: 0.0001324, 2: 0.00006194
Uberabasuchus: 0: 1
Peirosaurus: 0: 1
node 180: 0: 0.99550637*, 1: 0.00419611, 2: 0.00029752
node 181: 0: 0.99950544*, 1: 0.00036911, 2: 0.00012546
Araripesuchus gomesii: 0: 1
Araripesuchus patagonicus: 0: 1
node 184: 0: 0.99962376*, 1: 0.00033907, 2: 0.00003717
Urugaysuchus: 0: 1
node 186: 0: 0.99994247*, 1: 0.00004205, 2: 0.00001548
Libycosuchus: 0: 1
node 188: 0: 0.99995741*, 1: 0.00002296, 2: 0.00001963
Simosuchus: 0: 1
node 190: 0: 0.99999578*, 1: 0.00000213, 2: 0.00000209
Malawisuchus: 0: 1
node 192: 0: 0.99999568*, 1: 0.00000216, 2: 0.00000216
node 193: 0: 0.99984077*, 1: 0.00007962, 2: 0.00007962
Notosuchus: 0: 1
Mariliasuchus: 0: 1
node 196: 0: 0.99999881*, 1: 0.00000059, 2: 0.00000059
node 197: 0: 0.99999769*, 1: 0.00000115, 2: 0.00000115
Sphagesaurus: 0: 1
Chimaerasuchus: 0: 1
node 200: 0: 0.99990206*, 1: 0.00004897, 2: 0.00004897

Comahuesuchus: 0: 1
node 202: 0: 0.99999198*, 1: 0.00000401, 2: 0.00000401
Pissarrachampsa: 0: 1
Stratiotosuchus: 0: 1
node 205: 0: 0.0015466, 1: 0.00037314, 2: 0.99808026*
node 206: 0: 0.00001762, 1: 0.0000047, 2: 0.99997768*
Pelagosaurus: 2: 1
node 208: 0: 0.0000017, 1: 0.00000155, 2: 0.99999674*
Teleidosaurus calvadosii: 2: 1
node 210: 0: 0.00000067, 1: 0.00000067, 2: 0.99999866*
Eoneustes bathonicus: 2: 1
node 212: 0: 0.00000052, 1: 0.00000052, 2: 0.99999895*
Eoneustes gaudryi: 2: 1
node 214: 0: 0.0000002, 1: 0.0000002, 2: 0.99999959*
Zoneait: 2: 1
node 216: 0: 0.00000102, 1: 0.00000102, 2: 0.99999795*
Metriorhynchus superciliosus: 2: 1
node 218: 0: 0.00000022, 1: 0.00000022, 2: 0.99999956*
node 219: 0: 0.00000319, 1: 0.00000319, 2: 0.99999362*
Metriorhynchus leedsi: 2: 1
Cricosaurus suevicus: 2: 1
node 222: 0: 0.00000033, 1: 0.00000033, 2: 0.99999935*
node 223: 0: 0.00000007, 1: 0.00000007, 2: 0.99999986*
Metriorhynchus durobrivensis: 2: 1
Metriorhynchus casamiquelai: 2: 1
node 226: 0: 0.00000247, 1: 0.00000247, 2: 0.99999506*
node 227: 0: 0.0000014, 1: 0.0000014, 2: 0.9999972*
Geosaurus grandis: 2: 1
Geosaurus giganteus: 2: 1
node 230: 0: 0.00000292, 1: 0.00000292, 2: 0.99999417*
Dakosaurus maximus: 2: 1
Dakosaurus andiniensis: 2: 1
node 233: 0: 0.00002938, 1: 0.00000719, 2: 0.99996343*
Steneosaurus gracilirostris: 2: 1
node 235: 0: 0.00000198, 1: 0.00000065, 2: 0.99999737*
Teleosaurus: 2: 1
node 237: 0: 0.00000009, 1: 0.00000011, 2: 0.99999979*
node 238: 0: 0.00000004, 1: 0.00000213, 2: 0.99999783*
node 239: 0: 0.00000328, 1: 0.00038289, 2: 0.99961383*
node 240: 0: 0.000326, 1: 0.96008963*, 2: 0.03958436
Peipehsuchus: 1: 1
Thai teleosaurid: 1: 1
node 243: 0: 0.00000001, 1: 0.0000007, 2: 0.99999929*
Platysuchus multiscrobiculatus: 2: 1
Steneosaurus bollensis: 2: 1
Steneosaurus brevior: 2: 1
node 247: 0: 0.00001056, 1: 0.00001056, 2: 0.99997887*
node 248: 0: 0.000012, 1: 0.000012, 2: 0.99997601*
Steneosaurus durobrivensis: 2: 1
Machimosaurus: 2: 1
Steneosaurus leedsi: 2: 1

node 252: 0: 0.99976955*, 1: 0.0000317, 2: 0.00019875
Calsoyasuchus: 0: 1
node 254: 0: 0.99986929*, 1: 0.00006249, 2: 0.00006821
Hsisosuchus dashanpuensis: 0: 1
Hsisosuchus chungkingensis: 0: 1
node 257: 0: 0.99999238*, 1: 0.00000331, 2: 0.00000431
node 258: 0: 0.99999767*, 1: 0.00000114, 2: 0.00000119
node 259: 0: 0.99999218*, 1: 0.00000391, 2: 0.00000392
node 260: 0: 0.99999952*, 1: 0.00000024, 2: 0.00000024
Protosuchus: 0: 1
Orthosuchus: 0: 1
Edentosuchus: 0: 1
node 264: 0: 0.99951162*, 1: 0.00024419, 2: 0.00024419
node 265: 0: 0.99975792*, 1: 0.00012104, 2: 0.00012104
Sichuanosuchus: 0: 1
Shantungosuchus: 0: 1
node 268: 0: 0.99917433*, 1: 0.00041284, 2: 0.00041284
Zosuchus: 0: 1
Fruitachampsa: 0: 1
node 271: 0: 0.99294753*, 1: 0.00352618, 2: 0.00352629
Zaraasuchus: 0: 1
Gobiosuchus: 0: 1
Kayentasuchus: 0: 1
node 275: 0: 0.9995803*, 1: 0.00020985, 2: 0.00020985
Junggarsuchus: 0: 1
Almadasuchus: 0: 1
node 278: 0: 0.9999773*, 1: 0.00001135, 2: 0.00001135
node 279: 0: 0.99995828*, 1: 0.00002086, 2: 0.00002086
Dibothrosuchus: 0: 1
Sphenosuchus: 0: 1
Hesperosuchus: 0: 1
Postosuchus: 0: 1
Gracilisuchus: 0: 1

d) Thalattosuchia as sister to Tethysuchia ("longirostrine clade"):

Reference tree:

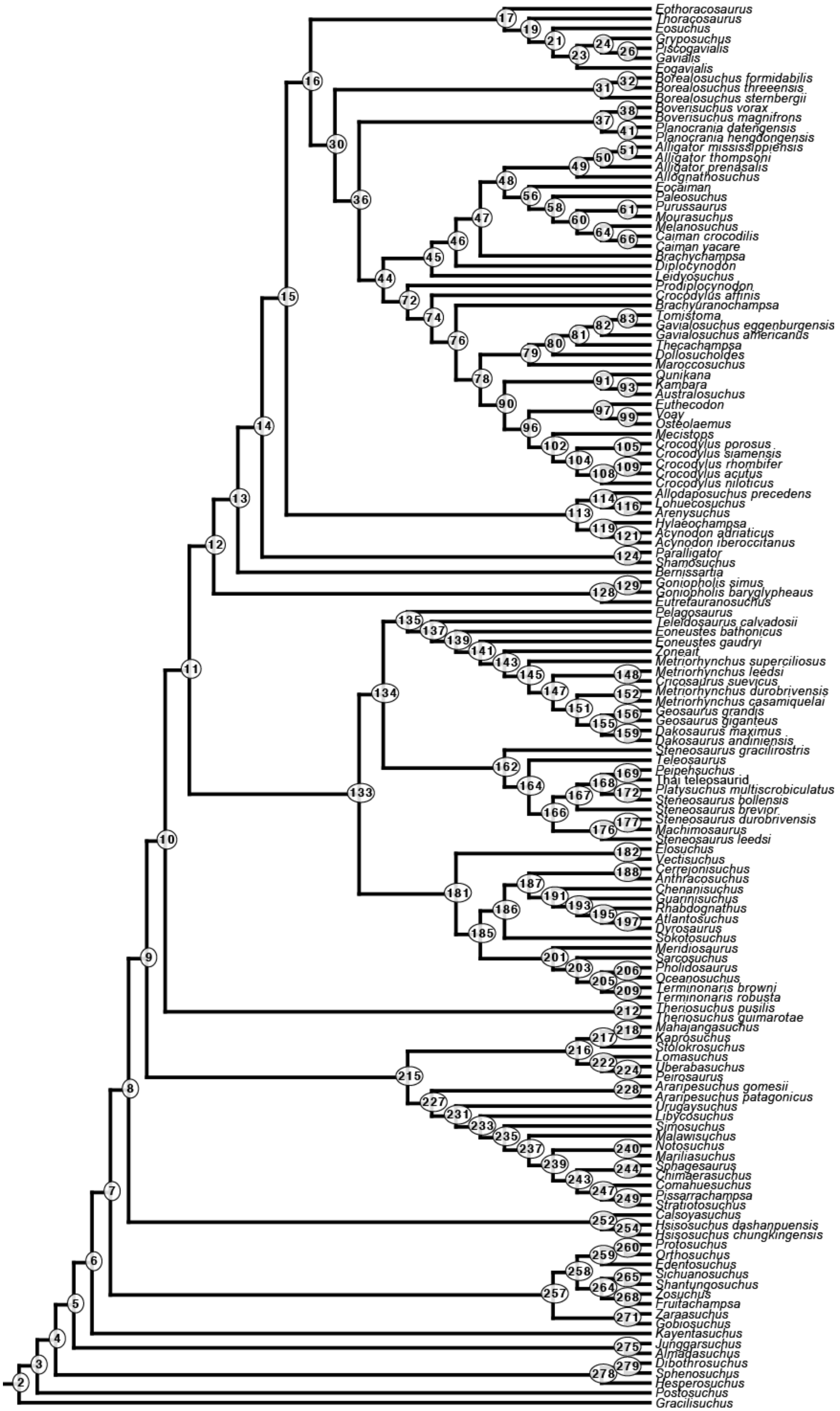

Character: habitat
Marginal prob. recon. with model Mk1 (est.) [rate 0.00208025 [est.]] -log L.:69.537371 (Opt.: width 0.0) Reporting likelihoods as Proportional Likelihoods; Threshold when decisions made: 2
Calc. by Maximum likelihood reconstruct (Generic categorical) (id# 1936)

node 2: 0: 0.99965543*, 1: 0.00017229, 2: 0.00017229
node 3: 0: 0.99995908*, 1: 0.00002046, 2: 0.00002046
node 4: 0: 0.99998028*, 1: 0.00000986, 2: 0.00000986
node 5: 0: 0.99998429*, 1: 0.0000079, 2: 0.00000781
node 6: 0: 0.99999782*, 1: 0.00000122, 2: 0.00000096
node 7: 0: 0.9999948*, 1: 0.00000475, 2: 0.00000045
node 8: 0: 0.99663016*, 1: 0.00325159, 2: 0.00011826
node 9: 0: 0.47760434*, 1: 0.51791479*, 2: 0.00448087
node 10: 0: 0.03783508, 1: 0.95055993*, 2: 0.01160498
node 11: 0: 0.01329612, 1: 0.96721971*, 2: 0.01948417
node 12: 0: 0.0006769, 1: 0.99835214*, 2: 0.00097095
node 13: 0: 0.00014448, 1: 0.99969738*, 2: 0.00015814
node 14: 0: 0.00000989, 1: 0.99997736*, 2: 0.00001275
node 15: 0: 0.00000162, 1: 0.99998941*, 2: 0.00000897
node 16: 0: 0.00074695, 1: 0.98998994*, 2: 0.00926311
node 17: 0: 0.00307256, 1: 0.05703033, 2: 0.93989711*
Eothoracosaurus: 2: 1
node 19: 0: 0.00002181, 1: 0.00013135, 2: 0.99984684*
Thoracosaurus: 2: 1
node 21: 0: 0.00000458, 1: 0.00000619, 2: 0.99998923*
Eosuchus: 2: 1
node 23: 0: 0.00001978, 1: 0.00002343, 2: 0.99995679*
node 24: 0: 0.0000466, 1: 0.00037492, 2: 0.99957848*
Gryposuchus: 2: 1
node 26: 0: 0.00024957, 1: 0.01177262, 2: 0.98797782*
Piscogavialis: 2: 1
Gavialis: 1: 1
Eogavialis: 2: 1
node 30: 0: 0.00049841, 1: 0.99883442*, 2: 0.00066717
node 31: 0: 0.00009934, 1: 0.99979077*, 2: 0.00010989
node 32: 0: 0.00004932, 1: 0.9999009*, 2: 0.00004978
Borealosuchus formidabilis: 1: 1
Borealosuchus threeensis: 1: 1
Borealosuchus sternbergii: 1: 1
node 36: 0: 0.0049933, 1: 0.99441292*, 2: 0.00059378
node 37: 0: 0.99108281*, 1: 0.00836452, 2: 0.00055267
node 38: 0: 0.99993164*, 1: 0.00005443, 2: 0.00001393
Boverisuchus vorax: 0: 1
Boverisuchus magnifrons: 0: 1
node 41: 0: 0.99984787*, 1: 0.00013852, 2: 0.00001361
Planocrania datengensis: 0: 1
Planocrania hengdongensis: 0: 1
node 44: 0: 0.00014719, 1: 0.9998257*, 2: 0.00002711
node 45: 0: 0.00001039, 1: 0.999982*, 2: 0.00000761
node 46: 0: 0.00001126, 1: 0.99997753*, 2: 0.00001121
node 47: 0: 0.00000128, 1: 0.99999744*, 2: 0.00000128

node 48: 0: 0.00001375, 1: 0.9999725*, 2: 0.00001375
node 49: 0: 0.00000064, 1: 0.99999872*, 2: 0.00000064
node 50: 0: 0.00000211, 1: 0.99999579*, 2: 0.00000211
node 51: 0: 0.00002748, 1: 0.99994505*, 2: 0.00002748
Alligator mississippiensis: 1: 1
Alligator thompsoni: 1: 1
Alligator prenasalis: 1: 1
Allognathosuchus: 1: 1
node 56: 0: 0.00001021, 1: 0.99997958*, 2: 0.00001021
Eocaiman: 1: 1
node 58: 0: 0.00005117, 1: 0.99989767*, 2: 0.00005117
Paleosuchus: 1: 1
node 60: 0: 0.00001667, 1: 0.99996666*, 2: 0.00001667
node 61: 0: 0.00000685, 1: 0.9999863*, 2: 0.00000685
Purussaurus: 1: 1
Mourasuchus: 1: 1
node 64: 0: 0.00000491, 1: 0.99999017*, 2: 0.00000491
Melanosuchus: 1: 1
node 66: 0: 0.00000044, 1: 0.99999911*, 2: 0.00000044
Caiman crocodilis: 1: 1
Caiman yacare: 1: 1
Brachychampsa: 1: 1
Diplocynodon: 1: 1
Leidyosuchus: 1: 1
node 72: 0: 0.00002985, 1: 0.99994136*, 2: 0.00002879
Prodiplocynodon: 1: 1
node 74: 0: 0.00001153, 1: 0.99984318*, 2: 0.00014529
Crocodylus affinis: 1: 1
node 76: 0: 0.0000147, 1: 0.99910077*, 2: 0.00088453
Brachyuranochampsa: 1: 1
node 78: 0: 0.00015009, 1: 0.98003371*, 2: 0.01981619
node 79: 0: 0.00010005, 1: 0.01453462, 2: 0.98536533*
node 80: 0: 0.00000813, 1: 0.00022466, 2: 0.99976721*
node 81: 0: 0.00001431, 1: 0.00002067, 2: 0.99996502*
node 82: 0: 0.00000965, 1: 0.00010882, 2: 0.99988152*
node 83: 0: 0.00003185, 1: 0.00072525, 2: 0.9992429*
Tomistoma: 1: 1
Gavialosuchus eggenburgensis: 2: 1
Gavialosuchus americanus: 2: 1
Thecachampsa: 2: 1
Dollosuchoides: 2: 1
Maroccosuchus: 2: 1
node 90: 0: 0.00011073, 1: 0.99960949*, 2: 0.00027978
node 91: 0: 0.0007164, 1: 0.99911336*, 2: 0.00017024
Qunikana: 0: 1
node 93: 0: 0.00003327, 1: 0.9999575*, 2: 0.00000923
Kambara: 1: 1
Australosuchus: 1: 1
node 96: 0: 0.00002938, 1: 0.99993288*, 2: 0.00003774
node 97: 0: 0.00001985, 1: 0.99995997*, 2: 0.00002018
Euthecodon: 1: 1

node 99: 0: 0.00001777, 1: 0.99996446*, 2: 0.00001777
Voay: 1: 1
Osteolaemus: 1: 1
node 102: 0: 0.00001399, 1: 0.99997198*, 2: 0.00001403
Mecistops: 1: 1
node 104: 0: 0.00000101, 1: 0.99999798*, 2: 0.00000101
node 105: 0: 0.00000185, 1: 0.99999631*, 2: 0.00000185
Crocodylus porosus: 1: 1
Crocodylus siamensis: 1: 1
node 108: 0: 0.00000111, 1: 0.99999778*, 2: 0.00000111
node 109: 0: 0.00000055, 1: 0.9999989*, 2: 0.00000055
Crocodylus rhombifer: 1: 1
Crocodylus acutus: 1: 1
Crocodylus niloticus: 1: 1
node 113: 0: 0.00000092, 1: 0.99999778*, 2: 0.00000131
node 114: 0: 0.00017381, 1: 0.99965235*, 2: 0.00017384
Allodaposuchus precedens: 1: 1
node 116: 0: 0.00007562, 1: 0.99984875*, 2: 0.00007562
Lohuecosuchus: 1: 1
Arenysuchus: 1: 1
node 119: 0: 0.00000098, 1: 0.99999802*, 2: 0.000001
Hylaeochampsa: 1: 1
node 121: 0: 0.00021956, 1: 0.99956088*, 2: 0.00021956
Acynodon adriaticus: 1: 1
Acynodon iberoccitanus: 1: 1
node 124: 0: 0.00013525, 1: 0.99972931*, 2: 0.00013545
Paralligator: 1: 1
Shamosuchus: 1: 1
Bernissartia: 1: 1
node 128: 0: 0.00003819, 1: 0.99991301*, 2: 0.00004881
node 129: 0: 0.00001491, 1: 0.99996978*, 2: 0.00001531
Goniopholis simus: 1: 1
Goniopholis baryglypheaus: 1: 1
Eutretauranosuchus: 1: 1
node 133: 0: 0.00560567, 1: 0.94009442*, 2: 0.05429991
node 134: 0: 0.00003051, 1: 0.00422577, 2: 0.99574372*
node 135: 0: 0.00000061, 1: 0.00003754, 2: 0.99996185*
Pelagosaurus: 2: 1
node 137: 0: 0.00000103, 1: 0.00000151, 2: 0.99999746*
Teleidosaurus calvadosii: 2: 1
node 139: 0: 0.00000053, 1: 0.00000054, 2: 0.99999894*
Eoneustes bathonicus: 2: 1
node 141: 0: 0.00000042, 1: 0.00000042, 2: 0.99999917*
Eoneustes gaudryi: 2: 1
node 143: 0: 0.00000016, 1: 0.00000016, 2: 0.99999968*
Zoneait: 2: 1
node 145: 0: 0.00000082, 1: 0.00000082, 2: 0.99999836*
Metriorhynchus superciliosus: 2: 1
node 147: 0: 0.00000017, 1: 0.00000017, 2: 0.99999965*
node 148: 0: 0.00000255, 1: 0.00000255, 2: 0.9999949*
Metriorhynchus leedsi: 2: 1

Cricosaurus suevicus: 2: 1
node 151: 0: 0.00000026, 1: 0.00000026, 2: 0.99999948*
node 152: 0: 0.00000005, 1: 0.00000005, 2: 0.99999989*
Metriorhynchus durobrivensis: 2: 1
Metriorhynchus casamiquelai: 2: 1
node 155: 0: 0.00000197, 1: 0.00000197, 2: 0.99999605*
node 156: 0: 0.00000112, 1: 0.00000112, 2: 0.99999776*
Geosaurus grandis: 2: 1
Geosaurus giganteus: 2: 1
node 159: 0: 0.00000233, 1: 0.00000233, 2: 0.99999534*
Dakosaurus maximus: 2: 1
Dakosaurus andiniensis: 2: 1
node 162: 0: 0.00000056, 1: 0.00006296, 2: 0.99993648*
Steneosaurus gracilirostris: 2: 1
node 164: 0: 0.00000012, 1: 0.00000351, 2: 0.99999637*
Teleosaurus: 2: 1
node 166: 0: 0.00000003, 1: 0.00000012, 2: 0.99999985*
node 167: 0: 0.00000001, 1: 0.00000089, 2: 0.9999991*
node 168: 0: 0.00000159, 1: 0.00022402, 2: 0.99977439*
node 169: 0: 0.00028021, 1: 0.95852866*, 2: 0.04119114
Peipehsuchus: 1: 1
Thai teleosaurid: 1: 1
node 172: 0: 4.33861975E-9, 1: 0.00000028, 2: 0.99999971*
Platysuchus multiscrobiculatus: 2: 1
Steneosaurus bollensis: 2: 1
Steneosaurus brevior: 2: 1
node 176: 0: 0.00000755, 1: 0.00000755, 2: 0.9999849*
node 177: 0: 0.00000875, 1: 0.00000875, 2: 0.9999825*
Steneosaurus durobrivensis: 2: 1
Machimosaurus: 2: 1
Steneosaurus leedsi: 2: 1
node 181: 0: 0.00078252, 1: 0.98049466*, 2: 0.01872282
node 182: 0: 0.00055673, 1: 0.99707433*, 2: 0.00236895
Elosuchus: 1: 1
Vectisuchus: 1: 1
node 185: 0: 0.00075687, 1: 0.9801032*, 2: 0.01913993
node 186: 0: 0.00766518, 1: 0.11690735, 2: 0.87542747*
node 187: 0: 0.00018768, 1: 0.0744442, 2: 0.92536812*
node 188: 0: 0.00008345, 1: 0.98978392*, 2: 0.01013263
Cerrejonisuchus: 1: 1
Anthracosuchus: 1: 1
node 191: 0: 0.00000227, 1: 0.00065997, 2: 0.99933777*
Chenanisuchus: 2: 1
node 193: 0: 0.00000002, 1: 0.00000433, 2: 0.99999565*
Guarinisuchus: 2: 1
node 195: 0: 2.82450088E-9, 1: 0.00000002, 2: 0.99999998*
Rhabdognathus: 2: 1
node 197: 0: 0.00000021, 1: 0.00000021, 2: 0.99999958*
Atlantosuchus: 2: 1
Dyrosaurus: 2: 1
Sokotosuchus: 2: 1

node 201: 0: 0.00003777, 1: 0.99839945*, 2: 0.00156279
Meridiosaurus: 1: 1
node 203: 0: 0.00003477, 1: 0.99733703*, 2: 0.0026282
Sarcosuchus: 1: 1
node 205: 0: 0.0000522, 1: 0.99455837*, 2: 0.00538943
node 206: 0: 0.00004387, 1: 0.99491814*, 2: 0.005038
Pholidosaurus: 1: 1
Oceanosuchus: 2: 1
node 209: 0: 0.00322625, 1: 0.05493175, 2: 0.94184201*
Terminonaris browni: 2: 1
Terminonaris robusta: 2: 1
node 212: 0: 0.00223896, 1: 0.99694693*, 2: 0.00081411
Theriosuchus pusilis: 1: 1
Theriosuchus guimarotae: 1: 1
node 215: 0: 0.67786529*, 1: 0.32053146*, 2: 0.00160325
node 216: 0: 0.63860555*, 1: 0.35767522*, 2: 0.00371923
node 217: 0: 0.03921556, 1: 0.95865777*, 2: 0.00212666
node 218: 0: 0.0008486, 1: 0.99898175*, 2: 0.00016965
Mahajangasuchus: 1: 1
Kaprosuchus: 1: 1
Stolokrosuchus: 1: 1
node 222: 0: 0.96547192*, 1: 0.03163737, 2: 0.00289071
Lomasuchus: 0: 1
node 224: 0: 0.99969862*, 1: 0.00022094, 2: 0.00008043
Uberabasuchus: 0: 1
Peirosaurus: 0: 1
node 227: 0: 0.97624522*, 1: 0.02312949, 2: 0.00062529
node 228: 0: 0.99715945*, 1: 0.00231284, 2: 0.00052771
Araripesuchus gomesii: 0: 1
Araripesuchus patagonicus: 0: 1
node 231: 0: 0.99703495*, 1: 0.00277664, 2: 0.00018841
Urugaysuchus: 0: 1
node 233: 0: 0.99953119*, 1: 0.00038178, 2: 0.00008703
Libycosuchus: 0: 1
node 235: 0: 0.99979241*, 1: 0.00012364, 2: 0.00008395
Simosuchus: 0: 1
node 237: 0: 0.99997311*, 1: 0.00001391, 2: 0.00001298
Malawisuchus: 0: 1
node 239: 0: 0.99999376*, 1: 0.00000312, 2: 0.00000312
node 240: 0: 0.99987276*, 1: 0.00006362, 2: 0.00006362
Notosuchus: 0: 1
Mariliasuchus: 0: 1
node 243: 0: 0.99999897*, 1: 0.00000052, 2: 0.00000052
node 244: 0: 0.99999815*, 1: 0.00000092, 2: 0.00000092
Sphagesaurus: 0: 1
Chimaerasuchus: 0: 1
node 247: 0: 0.99992178*, 1: 0.00003911, 2: 0.00003911
Comahuesuchus: 0: 1
node 249: 0: 0.9999936*, 1: 0.0000032, 2: 0.0000032
Pissarrachampsa: 0: 1
Stratiotosuchus: 0: 1

node 252: 0: 0.99985912*, 1: 0.00012849, 2: 0.00001239
Calsoyasuchus: 0: 1
node 254: 0: 0.99989761*, 1: 0.00005305, 2: 0.00004934
Hsisosuchus dashanpuensis: 0: 1
Hsisosuchus chungkingensis: 0: 1
node 257: 0: 0.99999434*, 1: 0.00000315, 2: 0.00000252
node 258: 0: 0.99999823*, 1: 0.0000009, 2: 0.00000087
node 259: 0: 0.9999938*, 1: 0.00000311, 2: 0.0000031
node 260: 0: 0.99999962*, 1: 0.00000019, 2: 0.00000019
Protosuchus: 0: 1
Orthosuchus: 0: 1
Edentosuchus: 0: 1
node 264: 0: 0.99961641*, 1: 0.0001918, 2: 0.00019179
node 265: 0: 0.99980722*, 1: 0.00009639, 2: 0.00009639
Sichuanosuchus: 0: 1
Shantungosuchus: 0: 1
node 268: 0: 0.99934747*, 1: 0.00032627, 2: 0.00032627
Zosuchus: 0: 1
Fruitachampsa: 0: 1
node 271: 0: 0.99439297*, 1: 0.00280355, 2: 0.00280348
Zaraasuchus: 0: 1
Gobiosuchus: 0: 1
Kayentasuchus: 0: 1
node 275: 0: 0.99966544*, 1: 0.00016728, 2: 0.00016728
Junggarsuchus: 0: 1
Almadasuchus: 0: 1
node 278: 0: 0.99998193*, 1: 0.00000904, 2: 0.00000904
node 279: 0: 0.99996668*, 1: 0.00001666, 2: 0.00001666
Dibothrosuchus: 0: 1
Sphenosuchus: 0: 1
Hesperosuchus: 0: 1
Postosuchus: 0: 1
Gracilisuchus: 0: 1

e) Gavialis and Tomistoma as living sister taxa (including thoracosaurs):

Reference tree:

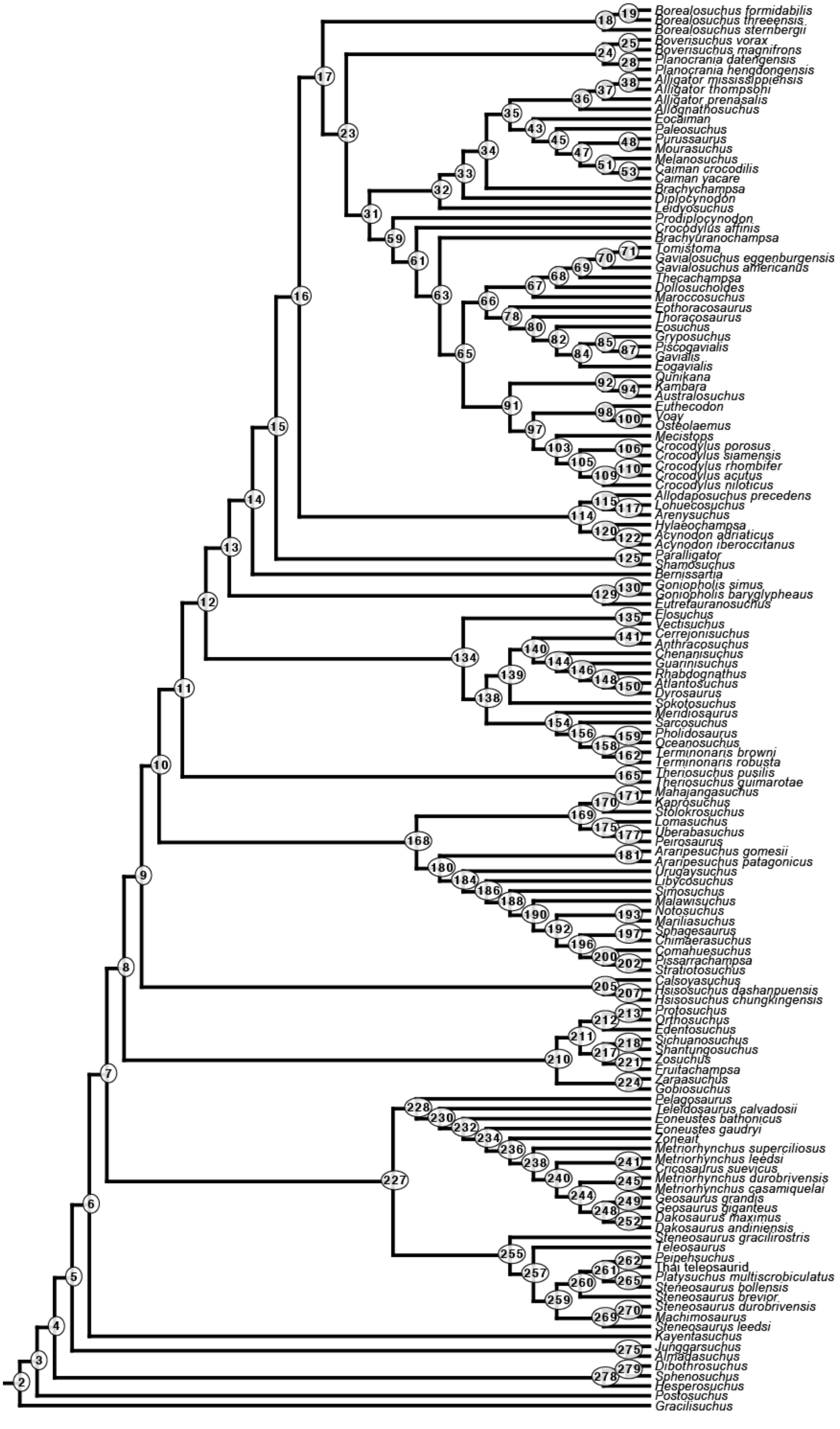

Character: habitat
Marginal prob. recon. with model Mk1 (est.) [rate 0.00191965 [est.]] -log L.:62.07528795 (Opt.: width 0.0) Reporting likelihoods as Proportional Likelihoods; Threshold when decisions made: 2
Calc. by Maximum likelihood reconstruct (Generic categorical) (id# 1176)

node 2: 0: 0.99970801*, 1: 0.000146, 2: 0.000146
node 3: 0: 0.99996805*, 1: 0.00001597, 2: 0.00001598
node 4: 0: 0.99998481*, 1: 0.00000756, 2: 0.00000763
node 5: 0: 0.9999798*, 1: 0.00000563, 2: 0.00001457
node 6: 0: 0.99997161*, 1: 0.00000133, 2: 0.00002706
node 7: 0: 0.99953456*, 1: 0.00001125, 2: 0.0004542
node 8: 0: 0.99998961*, 1: 0.0000022, 2: 0.00000819
node 9: 0: 0.99907478*, 1: 0.00088395, 2: 0.00004127
node 10: 0: 0.77469687*, 1: 0.22341807*, 2: 0.00188506
node 11: 0: 0.02026515, 1: 0.97948009*, 2: 0.00025476
node 12: 0: 0.0002592, 1: 0.99973145*, 2: 0.00000935
node 13: 0: 0.00001214, 1: 0.99998397*, 2: 0.00000389
node 14: 0: 0.00003904, 1: 0.9999222*, 2: 0.00003876
node 15: 0: 0.00000569, 1: 0.99998868*, 2: 0.00000563
node 16: 0: 0.00000076, 1: 0.99999864*, 2: 0.0000006
node 17: 0: 0.00030802, 1: 0.99964669*, 2: 0.00004528
node 18: 0: 0.00009898, 1: 0.99981925*, 2: 0.00008177
node 19: 0: 0.00005548, 1: 0.9998898*, 2: 0.00005472
Borealosuchus formidabilis: 1: 1
Borealosuchus threeensis: 1: 1
Borealosuchus sternbergii: 1: 1
node 23: 0: 0.00547099, 1: 0.99414254*, 2: 0.00038647
node 24: 0: 0.99289589*, 1: 0.00663609, 2: 0.00046802
node 25: 0: 0.99994892*, 1: 0.00004035, 2: 0.00001072
Boverisuchus vorax: 0: 1
Boverisuchus magnifrons: 0: 1
node 28: 0: 0.9998879*, 1: 0.00010164, 2: 0.00001046
Planocrania datengensis: 0: 1
Planocrania hengdongensis: 0: 1
node 31: 0: 0.00023367, 1: 0.99973183*, 2: 0.0000345
node 32: 0: 0.00001473, 1: 0.99997537*, 2: 0.0000099
node 33: 0: 0.00001014, 1: 0.9999798*, 2: 0.00001007
node 34: 0: 0.00000102, 1: 0.99999797*, 2: 0.00000101
node 35: 0: 0.00001074, 1: 0.99997852*, 2: 0.00001074
node 36: 0: 0.0000005, 1: 0.999999*, 2: 0.0000005
node 37: 0: 0.00000165, 1: 0.99999671*, 2: 0.00000165
node 38: 0: 0.00002151, 1: 0.99995698*, 2: 0.00002151
Alligator mississippiensis: 1: 1
Alligator thompsoni: 1: 1
Alligator prenasalis: 1: 1
Allognathosuchus: 1: 1
node 43: 0: 0.00000795, 1: 0.99998411*, 2: 0.00000795
Eocaiman: 1: 1
node 45: 0: 0.00003987, 1: 0.99992026*, 2: 0.00003987
Paleosuchus: 1: 1
node 47: 0: 0.0000129, 1: 0.9999742*, 2: 0.0000129

node 48: 0: 0.00000535, 1: 0.9999893*, 2: 0.00000535
Purussaurus: 1: 1
Mourasuchus: 1: 1
node 51: 0: 0.00000385, 1: 0.9999923*, 2: 0.00000385
Melanosuchus: 1: 1
node 53: 0: 0.00000035, 1: 0.99999931*, 2: 0.00000035
Caiman crocodilis: 1: 1
Caiman yacare: 1: 1
Brachychampsa: 1: 1
Diplocynodon: 1: 1
Leidyosuchus: 1: 1
node 59: 0: 0.00000219, 1: 0.99997935*, 2: 0.00001847
Prodiplocynodon: 1: 1
node 61: 0: 0.0000012, 1: 0.99991522*, 2: 0.00008358
Crocodylus affinis: 1: 1
node 63: 0: 0.0000083, 1: 0.99864867*, 2: 0.00134303
Brachyuranochampsa: 1: 1
node 65: 0: 0.00007314, 1: 0.97758331*, 2: 0.02234354
node 66: 0: 0.00005464, 1: 0.02051281, 2: 0.97943254*
node 67: 0: 0.00001569, 1: 0.00035882, 2: 0.99962549*
node 68: 0: 0.00000469, 1: 0.0000075, 2: 0.99998782*
node 69: 0: 0.00001108, 1: 0.00001277, 2: 0.99997615*
node 70: 0: 0.00000755, 1: 0.00009159, 2: 0.99990085*
node 71: 0: 0.0000268, 1: 0.00066168, 2: 0.99931151*
Tomistoma: 1: 1
Gavialosuchus eggenburgensis: 2: 1
Gavialosuchus americanus: 2: 1
Thecachampsa: 2: 1
Dollosuchoides: 2: 1
Maroccosuchus: 2: 1
node 78: 0: 0.00000112, 1: 0.00020011, 2: 0.99979877*
Eothoracosaurus: 2: 1
node 80: 0: 0.00000113, 1: 0.00000339, 2: 0.99999548*
Thoracosaurus: 2: 1
node 82: 0: 0.00000333, 1: 0.00000337, 2: 0.9999933*
Eosuchus: 2: 1
node 84: 0: 0.00001544, 1: 0.00001829, 2: 0.99996627*
node 85: 0: 0.00003651, 1: 0.00031678, 2: 0.99964672*
Gryposuchus: 2: 1
node 87: 0: 0.00021191, 1: 0.01084105, 2: 0.98894704*
Piscogavialis: 2: 1
Gavialis: 1: 1
Eogavialis: 2: 1
node 91: 0: 0.0002753, 1: 0.99883694*, 2: 0.00088776
node 92: 0: 0.00293786, 1: 0.99646823*, 2: 0.00059391
Qunikana: 0: 1
node 94: 0: 0.00015084, 1: 0.99980999*, 2: 0.00003917
Kambara: 1: 1
Australosuchus: 1: 1
node 97: 0: 0.00006786, 1: 0.99982728*, 2: 0.00010487
node 98: 0: 0.00003808, 1: 0.99992223*, 2: 0.00003969

Euthecodon: 1: 1
node 100: 0: 0.00001693, 1: 0.99996613*, 2: 0.00001694
Voay: 1: 1
Osteolaemus: 1: 1
node 103: 0: 0.00001483, 1: 0.99997021*, 2: 0.00001496
Mecistops: 1: 1
node 105: 0: 0.00000083, 1: 0.99999835*, 2: 0.00000083
node 106: 0: 0.00000145, 1: 0.9999971*, 2: 0.00000145
Crocodylus porosus: 1: 1
Crocodylus siamensis: 1: 1
node 109: 0: 0.00000087, 1: 0.99999826*, 2: 0.00000087
node 110: 0: 0.00000043, 1: 0.99999914*, 2: 0.00000043
Crocodylus rhombifer: 1: 1
Crocodylus acutus: 1: 1
Crocodylus niloticus: 1: 1
node 114: 0: 0.00000068, 1: 0.99999865*, 2: 0.00000067
node 115: 0: 0.00013539, 1: 0.99972922*, 2: 0.00013539
Allodaposuchus precedens: 1: 1
node 117: 0: 0.00005872, 1: 0.99988255*, 2: 0.00005872
Lohuecosuchus: 1: 1
Arenysuchus: 1: 1
node 120: 0: 0.00000076, 1: 0.99999849*, 2: 0.00000076
Hylaeochampsa: 1: 1
node 122: 0: 0.00017155, 1: 0.99965691*, 2: 0.00017155
Acynodon adriaticus: 1: 1
Acynodon iberoccitanus: 1: 1
node 125: 0: 0.00010554, 1: 0.99978892*, 2: 0.00010554
Paralligator: 1: 1
Shamosuchus: 1: 1
Bernissartia: 1: 1
node 129: 0: 0.00000236, 1: 0.99999545*, 2: 0.00000219
node 130: 0: 0.00000315, 1: 0.99999371*, 2: 0.00000314
Goniopholis simus: 1: 1
Goniopholis baryglypheaus: 1: 1
Eutretauranosuchus: 1: 1
node 134: 0: 0.000034, 1: 0.99990466*, 2: 0.00006134
node 135: 0: 0.00017307, 1: 0.99965148*, 2: 0.00017545
Elosuchus: 1: 1
Vectisuchus: 1: 1
node 138: 0: 0.00013882, 1: 0.99867201*, 2: 0.00118917
node 139: 0: 0.00650869, 1: 0.12875616*, 2: 0.86473515*
node 140: 0: 0.00016442, 1: 0.08276393, 2: 0.91707165*
node 141: 0: 0.00007036, 1: 0.99065894*, 2: 0.0092707
Cerrejonisuchus: 1: 1
Anthracosuchus: 1: 1
node 144: 0: 0.00000191, 1: 0.00067697, 2: 0.99932111*
Chenanisuchus: 2: 1
node 146: 0: 0.00000002, 1: 0.00000409, 2: 0.99999589*
Guarinisuchus: 2: 1
node 148: 0: 2.21192637E-9, 1: 0.00000001, 2: 0.99999998*
Rhabdognathus: 2: 1

node 150: 0: 0.00000016, 1: 0.00000017, 2: 0.99999967*
Atlantosuchus: 2: 1
Dyrosaurus: 2: 1
Sokotosuchus: 2: 1
node 154: 0: 0.00000478, 1: 0.99989839*, 2: 0.00009683
Meridiosaurus: 1: 1
node 156: 0: 0.00001833, 1: 0.99894445*, 2: 0.00103721
Sarcosuchus: 1: 1
node 158: 0: 0.0000381, 1: 0.99614701*, 2: 0.00381489
node 159: 0: 0.00003376, 1: 0.99633958*, 2: 0.00362666
Pholidosaurus: 1: 1
Oceanosuchus: 2: 1
node 162: 0: 0.00275386, 1: 0.05092261, 2: 0.94632353*
Terminonaris browni: 2: 1
Terminonaris robusta: 2: 1
node 165: 0: 0.00091849, 1: 0.99901893*, 2: 0.00006258
Theriosuchus pusilis: 1: 1
Theriosuchus guimarotae: 1: 1
node 168: 0: 0.95171335*, 1: 0.04800214, 2: 0.00028451
node 169: 0: 0.90880552*, 1: 0.09002769, 2: 0.00116679
node 170: 0: 0.03464292, 1: 0.96450861*, 2: 0.00084847
node 171: 0: 0.00099181, 1: 0.99890784*, 2: 0.00010035
Mahajangasuchus: 1: 1
Kaprosuchus: 1: 1
Stolokrosuchus: 1: 1
node 175: 0: 0.99370652*, 1: 0.00583296, 2: 0.00046052
Lomasuchus: 0: 1
node 177: 0: 0.99989662*, 1: 0.00006808, 2: 0.00003529
Uberabasuchus: 0: 1
Peirosaurus: 0: 1
node 180: 0: 0.99829071*, 1: 0.00168047, 2: 0.00002882
node 181: 0: 0.99978104*, 1: 0.00015424, 2: 0.00006472
Araripesuchus gomesii: 0: 1
Araripesuchus patagonicus: 0: 1
node 184: 0: 0.99987225*, 1: 0.00011842, 2: 0.00000932
Urugaysuchus: 0: 1
node 186: 0: 0.99997571*, 1: 0.00001623, 2: 0.00000806
Libycosuchus: 0: 1
node 188: 0: 0.99997535*, 1: 0.00001277, 2: 0.00001188
Simosuchus: 0: 1
node 190: 0: 0.99999744*, 1: 0.00000129, 2: 0.00000128
Malawisuchus: 0: 1
node 192: 0: 0.99999733*, 1: 0.00000133, 2: 0.00000133
node 193: 0: 0.99990055*, 1: 0.00004972, 2: 0.00004972
Notosuchus: 0: 1
Mariliasuchus: 0: 1
node 196: 0: 0.99999928*, 1: 0.00000036, 2: 0.00000036
node 197: 0: 0.99999856*, 1: 0.00000072, 2: 0.00000072
Sphagesaurus: 0: 1
Chimaerasuchus: 0: 1
node 200: 0: 0.99993877*, 1: 0.00003061, 2: 0.00003061

Comahuesuchus: 0: 1
node 202: 0: 0.99999499*, 1: 0.0000025, 2: 0.0000025
Pissarrachampsa: 0: 1
Stratiotosuchus: 0: 1
node 205: 0: 0.99995944*, 1: 0.00003444, 2: 0.00000612
Calsoyasuchus: 0: 1
node 207: 0: 0.99992379*, 1: 0.00003853, 2: 0.00003768
Hsisosuchus dashanpuensis: 0: 1
Hsisosuchus chungkingensis: 0: 1
node 210: 0: 0.99999565*, 1: 0.00000178, 2: 0.00000257
node 211: 0: 0.99999892*, 1: 0.00000052, 2: 0.00000056
node 212: 0: 0.9999962*, 1: 0.0000019, 2: 0.0000019
node 213: 0: 0.99999978*, 1: 0.00000011, 2: 0.00000011
Protosuchus: 0: 1
Orthosuchus: 0: 1
Edentosuchus: 0: 1
node 217: 0: 0.99971395*, 1: 0.00014302, 2: 0.00014302
node 218: 0: 0.9998508*, 1: 0.0000746, 2: 0.0000746
Sichuanosuchus: 0: 1
Shantungosuchus: 0: 1
node 221: 0: 0.99950762*, 1: 0.00024619, 2: 0.00024619
Zosuchus: 0: 1
Fruitachampsa: 0: 1
node 224: 0: 0.99571718*, 1: 0.00214137, 2: 0.00214145
Zaraasuchus: 0: 1
Gobiosuchus: 0: 1
node 227: 0: 0.00256332, 1: 0.00005841, 2: 0.99737827*
node 228: 0: 0.00004935, 1: 0.00000524, 2: 0.99994542*
Pelagosaurus: 2: 1
node 230: 0: 0.00000205, 1: 0.00000181, 2: 0.99999614*
Teleidosaurus calvadosii: 2: 1
node 232: 0: 0.00000044, 1: 0.00000043, 2: 0.99999913*
Eoneustes bathonicus: 2: 1
node 234: 0: 0.00000033, 1: 0.00000033, 2: 0.99999935*
Eoneustes gaudryi: 2: 1
node 236: 0: 0.00000013, 1: 0.00000013, 2: 0.99999975*
Zoneait: 2: 1
node 238: 0: 0.00000064, 1: 0.00000064, 2: 0.99999872*
Metriorhynchus superciliosus: 2: 1
node 240: 0: 0.00000014, 1: 0.00000014, 2: 0.99999973*
node 241: 0: 0.000002, 1: 0.000002, 2: 0.999996*
Metriorhynchus leedsi: 2: 1
Cricosaurus suevicus: 2: 1
node 244: 0: 0.0000002, 1: 0.0000002, 2: 0.99999959*
node 245: 0: 0.00000004, 1: 0.00000004, 2: 0.99999991*
Metriorhynchus durobrivensis: 2: 1
Metriorhynchus casamiquelai: 2: 1
node 248: 0: 0.00000154, 1: 0.00000154, 2: 0.99999691*
node 249: 0: 0.00000088, 1: 0.00000088, 2: 0.99999825*
Geosaurus grandis: 2: 1
Geosaurus giganteus: 2: 1

node 252: 0: 0.00000183, 1: 0.00000183, 2: 0.99999635*
Dakosaurus maximus: 2: 1
Dakosaurus andiniensis: 2: 1
node 255: 0: 0.00008113, 1: 0.00000267, 2: 0.9999162*
Steneosaurus gracilirostris: 2: 1
node 257: 0: 0.00000622, 1: 0.00000128, 2: 0.9999925*
Teleosaurus: 2: 1
node 259: 0: 0.00000042, 1: 0.00000064, 2: 0.99999894*
node 260: 0: 0.00000045, 1: 0.00001611, 2: 0.99998344*
node 261: 0: 0.00001607, 1: 0.00131738, 2: 0.99866655*
node 262: 0: 0.00024325, 1: 0.97942435*, 2: 0.0203324
Peipehsuchus: 1: 1
Thai teleosaurid: 1: 1
node 265: 0: 0.00000015, 1: 0.00000528, 2: 0.99999457*
Platysuchus multiscrobiculatus: 2: 1
Steneosaurus bollensis: 2: 1
Steneosaurus brevior: 2: 1
node 269: 0: 0.00001214, 1: 0.00001215, 2: 0.99997571*
node 270: 0: 0.0000124, 1: 0.0000124, 2: 0.99997521*
Steneosaurus durobrivensis: 2: 1
Machimosaurus: 2: 1
Steneosaurus leedsi: 2: 1
Kayentasuchus: 0: 1
node 275: 0: 0.99973496*, 1: 0.00013229, 2: 0.00013275
Junggarsuchus: 0: 1
Almadasuchus: 0: 1
node 278: 0: 0.9999859*, 1: 0.00000705, 2: 0.00000705
node 279: 0: 0.9999739*, 1: 0.00001305, 2: 0.00001305
Dibothrosuchus: 0: 1
Sphenosuchus: 0: 1
Hesperosuchus: 0: 1
Postosuchus: 0: 1
Gracilisuchus: 0: 1

f) Gavialis and Tomistoma as living sister taxa (excluding thoracosaurs):

Reference tree:

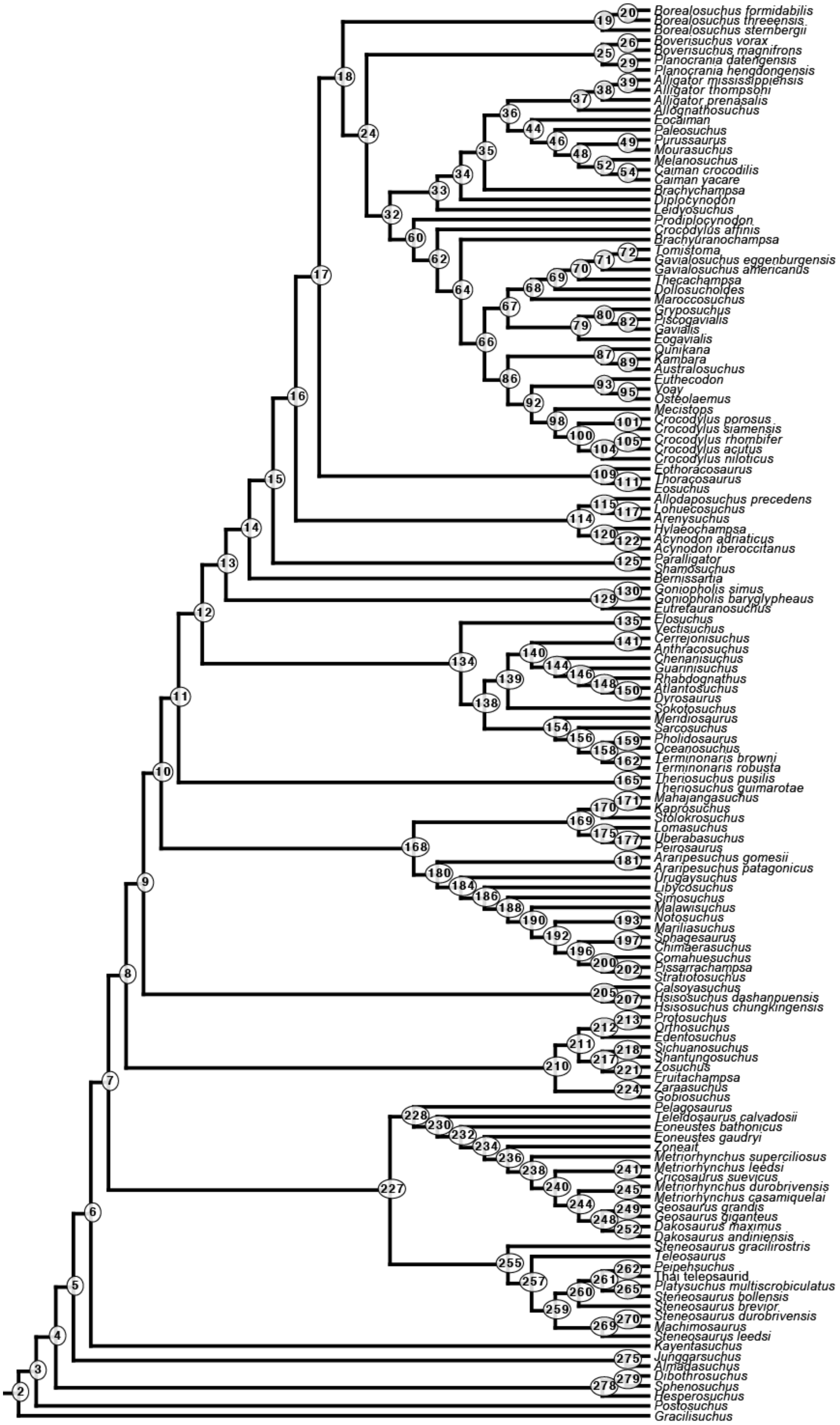

Character: Character 1
Marginal prob. recon. with model Mk1 (est.) [rate 0.00210206 [est.]] -log L.:64.64537642 (Opt.: width 0.0) Reporting likelihoods as Proportional Likelihoods; Threshold when decisions made: 2
Calc. by Maximum likelihood reconstruct (Generic categorical) (id# 2115)

node 2: 0: 0.99964794*, 1: 0.00017603, 2: 0.00017603
node 3: 0: 0.99995775*, 1: 0.00002112, 2: 0.00002113
node 4: 0: 0.99997978*, 1: 0.00001007, 2: 0.00001016
node 5: 0: 0.99997309*, 1: 0.00000753, 2: 0.00001938
node 6: 0: 0.99996436*, 1: 0.00000182, 2: 0.00003382
node 7: 0: 0.9994842*, 1: 0.00001372, 2: 0.00050209
node 8: 0: 0.9999868*, 1: 0.00000291, 2: 0.00001029
node 9: 0: 0.99890434*, 1: 0.00104224, 2: 0.00005343
node 10: 0: 0.75802498*, 1: 0.23974669*, 2: 0.00222833
node 11: 0: 0.0218063, 1: 0.97788713*, 2: 0.00030656
node 12: 0: 0.00030937, 1: 0.99967832*, 2: 0.00001231
node 13: 0: 0.00001596, 1: 0.99997884*, 2: 0.0000052
node 14: 0: 0.00005156, 1: 0.99989721*, 2: 0.00005123
node 15: 0: 0.00000776, 1: 0.99998229*, 2: 0.00000995
node 16: 0: 0.00000153, 1: 0.99999013*, 2: 0.00000834
node 17: 0: 0.00076312, 1: 0.98987715*, 2: 0.00935973
node 18: 0: 0.00050959, 1: 0.99880887*, 2: 0.00068154
node 19: 0: 0.00010263, 1: 0.99978389*, 2: 0.00011348
node 20: 0: 0.00005097, 1: 0.99989759*, 2: 0.00005144
Borealosuchus formidabilis: 1: 1
Borealosuchus threeensis: 1: 1
Borealosuchus sternbergii: 1: 1
node 24: 0: 0.00505178, 1: 0.99434159*, 2: 0.00060663
node 25: 0: 0.9909829*, 1: 0.00845255, 2: 0.00056456
node 26: 0: 0.99992995*, 1: 0.00005568, 2: 0.00001437
Boverisuchus vorax: 0: 1
Boverisuchus magnifrons: 0: 1
node 29: 0: 0.99984448*, 1: 0.00014147, 2: 0.00001405
Planocrania datengensis: 0: 1
Planocrania hengdongensis: 0: 1
node 32: 0: 0.00015048, 1: 0.9998215*, 2: 0.00002803
node 33: 0: 0.00001073, 1: 0.9999814*, 2: 0.00000787
node 34: 0: 0.00001163, 1: 0.99997679*, 2: 0.00001158
node 35: 0: 0.00000133, 1: 0.99999735*, 2: 0.00000132
node 36: 0: 0.0000142, 1: 0.99997161*, 2: 0.0000142
node 37: 0: 0.00000066, 1: 0.99999868*, 2: 0.00000066
node 38: 0: 0.00000217, 1: 0.99999565*, 2: 0.00000217
node 39: 0: 0.00002836, 1: 0.99994327*, 2: 0.00002836
Alligator mississippiensis: 1: 1
Alligator thompsoni: 1: 1
Alligator prenasalis: 1: 1
Allognathosuchus: 1: 1
node 44: 0: 0.00001055, 1: 0.9999789*, 2: 0.00001055
Eocaiman: 1: 1
node 46: 0: 0.00005285, 1: 0.9998943*, 2: 0.00005285
Paleosuchus: 1: 1

node 48: 0: 0.00001724, 1: 0.99996553*, 2: 0.00001724
node 49: 0: 0.00000707, 1: 0.99998585*, 2: 0.00000707
Purussaurus: 1: 1
Mourasuchus: 1: 1
node 52: 0: 0.00000507, 1: 0.99998985*, 2: 0.00000507
Melanosuchus: 1: 1
node 54: 0: 0.00000046, 1: 0.99999908*, 2: 0.00000046
Caiman crocodilis: 1: 1
Caiman yacare: 1: 1
Brachychampsa: 1: 1
Diplocynodon: 1: 1
Leidyosuchus: 1: 1
node 60: 0: 0.00002983, 1: 0.9999397*, 2: 0.00003047
Prodiplocynodon: 1: 1
node 62: 0: 0.00001112, 1: 0.99977385*, 2: 0.00021503
Crocodylus affinis: 1: 1
node 64: 0: 0.00001534, 1: 0.99884024*, 2: 0.00114442
Brachyuranochampsa: 1: 1
node 66: 0: 0.00014791, 1: 0.97652607*, 2: 0.02332602
node 67: 0: 0.00013806, 1: 0.02390701, 2: 0.97595493*
node 68: 0: 0.00000423, 1: 0.00033216, 2: 0.9996636*
node 69: 0: 0.00000338, 1: 0.00000877, 2: 0.99998786*
node 70: 0: 0.00001458, 1: 0.00001682, 2: 0.9999686*
node 71: 0: 0.00000996, 1: 0.00011056, 2: 0.99987948*
node 72: 0: 0.00003258, 1: 0.00073328, 2: 0.99923414*
Tomistoma: 1: 1
Gavialosuchus eggenburgensis: 2: 1
Gavialosuchus americanus: 2: 1
Thecachampsa: 2: 1
Dollosuchoides: 2: 1
Maroccosuchus: 2: 1
node 79: 0: 0.00005797, 1: 0.00130043, 2: 0.9986416*
node 80: 0: 0.00005689, 1: 0.00064624, 2: 0.99929687*
Gryposuchus: 2: 1
node 82: 0: 0.0002564, 1: 0.01214088, 2: 0.98760272*
Piscogavialis: 2: 1
Gavialis: 1: 1
Eogavialis: 2: 1
node 86: 0: 0.0001267, 1: 0.99958885*, 2: 0.00028446
node 87: 0: 0.00073812, 1: 0.99908859*, 2: 0.00017329
Qunikana: 0: 1
node 89: 0: 0.00003461, 1: 0.99995589*, 2: 0.0000095
Kambara: 1: 1
Australosuchus: 1: 1
node 92: 0: 0.00003103, 1: 0.99993005*, 2: 0.00003892
node 93: 0: 0.00002053, 1: 0.99995861*, 2: 0.00002085
Euthecodon: 1: 1
node 95: 0: 0.00001834, 1: 0.99996331*, 2: 0.00001835
Voay: 1: 1
Osteolaemus: 1: 1
node 98: 0: 0.00001444, 1: 0.99997107*, 2: 0.00001449

Mecistops: 1: 1
node 100: 0: 0.00000105, 1: 0.99999791*, 2: 0.00000105
node 101: 0: 0.00000191, 1: 0.99999619*, 2: 0.00000191
Crocodylus porosus: 1: 1
Crocodylus siamensis: 1: 1
node 104: 0: 0.00000115, 1: 0.99999771*, 2: 0.00000115
node 105: 0: 0.00000057, 1: 0.99999887*, 2: 0.00000057
Crocodylus rhombifer: 1: 1
Crocodylus acutus: 1: 1
Crocodylus niloticus: 1: 1
node 109: 0: 0.00314051, 1: 0.05766401, 2: 0.93919549*
Eothoracosaurus: 2: 1
node 111: 0: 0.0000343, 1: 0.00020446, 2: 0.99976124*
Thoracosaurus: 2: 1
Eosuchus: 2: 1
node 114: 0: 0.00000094, 1: 0.99999775*, 2: 0.00000131
node 115: 0: 0.00017955, 1: 0.99964088*, 2: 0.00017957
Allodaposuchus precedens: 1: 1
node 117: 0: 0.00007815, 1: 0.9998437*, 2: 0.00007815
Lohuecosuchus: 1: 1
Arenysuchus: 1: 1
node 120: 0: 0.00000101, 1: 0.99999796*, 2: 0.00000103
Hylaeochampsa: 1: 1
node 122: 0: 0.00022671, 1: 0.99954658*, 2: 0.00022671
Acynodon adriaticus: 1: 1
Acynodon iberoccitanus: 1: 1
node 125: 0: 0.00013946, 1: 0.99972092*, 2: 0.00013961
Paralligator: 1: 1
Shamosuchus: 1: 1
Bernissartia: 1: 1
node 129: 0: 0.00000314, 1: 0.99999396*, 2: 0.0000029
node 130: 0: 0.00000415, 1: 0.9999917*, 2: 0.00000414
Goniopholis simus: 1: 1
Goniopholis baryglypheaus: 1: 1
Eutretauranosuchus: 1: 1
node 134: 0: 0.0000448, 1: 0.99988009*, 2: 0.00007511
node 135: 0: 0.00022913, 1: 0.99953888*, 2: 0.00023199
Elosuchus: 1: 1
Vectisuchus: 1: 1
node 138: 0: 0.00016908, 1: 0.99853045*, 2: 0.00130046
node 139: 0: 0.00779244, 1: 0.1330184*, 2: 0.85918917*
node 140: 0: 0.00019641, 1: 0.0829774, 2: 0.91682619*
node 141: 0: 0.00008452, 1: 0.98977177*, 2: 0.01014371
Cerrejonisuchus: 1: 1
Anthracosuchus: 1: 1
node 144: 0: 0.00000245, 1: 0.00074334, 2: 0.99925422*
Chenanisuchus: 2: 1
node 146: 0: 0.00000003, 1: 0.00000492, 2: 0.99999505*
Guarinisuchus: 2: 1
node 148: 0: 2.92194458E-9, 1: 0.00000002, 2: 0.99999998*
Rhabdognathus: 2: 1

node 150: 0: 0.00000022, 1: 0.00000022, 2: 0.99999957*
Atlantosuchus: 2: 1
Dyrosaurus: 2: 1
Sokotosuchus: 2: 1
node 154: 0: 0.00000636, 1: 0.99988465*, 2: 0.00010899
Meridiosaurus: 1: 1
node 156: 0: 0.00002378, 1: 0.99883688*, 2: 0.00113934
Sarcosuchus: 1: 1
node 158: 0: 0.0000469, 1: 0.99604245*, 2: 0.00391065
node 159: 0: 0.0000411, 1: 0.99626203*, 2: 0.00369687
Pholidosaurus: 1: 1
Oceanosuchus: 2: 1
node 162: 0: 0.00329801, 1: 0.05556734, 2: 0.94113465*
Terminonaris browni: 2: 1
Terminonaris robusta: 2: 1
node 165: 0: 0.00108359, 1: 0.9988347*, 2: 0.00008172
Theriosuchus pusilis: 1: 1
Theriosuchus guimarotae: 1: 1
node 168: 0: 0.94793822*, 1: 0.05169385, 2: 0.00036793
node 169: 0: 0.90108757*, 1: 0.09749816, 2: 0.00141427
node 170: 0: 0.03763146, 1: 0.96135174*, 2: 0.0010168
node 171: 0: 0.00118378, 1: 0.99868478*, 2: 0.00013144
Mahajangasuchus: 1: 1
Kaprosuchus: 1: 1
Stolokrosuchus: 1: 1
node 175: 0: 0.99248936*, 1: 0.00690987, 2: 0.00060077
Lomasuchus: 0: 1
node 177: 0: 0.99986402*, 1: 0.00008889, 2: 0.00004709
Uberabasuchus: 0: 1
Peirosaurus: 0: 1
node 180: 0: 0.99795815*, 1: 0.00200291, 2: 0.00003894
node 181: 0: 0.99971232*, 1: 0.00020174, 2: 0.00008594
Araripesuchus gomesii: 0: 1
Araripesuchus patagonicus: 0: 1
node 184: 0: 0.99983195*, 1: 0.00015525, 2: 0.0000128
Urugaysuchus: 0: 1
node 186: 0: 0.9999665*, 1: 0.00002261, 2: 0.00001089
Libycosuchus: 0: 1
node 188: 0: 0.99996695*, 1: 0.00001722, 2: 0.00001583
Simosuchus: 0: 1
node 190: 0: 0.99999658*, 1: 0.00000172, 2: 0.0000017
Malawisuchus: 0: 1
node 192: 0: 0.99999646*, 1: 0.00000177, 2: 0.00000177
node 193: 0: 0.9998688*, 1: 0.0000656, 2: 0.0000656
Notosuchus: 0: 1
Mariliasuchus: 0: 1
node 196: 0: 0.99999903*, 1: 0.00000048, 2: 0.00000048
node 197: 0: 0.9999981*, 1: 0.00000095, 2: 0.00000095
Sphagesaurus: 0: 1
Chimaerasuchus: 0: 1
node 200: 0: 0.99991926*, 1: 0.00004037, 2: 0.00004037

Comahuesuchus: 0: 1
node 202: 0: 0.99999339*, 1: 0.0000033, 2: 0.0000033
Pissarrachampsa: 0: 1
Stratiotosuchus: 0: 1
node 205: 0: 0.9999471*, 1: 0.00004464, 2: 0.00000825
Calsoyasuchus: 0: 1
node 207: 0: 0.99989935*, 1: 0.00005092, 2: 0.00004972
Hsisosuchus dashanpuensis: 0: 1
Hsisosuchus chungkingensis: 0: 1
node 210: 0: 0.99999408*, 1: 0.00000242, 2: 0.0000035
node 211: 0: 0.9999985*, 1: 0.00000072, 2: 0.00000078
node 212: 0: 0.99999495*, 1: 0.00000252, 2: 0.00000253
node 213: 0: 0.9999997*, 1: 0.00000015, 2: 0.00000015
Protosuchus: 0: 1
Orthosuchus: 0: 1
Edentosuchus: 0: 1
node 217: 0: 0.99961555*, 1: 0.00019222, 2: 0.00019223
node 218: 0: 0.99980264*, 1: 0.00009868, 2: 0.00009868
Sichuanosuchus: 0: 1
Shantungosuchus: 0: 1
node 221: 0: 0.99934282*, 1: 0.00032859, 2: 0.00032859
Zosuchus: 0: 1
Fruitachampsa: 0: 1
node 224: 0: 0.99432027*, 1: 0.00283981, 2: 0.00283992
Zaraasuchus: 0: 1
Gobiosuchus: 0: 1
node 227: 0: 0.00281966, 1: 0.00007044, 2: 0.99710991*
node 228: 0: 0.00005982, 1: 0.0000069, 2: 0.99993329*
Pelagosaurus: 2: 1
node 230: 0: 0.0000027, 1: 0.00000239, 2: 0.9999949*
Teleidosaurus calvadosii: 2: 1
node 232: 0: 0.00000058, 1: 0.00000058, 2: 0.99999884*
Eoneustes bathonicus: 2: 1
node 234: 0: 0.00000043, 1: 0.00000043, 2: 0.99999914*
Eoneustes gaudryi: 2: 1
node 236: 0: 0.00000017, 1: 0.00000017, 2: 0.99999967*
Zoneait: 2: 1
node 238: 0: 0.00000084, 1: 0.00000084, 2: 0.99999831*
Metriorhynchus superciliosus: 2: 1
node 240: 0: 0.00000018, 1: 0.00000018, 2: 0.99999964*
node 241: 0: 0.00000263, 1: 0.00000263, 2: 0.99999473*
Metriorhynchus leedsi: 2: 1
Cricosaurus suevicus: 2: 1
node 244: 0: 0.00000027, 1: 0.00000027, 2: 0.99999946*
node 245: 0: 0.00000006, 1: 0.00000006, 2: 0.99999989*
Metriorhynchus durobrivensis: 2: 1
Metriorhynchus casamiquelai: 2: 1
node 248: 0: 0.00000204, 1: 0.00000204, 2: 0.99999593*
node 249: 0: 0.00000115, 1: 0.00000115, 2: 0.99999769*
Geosaurus grandis: 2: 1
Geosaurus giganteus: 2: 1

node 252: 0: 0.00000241, 1: 0.00000241, 2: 0.99999519*
Dakosaurus maximus: 2: 1
Dakosaurus andiniensis: 2: 1
node 255: 0: 0.00009821, 1: 0.00000354, 2: 0.99989824*
Steneosaurus gracilirostris: 2: 1
node 257: 0: 0.00000822, 1: 0.00000172, 2: 0.99999006*
Teleosaurus: 2: 1
node 259: 0: 0.00000057, 1: 0.00000085, 2: 0.99999858*
node 260: 0: 0.0000006, 1: 0.00001935, 2: 0.99998005*
node 261: 0: 0.00001931, 1: 0.00144147, 2: 0.99853923*
node 262: 0: 0.00029185, 1: 0.97744593*, 2: 0.02226222
Peipehsuchus: 1: 1
Thai teleosaurid: 1: 1
node 265: 0: 0.00000019, 1: 0.00000634, 2: 0.99999347*
Platysuchus multiscrobiculatus: 2: 1
Steneosaurus bollensis: 2: 1
Steneosaurus brevior: 2: 1
node 269: 0: 0.00001605, 1: 0.00001606, 2: 0.99996789*
node 270: 0: 0.00001639, 1: 0.00001639, 2: 0.99996723*
Steneosaurus durobrivensis: 2: 1
Machimosaurus: 2: 1
Steneosaurus leedsi: 2: 1
Kayentasuchus: 0: 1
node 275: 0: 0.99964973*, 1: 0.0001748, 2: 0.00017546
Junggarsuchus: 0: 1
Almadasuchus: 0: 1
node 278: 0: 0.99998134*, 1: 0.00000933, 2: 0.00000933
node 279: 0: 0.9999656*, 1: 0.0000172, 2: 0.0000172
Dibothrosuchus: 0: 1
Sphenosuchus: 0: 1
Hesperosuchus: 0: 1
Postosuchus: 0: 1
Gracilisuchus: 0: 1

III) Stratigraphic ranges for study taxa.

Taxon	Range - lower	Range - upper	midpoint
Acynodon adriaticus	84.9	66.043	75.4715
Acynodon iberoccitanus	70.6	66.043	68.3215
Alligator mississippiensis	0	0	0
Alligator prenasalis	37.2	33.9	35.55
Alligator thompsoni	20.43	15.97	18.2
Allodaposuchus precedens	83.5	66	74.75
Allognathosuchus	46.2	40	43.1
Almadasuchus	161.2	155.7	158.45
Anthracosuchus	61.7	55.8	58.75
Araripesuchus gomesii	112	109	110.5
Araripesuchus patagonicus	99.6	93.5	96.55
Arenysuchus	70.6	66.043	68.3215
Atlantosuchus	66.043	61.7	63.8715
Australosuchus	28.4	15.97	22.185
Bernissartia	129.4	122.5	125.95
Borealosuchus formidabilis	61.7	56.8	59.25
Borealosuchus sternbergii	66.043	63.3	64.6715
Borealosuchus threensis	70.6	61.7	66.15
Boverisuchus magnifrons	48.6	40.4	44.5
Boverisuchus vorax	55.8	50.3	53.05
Brachychampsa	70.6	66.043	68.3215
Brachyuranochampsa	46.2	40.4	43.3
Caiman crocodilis	0	0	0
Caiman yacare	0	0	0
Calsoyasuchus	199.3	182.7	191
Cerrejonisuchus	61.7	58.7	60.2
Chenanisuchus	70.6	55.8	63.2
Chimaerasuchus	126	100	113
Comahuesuchus	86.3	83.6	84.95
Cricosaurus suevicus	155.7	150.8	153.25
Crocodylus acutus	0	0	0
Crocodylus affinis	50.3	46.2	48.25
Crocodylus niloticus	0	0	0
Crocodylus porosus	0	0	0
Crocodylus rhombifer	0	0	0
Crocodylus siamensis	0	0	0
Dakosaurus andiniensis	152.1	145	148.55
Dakosaurus maximus	157.3	152.1	154.7
Dibothrosuchus	201.5	190	195.75
Diplocynodon	55.8	28.4	42.1
Dollosuchoides	48.6	40.4	44.5
Dyrosaurus	56	47.8	51.9
Edentosuchus	126	100	113
Elosuchus	99.6	93.5	96.55
Enaliosuchus schroederi	145.5	136.4	140.95
Eocaiman	55.8	33.9	44.85

Eogavialis	37.2	33.9	35.55
Eoneustes bathonicus	167.7	164.7	166.2
Eoneustes gaudryi	167.7	164.7	166.2
Eosuchus	55.8	48.6	52.2
Eothoracosaurus	70.6	66	68.3
Euthecodon	23.03	15.97	19.5
Eutretauranosuchus	157.3	152.1	154.7
Fruitachampsa	157	152	154.5
Gavialis	0	0	0
Gavialosuchus americanus	11.6	2.6	7.1
Gavialosuchus eggenburgensis	20.43	15.97	18.2
Geosaurus giganteus	150.8	145.5	148.15
Geosaurus grandis	150.8	145.5	148.15
Gobiosuchus	83.5	70.6	77.05
Goniopholis baryglypheaus	157.3	152.1	154.7
Goniopholis simus	145	139.8	142.4
Gracilisuchus	242	235	238.5
Gryposuchus	15.97	11.8	13.885
Guarinisuchus	66.043	61.7	63.8715
Hesperosuchus	221.5	205.6	213.55
Hsisosuchus chungkingensis	163.5	145	154.25
Hsisosuchus dashanpuensis	171.6	161.2	166.4
Hylaeochampsa	130	122.5	126.25
Junggarsuchus	164.7	161.2	162.95
Kambara	48.6	33.9	41.25
Kaprosuchus	100.5	93.9	97.2
Kayenta form	197	183	190
Kayentasuchus	196.5	183	189.75
Leidyosuchus	83.5	70.6	77.05
Libycosuchus	99.6	93.5	96.55
Lohuecosuchus	70.6	66.043	68.3215
Lomasuchus	93.5	85.8	89.65
Machimosaurus	155.7	150.8	153.25
Mahajangasuchus	72.1	66	69.05
Malawisuchus	126	113	119.5
Mariliasuchus	83.6	72.1	77.85
Maroccosuchus	55.8	48.6	52.2
Mecistops	0	0	0
Melanosuchus	0	0	0
Meridiosaurus	157.3	145	151.15
Metriorhynchus casamiquelai	164.7	161.2	162.95
Metriorhynchus durobrivensis	164.7	161.2	162.95
Metriorhynchus leedsi	164.7	150.8	157.75
Metriorhynchus superciliosus	164.7	161.2	162.95
Mourasuchus	15.97	5.332	10.651
Notosuchus	86.3	83.6	84.95
Oceanosuchus	99.6	93.5	96.55
Orthosuchus	201.5	190	195.75
Osteolaemus	0	0	0

Paleosuchus	0	0	0
Paralligator	99.7	89.3	94.5
Peipehsuchus	182.7	174.1	178.4
Peirosaurus	70.6	66	68.3
Pelagosaurus	183	182	182.5
Pholidosaurus	145	139.8	142.4
Piscogavialis	7.246	5.332	6.289
Pissarrachampsa	94.3	66.043	80.1715
Planocrania datengensis	61.7	55.87	58.785
Planocrania hengdongensis	58.7	55.8	57.25
Platysuchus multiscrobiculatus	183	182	182.5
Postosuchus	221.5	205.6	213.55
Prodiplocynodon	70.6	66.043	68.3215
Protosuchus	201.3	197	199.15
Purussaurus	13.8	5.332	9.566
Quinkana	28.4	11.608	20.004
Rhabdognathus	72.1	61.6	66.85
Sarcosuchus	122.5	109	115.75
Shamosuchus	83.5	70.6	77.05
Shantungosuchus	145	100	122.5
Sichuanosuchus	145	100	122.5
Simosuchus	72.1	66	69.05
Sokotosuchus	70.6	66	68.3
Sphagesaurus	100	72.1	86.05
Sphenosuchus	201.6	189.6	195.6
Steneosaurus bollensis	183	182	182.5
Steneosaurus brevior	183	182	182.5
Steneosaurus durobrivensis	164.7	161.2	162.95
Steneosaurus gracilirostris	183	175.6	179.3
Steneosaurus leedsi	164.7	161.2	162.95
Stolokrosuchus	125.45	99.7	112.575
Stratiotosuchus	84.9	66.043	75.4715
Teleidosaurus calvadosi	171.6	164.7	168.15
Teleosaurus	167.7	150.8	159.25
Terminonaris browni	94.3	89.3	91.8
Terminonaris robusta	100.5	89.8	95.15
Thai teleosaurid	152.1	145	148.55
Thecachampsa	28.4	23.03	25.715
Theriosuchus guimarotae	157.3	152.1	154.7
Theriosuchus pusilis	145	140.2	142.6
Thoracosaurus	66.043	55.8	60.9215
Tomistoma	0	0	0
Uberabasuchus	70.6	66	68.3
Urugaysuchus	126	113	119.5
Vectisuchus	130	122.46	126.23
Voay	0.012	0	0.006
Zaraasuchus	83.6	72.1	77.85
Zoneait	175.6	168.4	172
Zosuchus	83.6	72.1	77.85

IV) Material and references used for phylogenetic character scoring.

Gracilisuchus stipanicicorum – MCZ 4117; MCZ 4118; Romer 1972; Leucona and Desojo 2011
Postosuchus kirkpatricki – Weinbaum 2002, 2011
Hesperosuchus agilis – CM 29894; Clark et al. 2000
Kayentasuchus walker – Clark and Sues 2002
Junggarsuchus sloani – IVPP V14010 (holotype); Clark et al. 2004
Almadasuchus figarii – Pol et al. 2013
Sphenosuchus acutus – Walker 1990
Dibothrosuchus elaphros – Wu and Chaterjee 1993
Zaraasuchus shepardi – Pol and Norell 2004b
Gobiosuchus kielanae – Osmolska et al. 1997
Orthosuchus stormbergi – Nash 1975
Protosuchus richardsoni – MCZ 6727; AMNH 3024 (holotype); Colbert and Mook 1951
Shantungosuchus hangjinensis – Wu et al. 1994
Sichuanosuchus shuhanensis – IVPP V10594 (holotype); IVPP V12088; Wu et al. 1997
Zossuchus davidsoni – Pol and Norell 2004a
Kayenta Form – Clark 1986
Edentosuchus tienshanensis – IVPP V3236; Pol et al. 2004b
Fruitachampsa callisoni – Clark 2011
Hsisosuchus chunkingensis – IVPP V703 (holotype); Li et al. 2001
Hsisosuchus dashanpuensis – Gao, 2001
Simosuchus clarki – Buckley et al. 2000; Kley et al. 2010; Georgi and Krause 2010; Sertich and Groenke 2010; Hill 2010
Libycosuchus brevirostris – BSPG 1012 VIII 574 (holotype); MNHN MRS 3101; MNHN MRS 3102
Araripesuchus gomesii - AMNH 24450 (holotype)
Araripesuchus patagonicus - Ortega et al. 2000
Malawisuchus mwakayasyungutiensis – Gomani 1997
Uruguaysuchus aznarezi – Gasparini 1971
Notosuchus terrestris - NHMUK PV R14105 (cast); Pol 2005; Fiorelli and Calvo 2008
Mariliasuchus amarali – Zaher et al. 2006
Comahuesuchus brachybuccalis – Martinelli 2003
Chimaerasuchus paradoxus – IVPP V8274 (holotype); Wu and Sues 1996
Sphagesaurus heuni – Pol 2003
Lomasuchus palpebrosus - Gasparini et al. 1991
Uberabasuchus terrificus – Carvalho et al. 2004
Pissarrachampsa sera – Montefeltro et al. 2011
Striatosuchus maxhechti – Pinheiro et al. 2008; Riff and Kellner 2011
Peirosaurus torminni - Gasparini et al. 1991
Mahajangasuchus insignis – FMNH PR 2389; FMNH PR 2449; Buckley and Brochu 1999; Turner and Buckley 2008
Kaprosuchus saharicus – Sereno and Larsson 2009

Stolokrosuchus lapparenti – Larsson and Gado 2000
Montsecosuchus depereti – Buscalioni and Sanz 1990
Theriosuchus guimarotae – Schwarz and Salisbury 2005
Theriosuchus pusillus - NHMUK PV R48330 (holotype); NHMUK PV 48216; NHMUK PV 48318; NHMUK PV 48328; NHMUK PV48244; NHMUK PV 48266; Clark 1986
Hylaeochampsa vectiana – NHMUK PV R 177 (holotype); Clark and Norell 1992
Bernissartia fagesii – IRSNB n° R 46 (lectotype); Norell and Clark 1990
Shamosuchus djadochtaensis - Pol et al. 2009
Borealosuchus formidabilis – Erickson 1976; Brochu 1997b
Allodaposuchus precedens – Buscalioni et al. 2001
Gavialis gangeticus – NHMUK PV 61.4.1.2; Jouve 2009; Brochu 1997a
Alligator mississippiensis – Jouve 2009; Brochu 1999
Crocodylus niloticus – unnumbered SUI specimen; Jouve 2009
Susisuchus anatoceps – Salisbury et al. 2003
Eutretauranosuchus delfsi – Mook 1967; Smith et al. 2010; Pritchard et al. 2013
Goniopholis simus - NHMUK PV R 41098; NHMUK PV R 5814; NHMUK PV R 14155; NHMUK PV R 3876; NHMUK PV R 48310; NHMUK PV R 43598; NHMUK PV R 1956; NHMUK PV R 5138; NHMUK PV R 214; NHMUK PV R 3220; NHMUK PV R 48307; NHMUK PV R 48307; NHMUK PV R 3876; Salisbury et al. 1999
Goniopholis baryglyphaeus – Schwarz 2002
Vectisuchus leptognathus - SMNS 50984 (holotype); Buffetaut and Hutt 1980.
Calsoyasuchus valliceps – Tykoski et al. 2002
Elosuchus cherifiensis - MNHN SAM 129; Lapparent de Broin 2002
Chenanisuchus lateroculi – CNRST-SUNY 280; BSPG 2001 I40; Jouve et al. 2005
Sokotosuchus ianwilsoni – Buffetaut 1979
Rhabdognathus aslerensis – CNRST-SUNY 190; Brochu et al. 2002; Jouve 2007
Dyrosaurus phosphaticus – MNHN ALG 1; MNHN ALG 2; MNHN ALG 3; MNHN 1901-11 (holotype); MNHN APH 27; BSPG 2001 I 38; Jouve 2005
Hyposaurus rogersii – NJSM 12251; NJSM 11882; NJSM 6625; NJSM 11069; NJSM 10861; NJSM 10416; YPM 985; YPM 983; YPM 380; YPM 753. YPM 323; Denton et al. 1997
Guarinisuchus munizi – Barbosa et al. 2008
Atlantosuchus coupatezi – BRSLI unnumbered (holotype); Jouve et al. 2008
Cerrejonisuchus improcerus – Hastings et al. 2010
Anthracosuchus balrogus – Hastings et al. 2014
Meridiosaurus vallisparadisi – Fortier et al. 2011
Sarcosuchus imperator – MNHN GDF 662; Sereno et al. 2001
Pholidosaurus purbeckensis – NHMUK PV 28432; NHMUK PV R3414; NHMUK PV R3956; Salisbury et al. 2002
Oceanosuchus boecensis – MHNH 9036 (holotype)
Terminonaris browni – AMNH 5851 (holotype); AMNH 5844; Mook 1933
Terminonaris robusta – SMNH P2411.1; Wu et al. 2001
Steneosaurus brevior – NHMUK PV 14781 (holotype); NHMUK PV R756; UH 7; Mueller-Töwe 2006
Steneosaurus brevidens – NHMUK PV R8576

Steneosaurus bollensis – MCZ 1063; SMNS 20280; SMNS 4554; SMNS 4168; SMNS 15951b; SMNS 52034; GPIT Re 1193/1; GPIT Re 1193/2; GPIT Re 1193/3; GPIT Re 1193/10; GPIT Re 1193/13; Mueller-Töwe 2006
Steneosaurus durobrivensis – NHMUK PV R 2073; NHMUK PV R2865; NHMUK PV R3701 (holotype); Andrews 1913
Steneosaurus leedsi – NHMUK PV R3806; NHMUK PV R3320 (holotype); Andrews 1913
Pelagosaurus typus – UH 4; UH 8; UH 9; UH 10; BSPG 1973 VII 592; BSPG 1925 I 34; NHMUK PV 32599; Mueller-Töwe 2006
Peipehsuchus teleorhinus – IVPP R48001 (holotype); IVPP 10098
Phu Noi teleosaur – Photographs of PRC 239; PRC 238; PRC 7; PRC 8; PRC 9; Martin et al. 2015
Teleosaurus cadomensis – Jouve 2009
Platysuchus multiscrobiculatus – SMNS 9930 (holotype); UH 1; Mueller-Töwe 2006
Machimosaurus buffetauti – SMNS 91415 (holotype)
Teleidosaurus calvadosi – NHMUK PV R2681 (plastotype)
Eoneustes bathonicus – Mercier 1933
Eoneustes gaudryi – NHMUK PV 3353 (holotype)
Zoneait nargorum – UOMNCH F39539 (holotype); USNM 244292; USNM 244476; USNM 256441
Gracilineustes leedsi – NHMUK PV R3540 (holotype); NHMUK PV R3899; Andrews 1913
"Metriorhynchus" casamiquelai - Gasparini and Chong Díaz 1977
Suchodus durobrivensis – NHMUK PV R2039; NHMUK PV R2618; Andrews 1913
Suchodus brachyrhynchus – NHMUK PV R3699; NHMUK PR R3700; NHMUK PV R3939; Andrews 1913
Metriorhynchus superciliosus – NHMUK PV R2030; NHMUK PV R2036; NHMUK PV R2041; NHMUK PV R2051; NHMUK PV R6859; MNHN 1908-6; MNHN 8925; MNHN 8922
Cricosaurus suevicus – SMNS 9808; SMNS 90513
Enaliosuchus macrospondylus – Hua et al. 2000
Dakosaurus andiniensis – Pol and Gasparini 2009
Dakosaurus maximus – SMNS 8203 (neotype); SMNS 10819a; SMNS 10819b; SMNS 82043 Fraas 1902
Geosaurus giganteues – NHMUK PV R1229 (holotype); NHMUK PV R1230; NHMUK PV R37020; Young and Andrade 2009
Geosaurus grandis – BSPG AS-VI-1 (holotype)
Torvoneustes coryphaeus – Young et al. 2013
Steneosaurus gracilirostris – NHMUK PV R14372 (holotype); NHMUK PV R15500; NHMUK PV R33095; Mueller-Töwe 2006

V) Character list and state descriptions of the data matrix used in the phylogenetic analysis

Characters are arranged by anatomical region. The character list was assembled from numerous sources. Many of the characters have been reworded or modified from their original source. The original publication from which the character was taken is listed in the right column. The original character number is in parentheses. Characters treated as additive for the ordered-character analysis are denoted by "ORDERED" following the character description. Characters new to this analysis denoted by "NEW". Notes on applicability or other comments follow the description in italics.

Rostrum

Char. Number	Character description	Original publication
1.	Sculpture of external surface of rostrum:<br>0: absent or very reduced (slight grooves)<br>1: present as deep pits or grooves	Gasparini et al. 2006 (252)
2.	Rostral proportions at orbits:<br>0: rostrum narrows gradually anterior to orbits<br>1: rostrum narrows abruptly at orbits as in *Gavialis gangeticus; Protosuchus richardsoni*	Clark 1994 (2)
3.	Rostral length measured from anterior orbital edge to anterior contour of rostrum:<br>0: equal to or longer than remainder of skull as measured to the posterior end of the quadrate<br>1: shorter than remainder of skull	Ortega et al. 2000 (3)
4.	Rostral length measured from anterior orbital edge to anterior contour of rostrum:<br>0: equal to or slightly longer than distance from anterior orbital edge to posterior parietal contour<br>1: at least twice the distance from anterior orbital edge to posterior parietal contour	Ortega et al. 2000 (4)
5.	Lateral contour of maxilla in dorsal view:<br>0: straight or gently convex<br>1: sinusoidal	Ortega et al. 2000 (130)
6.	Elements contributing to dorsal border of external nares:<br>0: formed primarily by nasals with very little or no contribution from premaxilla<br>1: both the nasals and premaxilla<br>2: premaxilla only	Wu et al. 2001 (13)
7.	Orientation of external naris:<br>0: anteriorly or anterolaterally directed<br>1: dorsally or anterodorsally, located at anterior tip of snout,	Clark 1994 (6)

	with little or no premaxilla visible at anterior margin (or perinarial fossa extends to anterior margin of premaxilla as in *Steneosaurus bollensis*)<br>2: dorsally, located posterior to anterior tip of snout, with premaxilla broadly visible anterior to naris	
8.	Shape of external nares in dorsal view:<br>0: wider than long<br>1: subequal<br>2: much longer than wide as in *Cricosaurus suevicus*	Jouve 2009 (309)
9.	Septum dividing external naris:<br>0: present – formed partially by nasals<br>1: present – formed by premaxilla only<br>2: absent – external naris confluent	Clark 1994 (66)
10.	Notch in premaxilla on lateral edge of external nares:<br>0: absent<br>1: present on dorsal half	Pol 1999 (135)
11.	Dorsal projection of premaxilla (at suture between left and right premaxillae) at anterior margin of external nares:<br>0: absent<br>1: present (may contribute to internarial bar if present)	Jouve 2004 (3)
12.	Premaxilla contribution to internarial bar (*inapplicable in taxa lacking an internarial bar*)<br>0: forming at least ventral half<br>1: little, if any, contribution	Wu et al. 2001 (125)
13.	Maximal width of premaxillae relative to maximal width of rostrum at level of fourth or fifth maxillary alveoli:<br>0: premaxilla narrower<br>1: premaxilla broader as in *Sarcosuchus imperator*	Jouve 2009 (341)
14.	Premaxilla-maxilla contact:<br>0: loosely overlies maxilla (i.e. posterodorsal process of premaxilla overlaps anterodorsal surface of maxilla)<br>1: premaxilla and maxilla sutured together along butt joint	Clark 1994 (8)
15.	Premaxillo-maxillary suture direction in lateral view:<br>0: vertically directed<br>1: posterodorsally directed	Ortega et al. 2000 (6)
16.	Premaxillo-maxillary suture shape in lateral view:<br>0: straight<br>1: zigzag shaped	Ortega et al. 2000 (8)
17.	Premaxillo-maxillary suture direction in palatal view (direction of suture is evaluated with respect to a theoretical line that passes between the lateral contact of both bones):<br>0: anteriorly directed<br>1: sinusoidal, posteromedially directed on its lateral half and	Ortega et al. 2000 (9)

	anteromedially directed along its medial region<br>2: posteriorly directed<br>3: perpendicular to the longitudinal axis of the skull	
18.	Foramen at premaxillo-maxillary suture in lateral surface (not for large mandibular teeth):<br>0: absent<br>1: present as in *Simosuchus clarki*	Pol and Norell 2004a (135)
19.	Ventrally opening notch at premaxilla/maxilla contact for acceptance of enlarged dentary tooth (or teeth):<br>0: absent, snout not constricted at premaxilla/maxilla contact<br>1: present as a laterally open notch, snout constricted at premaxilla/maxilla contact as in *Crocodylus niloticus*<br>2: snout broad at contact with premaxilla and maxilla with notch opening dorsally as a large foramen as in *Dibothrosuchus*	Clark 1994 (9)
20.	Ventral edge of maxilla in lateral view:<br>0: straight or convex<br>1: sinusoidal	Ortega et al. 2000 (21)
21.	Maxillary fossa on posterolateral surface of maxilla:<br>0: absent<br>1: present as enlarged unsculptured area on maxilla only with multiple neurovascular foramina as in *Goniopholis simus*<br>2: present as elongate unsculptured groove beginning on jugal and extending onto maxilla with enlarged foramen or foramina exiting jugal as in *Sarcosuchus*	Wu et al. 2001 (127)
22.	Groove along lateral margin of maxilla dorsal to toothrow (separates sculptured region from unscluptured region):<br>0: absent<br>1: present as in *Sphagesaurus huenei*, *Terminonaris robusta*	Wilberg 2015a (346)
23.	Large and aligned neurovascular foramina on lateral maxillary surface:<br>0: absent<br>1: present	Pol, 1999 (152)
24.	Position of anterior portion of maxillary tooth row in relation to dentary tooth row:<br>0: adjacent to<br>1: offset labially and ventrally as in *Comahuesuchus*	Sereno et al. 2003 (75)
25.	Posterior extent of posterior process of maxilla:<br>0: terminating posterior to anterior margin of orbit<br>1: anterior to orbit	Wu et al. 2001 (114)
26.	Antorbital fenestra (ORDERED) (*metriorhynchoid taxa*	Clark 1994 (67)

	possessing a preorbital fenestra are coded as absent – these openings are not considered homologous):<br>0: as large as orbit<br>1: about half the diameter of orbit<br>2: much smaller than orbit<br>3: absent	
27.	Jugal participation in antorbital fenestra/fossa (*inapplicable for taxa lacking an antorbital fenestra*):<br>0: present - participates in margin<br>1: absent - excluded from fenestra	Ortega et al. 2000 (71)
28.	Jugal participation in preorbital fenestra/fossa (*inapplicable in taxa lacking a preorbital fenestra*)<br>0: present<br>1: absent – excluded or nearly excluded by long ventral process of lacrimal along ventral margin of preorbital fossa as in *Torvoneustes coryphaeus*	Wilberg, 2017
29.	Maxilla–lacrimal contact in antorbital region (*inapplicable in taxa lacking an antorbital fenestra or preorbital fenestra*):<br>0: partially included in antorbital fossa<br>1: completely included in antorbital fossa<br>2: completely included in preorbital fenestra	Pol 1999 (145)
30.	Nasal contact with premaxilla:<br>0: present<br>1: absent	Clark 1994 (14)
31.	Nasal lateral border near premaxilla/maxilla/nasal junction (*inapplicable in taxa lacking premaxilla-nasal contact*):<br>0: anterior portion of nasal laterally concave posterior to external nares (nasal may send a small lateral process between maxilla and premaxilla as *in Orthosuchus stormbergi*)<br>1: premaxilla-maxilla suture straight, continuous with the nasal-maxilla suture	Pol 1999 (140)
32.	Nasal contact with lacrimals:<br>0: nasal extensively contacts lacrimal<br>1: lacrimo-nasal contact excluded (or very nearly) by anterior projection of prefrontal meeting posterior projection of maxilla as in *Orthosuchus stormbergi*	Clark 1994 (11)
33.	Lacrimal contact with nasal (*inapplicable in taxa lacking extensive contact between lacrimal and nasal*):<br>0: contacting nasal along medial edge only	Clark 1994 (12)

	1: along medial and anterior edges	
34.	Nasal orientation at posterior border:<br>0: nasals converge at sagittal plane posteriorly<br>1: nasals separated posteriorly by an anterior sagittal projection of frontal	Ortega et al. 2000 (24)
35.	Distance between the posterior processes of nasals relative to the distance from the posterior process of the nasal to the anterior margin of the supratemporal fossa (*inapplicable in taxa lacking an anterior process of frontal separating the nasals posteriorly*):<br>0: much shorter<br>1: nearly as long	Jouve 2009 (312)
36.	Nasal bones:<br>0: paired<br>1: partially or completely fused as in *Dyrosaurus phosphaticus*	Gasparini et al. 2006 (257)
37.	Maximal width of the nasals relative to the minimal width of the snout in dorsal view:<br>0: narrower than or nearly as wide<br>1: wider than<br>2: more than twice as wide	Jouve 2009 (311)
38.	Posterolateral region of nasals:<br>0: flat or slightly convex surface facing dorsally<br>1: lateral region deflected ventrally, forming part of the lateral surface of the snout as in *Metriorhynchus superciliosus*	Pol and Apesteguía 2005 (223)
39.	Midline depression at contact between anterior process of frontal and nasals extending as a groove between nasals:<br>0: absent<br>1: present as in *Metriorhynchus superciliosus*	Wilberg 2015b (39)
40.	Anterior extent of lacrimal relative to anterior margin of antorbital fenestra (*inapplicable in taxa lacking an antorbital fenestra*):<br>0: does not exceed<br>1: exceeds	Jouve 2009 (314)
41.	Anterior extent of anterior process of prefrontal relative to posterior margin of antorbital fenestra (*inapplicable in taxa lacking an antorbital fenestra*):<br>0: reaches or exceeds<br>1: remains posterior	Jouve 2009 (317)
42.	Anterior extent of anterior process of jugal relative to anterior extent of lacrimal:<br>0: does not exceed	Jouve 2009 (318)

	1: exceeds	
43.	Anterior process of jugal contacting nasal, separating maxilla from lacrimal:<br>0: absent<br>1: present as in *Terminonaris robusta*	Wilberg 2015a (337)
44.	Prefrontal contact with nasal:<br>0: along medial edge only<br>1: penetrates the nasal anteriorly, separating the nasal into posteromedial and a posterolateral (or posteroventrolateral) processes as in *Metriorhynchus superciliosus*	Jouve 2009 (254)
45.	Anterior process of prefrontal relative to anterior process of lacrimal:<br>0: shorter<br>1: reaches or exceeds anteriorly	Wilberg 2015a (338)
46.	Nasal-prefrontal suture with a pronounced, rectangular 'concavity' (directed posteriorly) :<br>0: absent<br>1: present as in *Eoneustes gaudryi*	Young et al. 2012 (53)
47.	Prefrontal medial extent:<br>0: prefrontals do not meet at midline<br>1: prefrontals meet (or very nearly meet) at midline, excluding frontal contact with nasals (at least externally) as in *Pissarachampsa*	Wilberg 2015b (47)
48.	Posterior extent of posterior process of prefrontal relative to the anterior margin of the supratemporal fossa:<br>0: does not reach<br>1: reaches or very nearly reaches	Jouve 2009 (316)
49.	Prefrontal anterior process:<br>0: two anterior processes, one anterodorsal and one anteroventral, separated by posterodorsal process of lacrimal<br>1: single short anterior process (shorter than or as long as the orbit)<br>2: single long anterior process (longer than the orbit)	Gomani 1997 (4)
50.	Anterior extent of anterior process of frontal relative to anterior process of lacrimal:<br>0: much shorter than lacrimal<br>1: subequal or exceeds anterior extent of lacrimal	Wu et al. 2001 (129)

Skull roof

51.	Ornamentation of external surface of frontal and parietal:<br>0: smooth	Clark 1994 (1)

	1: formed by grooves and ridges<br>2: with circular or subpolygonal pits	
52.	Sculpturing on postorbital and squamosal when parietal and frontal are ornamented (*inapplicable in taxa lacking ornamentation on frontal and parietal*):<br>0: absent:<br>1: present – ornamentation on postorbital and squamosal	Wilberg 2015a (373)
53.	Dorsally flat skull table:<br>0: absent – supratemporal fenestrae and fossae cover most of surface of skull roof (surrounded by narrow ridges with no extended flat surface)<br>1: present – postorbital and squamosal with flat shelves extending laterally beyond quadrate contact (regardless of fenestra size)	Clark 1994 (24)
54.	Supratemporal fenestra:<br>0: present<br>1: reduced to a thin slit or absent as in *Gobiosuchus*	Ortega et al. 2000 (72)
55.	Supratemporal fenestra size relative to orbit:<br>0: smaller or nearly same size as orbit<br>1: larger than orbit, but less than twice as long as wide<br>2: larger than orbit, but nearly twice as long as wide	Wu et al. 2001 (131)
56.	Cranial table width relative to ventral portion of skull:<br>0: nearly as wide as ventral portion of skull<br>1: narrower than ventral portion	Wu et al. 2001 (123)
57.	Wide frontal plate in the anteromedial corner of the supratemporal fossa ("intratemporal flange" sensu Young et al. 2012):<br>0: absent<br>1: present	Jouve 2009 (320)
58.	Supratemporal fossa, anterior margin in dorsal view (ORDERED):<br>0: anterior margin posterior to the postorbital<br>1: anterior margin reaches between the anterior and posterior points of the frontal-postorbital suture<br>2: reaches at least as far anteriorly as the postorbital<br>3: projects further anteriorly than the postorbital and reaches the interorbital minimum distance	Young et al. 2012 (38)

59.	Supratemporal fossae, shape, anteroposterior and lateromedial axes (ORDERED):<br>0: longitudinal ellipsoid/sub-rectangular (anteroposterior axis more than 10% longer than the lateromedial axis)<br>1: sub-square/sub-circular (anteroposterior and lateromedial axes subequal, ± 5%)<br>2: transverse ellipsoid/sub-rectangular (lateromedial axis more than 10% longer than the anteroposterior axis)	Young et al. 2012 (39)
60.	Supratemporal fossae, shape, parallelogram (lateral and medial margins, and anterior and posterior margins are sub-parallel – anterior and posterior margins swept back, being directed slightly posterolaterally):<br>0: no<br>1: yes as in *Machimosaurus buffetauti, Steneosaurus leedsi*	Young et al. 2012 (41)
61.	Supratemporal fossa, in dorsal view, posterior limit (ORDERED):<br>0: terminates well before the posterior-most point of the parietal<br>1: either terminates near the posterior-most of the parietal or exceeds it, but never reaches the supraoccipital<br>2: more posterior than intertemporal bar	Young et al. 2012 (43)
62.	Posterior edge of the supratemporal fenestra:<br>0: thin (with fossa extending to posterior limit – thin ridge)<br>1: thin, but not a narrow ridge (no posterior extension of fossa)<br>2: thick	Jouve 2004 (184)
63.	Frontal contribution to supratemporal fossa:<br>0: excluded or nearly excluded from supratemporal fossa<br>1: extends well into supratemporal fossa	Wu et al. 2001 (23)
64.	Angle between posteromedial process (interfenestral bar) and lateral process of frontal (posterodorsal margin of orbit) in dorsal view (ORDERED):<br>0: nearly 90° or obtuse<br>1: less than 90° - approximately 70°-60°<br>2: much less than 90° - approximately 45°	Jouve 2009 (267)
65.	Frontal-postorbital suture on dorsal surface of skull (anterior to the supratemporal fenestra):<br>0: straight or irregular<br>1: V-shaped, frontal tapers laterally, sending a lateral process within the postorbital on dorsal surface of skull	Jouve 2009 (268)
66.	Anterolaterally directed ridges on frontal following postorbital/frontal suture, joining with midline frontal ridge posteriorly forming posteriorly pointing arrow shape:	Wilberg 2015b (66)

	0: absent<br>1: present as in *Pissarachampsa*	
67.	Parietal portion of intertemporal bar:<br>0: broad region separating supratemporal fossae (sculptured if sculpturing present on skull)<br>1: narrow – elevated sagittal crest present<br>2: narrow – flat, T-shaped in cross-section as in *Dyrosaurus maghribensis*	Clark 1994 (33)
68.	Sagittal crest shape (*inapplicable in taxa lacking a sagittal crest*):<br>0: narrow, but similar in height along entire length and dorsally flat as in *Steneosaurus bollensis*<br>1: narrow, but of uniform width with distinct medial groove as in *Metriorhynchus superciliosus*<br>2: narrows abruptly posteriorly at frontal parietal suture and is dorsoventrally expanded as in *Suchodus brachyrhynchus*<br>3: broadens posteriorly – parietal portion is broader than frontal portion as in *Dakosaurus andiniensis*	Wilberg 2015a (374)
69.	Width of anterior (frontal) and posterior (parietal) portions of interfenestral bar (*inapplicable in taxa lacking frontal contribution to interfenestral bar*):<br>0: uniform – anterior and posterior portions approximately same width<br>1: anterior portion (frontal) much wider than posterior portion (parietal)	Jouve 2009 (266)
70.	Parieto-postorbital suture on dorsal skull roof:<br>0: absent from skull roof and supratemporal fossa<br>1: absent from dorsal surface of skull roof, but broadly present within supratemporal fossa<br>2: present on dorsal surface of skull roof and within supratemporal fossa	Clark 1994 (23)
71.	Anterior process of parietal wedging between frontal and laterosphenoid in supratemporal fossa:<br>0: absent<br>1: Thin process present - participates to the anteroventral margin of the supratemporal fossa, below the frontal within the fenestra	Jouve 2009 (270)
72.	Posterior margin of the parietal in dorsal view:<br>0: relatively straight or gently concave	Young et al. 2012 (68)

	1: with pronounced, deep concavity opening posteriorly as in *Cricosaurus elegans* (*similar morphology also present in some dyrosaurs – e.g.* Rhabdognathus keinensis *and mahajangasuchids*)<br>2: posteriorly expanded (convex) as in *Oceanosuchus boecensis*<br>3: broad v-shape as in *Hyposaurus rogersii*	
73.	Supraoccipital exposure on cranial roof:<br>0: absent – parietals contact on occiput preventing dorsal exposure of supraoccipital<br>1: present – clearly exposed on dorsal surface of cranial roof	Ortega et al. 2000 (62)
74.	Anterior opening of temporo-orbital (temporal) canal in dorsal view:<br>0: exposed<br>1: hidden by overlapping squamosal rim of supratemporal fossa	Ortega et al. 2000 (75)
75.	Relative length between postorbital and squamosal:<br>0: squamosal is longer<br>1: postorbital is longer	Ortega et al. 2000 (33)
76.	Dorsal part of the postorbital:<br>0: with anterior and lateral edges only<br>1: with anterolaterally facing edge so that skull roof and supratemporal fenestrae narrow anteriorly – not for articulation with posterior palpebral<br>2: with anterolaterally facing edge for articulation with palpebral as in *Notosuchus terrestris*	Clark 1994 (29)
77.	Posterolateral projections of squamosal (squamosal prongs):<br>0: absent - posterior edge of squamosal nearly flat<br>1: present - posterolateral edge of squamosal extending posteriorly as an elongate process	Clark 1994 (36)
78.	Morphology of squamosal prong (*inapplicable in taxa lacking a squamosal prong*):<br>0: short, poorly developed, at same level as skull table<br>1: elongated, thin, and posteriorly directed (not ventrally deflected) as in *Rhabdognathus aslerensis*<br>2: elongated, posterolaterally directed, and ventrally deflected as in *Notosuchus terrestris*<br>3: elongated and ventrally directed, forming a near 90 degree	Riff 2007 (36)

	angle with skull table<br>4: elaborate squamosal horns projecting dorsally as in *Kaprosuchus*	
79.	Descending process of squamosal anterior to quadrate:<br>0: present<br>1: absent	Clark et al. 2000 (11)
80.	Posterior extent of squamosal relative to quadrate condyle in lateral view:<br>0: squamosal terminates anterior to the quadrate condyle<br>1: reaches quadrate condyle (or nearly so)<br>2: extends far posterior to the quadrate condyle	Jouve 2004 (90)
81.	Three curved ridges (oriented longitudinally) on dorsal surface of posterolateral region of squamosal:<br>0: absent<br>1: present as in *Zaraasuchus shepardi*	Pol and Norell 2004b (184)
82.	Posterolaterally directed facet on posterolateral margin of squamosal:<br>0: absent<br>1: present as in *Metriorhynchus superciliosus*	Wilberg 2015a (359)
83.	Dorsoventral height of squamosal portion of lateral rim of supratemporal fenestra with respect to interefenstral bar (ORDERED):<br>0: at same level (flat skull table)<br>1: slightly deflected ventrally as in *Steneosaurus bollensis*<br>2: strongly deflected ventrally as in *Cricosaurus suevicus*	Wilberg 2015a (334)
84.	Squamosal overhang of lateral temporal region:<br>0: not significant – weakly developed<br>1: broad lateral expansion overhanging lateral temporal region	Clark et al. 2004 (10)
85.	Squamosal ridge on dorsal surface along edge of supratemporal fossa:<br>0: absent<br>1: present as in *Sphenosuchus acutus*	Clark et al. 2004 (12)

Orbit and temporal region

86.	Orbit orientation:<br>0: more circular in lateral aspect<br>1: more circular in dorsal aspect	Jouve 2009 (310)
87.	Sclerotic ossicles:<br>0: absent<br>1: present	Young and Andrade 2009 (19)
88.	Lateral border of orbit relative to lateral border of	Wu et al. 2001

	supratemporal fossa:<br>0: lateral to<br>1: medial to	(130)
89.	Prefrontal and lacrimal around orbits:<br>0: forming flat rims – flush with external surface of skull<br>1: evaginated – forming elevated rims as in *Gavialis gangeticus*	Gasparini et al. 2006 (256)
90.	Descending process of prefrontal ("prefrontal pillar"):<br>0: absent<br>1: present	Clark et al. 2004 (5)
91.	Descending process of prefrontal (prefrontal pillar) integration in palate (*inapplicable in taxa lacking a descending process of prefrontal*):<br>0: does not reach palate<br>1: reaches palate and solid integrated	Clark 1994 (15)
92.	Prefrontal pillars when integrated in palate (*inapplicable in taxa lacking a descending process of prefrontal or lacking contact between prefrontal pillar and palate*):<br>0: pillars transversely expanded<br>1: pillars transversely expanded in their dorsal half and columnar ventrally<br>2: pillars longitudinally expanded in their dorsal part and columnar ventrally	Ortega et al. 2000 (54)
93.	Lacrimal orbital contour:<br>0: facing laterally<br>1: facing laterodorsally	Ortega et al. 2000 (172)
94.	Lacrimal in dorsal view:<br>0: visible<br>1: not visible	Jouve 2009 (315)
95.	Ventral portion of the lacrimal – contact with jugal:<br>0: extending ventroposteriorly widely contacting the jugal<br>1: tapering ventroposteriorly, does not contact or contacts the jugal only slightly as in *Mariliasuchus amarali*	Pol and Apesteguía 2005 (224)
96.	Anterior extension of the jugal relative to the anterior margin of the orbit:<br>0: does not exceed the anterior margin of the orbit<br>1: exceeds the anterior margin but length (measured from anterior margin of orbit to anterior tip of jugal) is less than that the orbital length<br>2: greatly exceeds anterior margin such that the anterior process of jugal is as long or longer than the orbital length	Pol 1999 (134)

97.	Dorsoventral height of antorbital region of the jugal with respect to infraorbital region (*inapplicable in taxa lacking a jugal exceeding the anterior orbital margin*):<br>0: equal or narrower<br>1: antorbital region greatly expanded (150% or more than minimal height of the jugal below the orbit) as in *Sphagesaurus huenei*	Pol and Norell 2004 (130)
98.	Lateral surface of anterior process of jugal:<br>0: flat or convex<br>1: with broad shelf ventral to the orbit with triangular depression beneath it as in *Sphagesaurus huenei*	Pol 1999 (133)
99.	Ventral margin of jugal between ventral contact with maxilla and quadratojugal in lateral view:<br>0: relatively straight<br>1: arched dorsally as in *Simosuchus clarki*	Pol and Norell, 2004 (179)
100.	Longitudinal ridge on lateral surface of jugal below infratemporal fenestra:<br>0: absent<br>1: present as in *Zaraasuchus shepardi*	Pol and Norell 2004 (183)
101.	Elongate neurovascular groove on lateral surface of jugal beneath orbit:<br>0: absent<br>1: present as in *Steneosaurus brevior*	Wilberg 2015a (358)
102.	Jugal participation in ventral (or lateral) margin of orbit:<br>0: jugal broadly participates in the orbital margin<br>1: jugal excluded or nearly excluded from the orbit by lacrimal-postorbital contact as in *Platysuchus multiscrobiculatus*	Young et al. 2012 (75)
103.	Prefrontal–maxilla contact in the inner anteromedial region of orbit:<br>0: absent<br>1: present as in *Sphagesaurus*	Pol 1999 (162)
104.	Lateral margin of prefrontal relative to dorsal margin of the orbit:<br>0: continuous with, not laterally expanded<br>1: laterally expanded, forming a “prefrontal overhang” over the orbit as in *Metriorhynchus superciliosus*	Jouve 2009 (255)
105.	Prefrontal “overhang” over orbit (*inapplicable in taxa lacking a prefrontal overhang*):<br>0: very slight – approximately 5-10% of its width	Young and Andrade 2009 (12)

	1: greatly enlarged > 10%	
106.	Shape of lateral margin of prefrontal overhang in dorsal view (*inapplicable in taxa lacking a prefrontal overhang*):<br>0: gently curved (obtuse angle) with posterior margin anterolaterally directed<br>1: gently curved with posterior margin directed laterally nearly perpendicular to sagittal plane as in *Enaliosuchus macrospondylus*<br>2: with distinct point formed with posterior margin directed anterolaterally as in *Metriorhynchus casamiquelai*<br>3: with distinct point and posterior margin directed laterally, nearly perpendicular to sagittal plane as in *Dakosaurus andiniensis*	Young and Andrade 2009 (14)
107.	Lateral extent of prefrontal overhang relative to the posterolateral corner of the supratemporal fossa in dorsal view (*inapplicable in taxa lacking a prefrontal overhang*):<br>0: Prefrontal does reach the same plane laterally as the posterolateral corner of the supratemporal fossa<br>1: Prefrontal reaches or exceeds laterally than the posterolateral corner of the supratemporal fossa	Young et al. 2012 (48)
108.	Prefrontal/lacrimal suture raised, forming an anteroposteriorly directed ridge:<br>0: absent<br>1: present	Young and Andrade 2009 (150)
109.	Elements contributing to medial margin of the orbit:<br>0: primarily frontal<br>1: prefrontal contributes 50% or greater, reducing frontal contribution	Jouve 2009 (326)
110.	Maximum frontal interorbital width (not including prefrontals or fused palpebrals) –<br>0: relatively narrow<br>1: Greater than 50% of skull width at orbit	Clark, 1994 (20)
111.	Interfrontal suture at maturity:<br>0: remains open (frontals paired)<br>1: closed (frontals fused into a single element)	Clark 1994 (21)
112.	Narrow midline ridge on dorsal surface of frontal:<br>0: absent<br>1: present as in *Dibothrosuchus*	Wu et al. 2001 (23)
113.	Anterior process of the frontal relative to anterior margin of orbit:<br>0: extending far anterior<br>1: slightly anterior, subequal, or posterior	Jouve 2004 (178)

114.	Transverse frontal ridge (transverse interorbital crest):<br>0: absent<br>1: present as in *Eutretauranosuchus delfsi*	Jouve 2009 (319)
115.	Postfrontal:<br>0: present<br>1: absent	Clark et al. 2004 (8)
116.	Palpebral elements:<br>0: palpebrals present<br>1: palpebrals absent	Clark 1994 (65)
117.	Number of palpebral elements (*inapplicable in taxa lacking palpebrals*):<br>0: One small palpebral<br>1: two large palpebrals<br>2: one large palpebral	Clark 1994 (65)
118.	Palpebral contact with frontal (*inapplicable in taxa lacking palpebrals*):<br>0: separated from the lateral edge of the frontals<br>1: extensively sutured to each other and to the lateral margin of the frontals	Pol and Norell 2004 (181)
119.	Postorbital orientation relative to jugal on postorbital bar:<br>0: anterior to jugal<br>1: medial or posterior to jugal<br>2: lateral to jugal	Clark 1994 (16)
120.	Postorbital bar sculpturing (when sculpture present on skull - *inapplicable in taxa in which postorbital not medial to jugal*):<br>0: present<br>1: absent	Clark 1994 (25)
121.	Postorbital bar shape (*inapplicable in taxa in which postorbital not medial to jugal*):<br>0: transversely flattened<br>1: columnar	Clark 1994 (26)
122.	Vascular opening on lateral surface of the postorbital in the dorsal portion of postorbital bar:<br>0: absent<br>1: present	Clark 1994 (27)
123.	Ventral portion of postorbital bar:<br>0: flush with lateral surface of jugal<br>1: anteriorly continuous but posteriorly inset<br>2: medially displaced anteriorly and posteriorly – a ridge separates postorbital bar from lateral surface of jugal	Ortega et al. 2000 (34)
124.	Orientation of the base of the postorbital bar:<br>0: directed posterodorsally<br>1: directed dorsally	Pol 1999 (156)

	2: directed anterodorsally	
125.	Dorsal portion of postorbital bar:<br>0: inset from the dorsolateral margin of the postorbital<br>1: inset posteriorly but continuous anteriorly as in *Dakosaurus andiniensis*<br>2: lateral surface of postorbital continuous with postorbital bar (postorbital bar not inset)	Clark 1994 (30)
126.	Postorbital bar orientation in dorsal view:<br>0: ventrolaterally oriented – visible in dorsal view<br>1: vertical – not visible in dorsal view	Jouve 2004 (192)
127.	External surface of ascending process of jugal (*inapplicable in taxa with postorbital forming external surface of postorbital bar as in* Teleosaurus cadomensis):<br>0: exposed laterally<br>1: exposed posterolaterally as in *Gobiosuchus*	Pol and Norell 2004 (182)
128.	Postorbital participation to posterodorsal (in lateral view) or posteromedial (in dorsal view) margin of orbit:<br>0: postorbital is excluded from the orbit posterodorsal margin<br>1: postorbital reaches the orbit posterodorsal margin	Young et al. 2012 (72)
129.	Postorbital participation in posteroventral (in lateral view) or posterolateral (in dorsal view) margin of the orbit:<br>0: postorbital does not contribute to the posteroventral orbital margin<br>1: postorbital reaches the orbit posteroventral margin, forming part of the ventral margin of the orbit	Young et al. 2012 (74)
130.	Anterolateral process of the postorbital (ORDERED):<br>0: absent<br>1: small<br>2: extensive, contacting or nearly contacting the dorsal margin of the jugal as in *Rhabdognathus aslerensis*	Jouve 2004 (9)
131.	Ectopterygoid–postorbital contact: (*this character is inapplicable for thalattosuchians – the position of the postorbital on the lateral surface of the postorbital bar precludes contact with the ectopterygoid on the medial surface and is thus not homologous with a lack of contact when postorbital forms the medial surface*)<br>0: absent - ectopterygoid does not contact postorbital<br>1: ectopterygoid contacts postorbital on medial side of	Ortega et al. 2000 (36)

	postorbital bar	
132.	Infratemporal fenestra length:<br>0: anteroposteriorly shorter than dorsoventral height or subequal<br>1: elongated, approximately twice as long as deep	Ortega et al. 2000 (74)
133.	Infratemporal fenestra orientation:<br>0: facing laterally<br>1: facing dorsolaterally	Ortega et al. 2000 (46)
134.	Postorbital contribution to infratemporal fenestra border:<br>0: almost or entirely excluded (as in *Protosuchus richardsoni*)<br>1: bordering infratemporal fenestra	Wu et al. 1997 (108)
135.	Extent of postorbital contribution to dorsal margin of infratemporal fenestra (*inapplicable in taxa where postorbital is excluded from infratemporal fenestra*):<br>0: slight contribution (forms <50% of the dorsal border)<br>1: large contribution (forms >50% of the dorsal border)	Jouve 2004 (59)
136.	Jugal shape ventral to infratemporal fenestra:<br>0: transversely flattened<br>1: rod-like	Clark 1994 (18)
137.	Length of posterior process of jugal relative to anterior process:<br>0: equal in length or longer<br>1: shorter, but greater than 50% of the length of the anterior process<br>2: much shorter, less than 50% of the length of the anterior process	Wu et al. 2001 (102)
138.	Posterior limit of posterior process of the jugal relative to infratemporal fenestra:<br>0: exceeds the posterior border of the infratemporal fenestrae<br>1: terminates anterior to or reaches posterior border of the infratemporal fenestra	Pol 1999 (150)
139.	Infratemporal fenestra length relative to supratemporal fenestra:<br>0: much shorter than supratemporal fenestra<br>1: subequal<br>2: longer than supratemporal fenestra	Jouve 2009 (241)
140.	Quadratojugal dorsal process contact with postorbital:<br>0: absent<br>1: present	Ortega et al. 2000 (49)
141.	Quadratojugal dorsal process contact with postorbital (*inapplicable in taxa lacking quadratojugal/postorbital contact*):<br>0: narrow, contacting only small part of postorbital<br>1: broad, extensively contacting postorbital and greatly reducing size of infratemporal fenestra as in *Gobiosuchus*	Clark 1994 (19)

142.	Jugal-quadratojugal suture relative to posterior corner of the infratemporal fenestra in lateral view:<br>0: jugal–quadratojugal suture lies at posteroventral corner<br>1: quadratojugal extends anteriorly forming part of ventral edge of infratemporal bar	Ortega et al. 2000 (39)
143.	Posteroventral extent of quadratojugal:<br>0: reaches the quadrate condyle<br>1: terminates prior to reaching the quadrate condyle	Pol 1999 (155)
144.	Posterolateral end of quadratojugal:<br>0: acute or rounded, tightly overlapping the quadrate<br>1: with sinusoidal ventral edge and wide and rounded posterior edge slightly overhanging the lateral surface of the quadrate as in *Zosuchus, Sichuanosuchus*	Pol and Norell 2004 (180)
145.	Quadratojugal spine at posterior margin of infratemporal fenestrae:<br>0: absent<br>1: present	Ortega et al. 2000 (47)
146.	Dorsal and ventral rims of squamosal groove for external ear-flap musculature:<br>0: absent<br>1: ventral placed lateral to dorsal<br>2: ventral directly beneath dorsal	Young and Andrade 2009 (112)
147.	Subtriangular concavity located on the posterolateral surface of the squamosal, located posteriorly to the otic shelf recess and anterolaterally from the paroccipital process:<br>0: absent<br>1: present as in *Almadasuchus figarii*	Pol et al. 2013 (75)2

Palate and perichoanal structures

148.	Palatal part of premaxillae:<br>0: not in contact posterior to incisive foramen<br>1: in contact posteriorly along contact with maxillae<br>2: in contact along entire length due to lack of incisive foramen as in *Gobiosuchus*	Wu et al. 2001 (7)
149.	Incisive foramen position (*inapplicable in taxa lacking an incisive foramen*):<br>0: completely separated from premaxillary tooth row, at the level of the second or third alveolus<br>1: abuts premaxillary tooth row	Brochu 1997 (153)
150.	Palatal branches of maxillae (ORDERED):<br>0: not in contact in palate<br>1: posterior portion not in contact on palate at sutures with	Clark 1994 (10)

	palatines<br>2: in contact for entire length	
151.	Paired foramina on palatal surface of the premaxilla-maxilla suture (not pits for dentary teeth):<br>0: absent<br>1: present as in *Simosuchus clarki*	Jouve 2009 (248)
152.	Ornamentation of palatal surface of maxilla:<br>0: maxillary palatal surface smooth<br>1: maxillary palatal surface ornamented with ridges as in *Protosuchus richardsoni*<br>2: maxillary palatal surface ornamented with pits as in *Kayentasuchus*	Ortega et al. 2000 (2)
153.	Palatine contribution to palate (ORDERED):<br>0: palatal processes of palatines weakly developed - not in contact on palate below narial passage<br>1: palatal processes well developed, forming palatal shelves that do not meet<br>2: palatines in contact ventral to narial passage, forming part of secondary palate	Clark 1994 (37)
154.	Ornamentation of palatal surface of palatines:<br>0: absent – smooth<br>1: present – pitted as in *Fruitachampsa callisoni*	Wilberg 2015a (340)
155.	Palatine anteromedial process anterior extent (*inapplicable in taxa lacking midline contact between maxilla and palatine*):<br>0: exceeds the anterior margin of the suborbital fenestrae, extending anteriorly between the maxillae<br>1: terminates posterior to the anterior margin of the suborbital fenestrae as in *Stratiotosuchus*	Pol 1999 (143)
156.	Shape of anterior process of palatine (maxilla-palatine suture) near midline on palatal surface (*inapplicable in taxa lacking midline contact between maxilla and palatine*):<br>0: palatine tapers anteriorly (rounded or pointed)<br>1: palatine anteromedially straight, perpendicular to the longitudinal axis of the skull<br>2: palatine invaginated	Brochu 1999 (108)
157.	Palatine, anterior margin has two distinct non-midline anterior processes (*inapplicable in taxa lacking midline contact between maxilla and palatine*):<br>0: absent<br>1: present as in *Pelagosaurus typus*	Young et al. 2012 (93)
158.	Palatomaxillary foramina (small anteriorly directed foramina on either side of midline at palatine/maxilla suture, opening into a ventral maxillary groove:	Jouve 2004 (104)

	0: absent<br>1: present as in *Pelagosaurus typus*	
159.	Palatomaxillary groove extent (*inapplicable in taxa lacking palatomaxillary foramina*)<br>0: restricted to maxillae<br>1: extend anteriorly onto maxillae and caudally onto surface of palatines	Wilberg 2015a (339)
160.	Maxillo-palatal fenestrae (moderately sized fenestrae opening ventrally from maxilla/palatine suture on palate, connecting nasal cavity with oral cavity):<br>0: absent<br>1: present as in *Notosuchus terrestris*	Wu et al. 2001 (128)
161.	Foramina on palatine ventral surface:<br>0: absent<br>1: present – foramina lying along grooves on either side of midline as in *Pissarachampsa*	Wilberg 2015b (159)
162.	Orientation of posterior region of palatines (*inapplicable in taxa lacking midline contact between palatines*):<br>0: run parasagittaly along midline<br>1: palatines diverge posterolaterally becoming rod-like caudally forming palatine bars as in *Notosuchus terrestris*	Martinelli 2003 (36)
163.	Choanal opening:<br>0: continuous with pterygoid ventral surface except for anterior and anterolateral borders<br>1: opens into palate through a deep midline depression (choanal groove)	Clark 1994 (39)
164.	Choanae size:<br>0: of moderate size<br>1: extremely large, nearly half of maximal skull width as in *Notosuchus terrestris*<br>2: very narrow, elongate, more than three times longer than wide as in *Eutretauranosuchus*	Clark 1994 (42)
165.	Elements contributing to anterior margin of choanae (ORDERED):<br>0: vomers and maxillae<br>1: maxillae only<br>2: palatines only<br>3: pterygoid with small participation of palatine<br>4: Completely enclosed within pterygoids	Wu et al. 2001 (44)
166.	Palatine portion of anterior margin of the choanal opening	Jouve 2009

	(*inapplicable in taxa lacking a palatine contribution to anterior border*):<br>0: gently rounded<br>1: tapers anteriorly between the palatines as in *Pelagosaurus typus*<br>2: w-shaped – with short midline posterior process of palatines forming middle portion of "w" as in *Metriorhynchus leedsi*<br>3: with short posterior processes (rugosities) on either side of midline as in *Machimosaurus hugii*	(242)
167.	Internal choanal opening:<br>0: opening posteriorly and continuous with pterygoid surface<br>1: closed posteriorly by an elevated wall formed by the pterygoids	Pol and Norell 2004a (183)
168.	Internal choanal groove:<br>0: undivided<br>1: partially septated<br>2: or completely septated	Clark 1994 (69)
169.	Internal choanal septum shape (*inapplicable in taxa lacking a choanal septum*):<br>0: narrow vertical bony sheet<br>1: T-shaped bar expanded ventrally	Pol and Apesteguía 2005 (186)
170.	Flat ventral surface of internal choanal septum (*inapplicable in taxa lacking a t-shaped choanal septum*):<br>0: uniform width (parallel sided)<br>1: tapering anteriorly as in *Araripesuchus gomesii*<br>2: expanding anteriorly as in *Mahajangasuchus insignis*	Pol and Apesteguía 2005 (220)
171.	Anteroposterior position of posterior margin of choana relative to posterior edge of suborbital fenestra:<br>0: anterior to<br>1: posterior to	Wu et al. 2001 (143)
172.	Anteroposterior position of the anterior margin of choanae relative to suborbital fenestra:<br>0: anterior to the posterior margin of suborbital fenestra<br>1: posterior to posterior margin of suborbital fenestra	Jouve 2004 (23)
173.	Palatine contribution to suborbital fenestra border (*inapplicable in taxa lacking a suborbital fenestra*):<br>0: participates in suborbital fenestra<br>1: entirely excluded from suborbital fenestra	Wu et al. 2001 (109)
174.	Anterior extent of suborbital fenestrae relative to anterior border of orbit:<br>0: end anteriorly at level of anterior border of orbit<br>1: extend further anteriorly	Jouve 2004 (195)

	2: terminate posterior to the anterior border of the orbit	
175.	Suborbital fenestra:<br>0: large opening<br>1: extremely reduced or absent as in *Hsisosuchus*	NEW
176.	Vomer contribution to secondary palate (does not include palatal exposure due to lack of palatine or maxillary palatal processes meeting at midline):<br>0: vomer contributes flattened plate to secondary palate as in *Simosuchus clarki*<br>1: vomer forms no contribution to secondary palate	Buckley et al. 2000 (115)
177.	Edentulous portion of posterior process of ventral lamina of maxilla:<br>0: short<br>1: long (room for at least 2 additional posterior maxillary teeth)	Jouve 2009 (250)
178.	Ectopterygoid–maxilla contact:<br>0: absent – ectopterygoid does not contact palatal branch of maxilla<br>1: present	Ortega et al. 2000 (61)
179.	Ectopterygoid contact with palatal branch of maxilla (*inapplicable in taxa lacking ectopterygoid-maxilla contact*):<br>0: very slight contact<br>1: extensive contact – suture mediolaterally oriented (perpendicular to sagittal plane)<br>2: extensive contact – suture primarily oriented anteromedially<br>3: contact – suture oriented anterolaterally as in *Teleosaurus cadomensis*	Jouve 2009 (279)
180.	Ectopterygoid medial process:<br>0: single<br>1: forked	Ortega et al. 2000 (146)
181.	Ectopterygoid pneumaticity:<br>0: absent<br>1: present (ectopterygoid foramen/foramina) as in *Pissarrachampsa*	Jouve 2009 (324)
182.	Ectopterygoid projecting medially on ventral surface of pterygoid flanges:<br>0: barely extended<br>1: widely extended covering approximately the lateral half of the ventral surface of the pterygoid flanges as in *Notosuchus terrestris*, *Dyrosaurus phosphaticus*	Pol and Apesteguía 2005 (230)
183.	Posterior process of ectopterygoid projecting along ventral surface of jugal:	Pol 1999 (148)

	0: developed<br>1: absent as in *Sphagesaurus huenei*	
184.	Pterygoid:<br>0: restricted to palate and suspensorium, joints with quadrate and basisphenoid overlapping<br>1: pterygoid extending dorsally to contact laterosphenoid and forming ventrolateral edge of trigeminal foramen, strongly sutured to quadrate and laterosphenoid	Clark 1994 (38)
185.	Interpterygoid suture at maturity:<br>0: present - visible posterior to choana<br>1: closed – pterygoids fused into single element	Clark 1994 (41)
186.	Sculpturing of palatal surface of pterygoid:<br>0: absent<br>1: present as in *Protosuchus richardsoni*	Clark 1994 (40)
187.	Pterygoids between basisphenoid and choana:<br>0: separated - not in contact along midline on palatal surface as in *Postosuchus kirkpatricki*<br>1: in contact along midline	Wu et al. 2001 (121)
188.	Pterygoid pneumatisation:<br>0: absent - pterygoid thin, sheet-like<br>1: present as in *Gobiosuchus*	Wu et al. 2001 (106)
189.	Posterior extent of posteromedial process of pterygoid relative to medial Eustachian foramen:<br>0: terminates anterior to the level of the medial eustachian foramen<br>1: reaches same level as the medial eustachian foramen	Jouve 2004 (114)
190.	Anteroposterior position of posterolateral margin of the pterygoid (torus transiliens) relative to medial Eustachian foramen in ventral view:<br>0: terminates far anterior to the medial eustachian foramen<br>1: reaches approximately the same anteroposterior level as the medial eustachian foramen<br>2: terminates far posterior to the medial eustachian foramen	Jouve 2004 (115)
191.	Shape of quadrate ramus of pterygoid in ventral view:<br>0: narrow and elongate<br>1: broad in ventral view<br>2: narrow and very short in ventral view	Wu et al. 2001 (119)
192.	In ventral view, narrow flanges of pterygoid extend posterolaterally along lateral margin of basisphenoid forming small posterolateral pterygoid-basisphenoid “wings” separating basisphenoid from contact with quadrate laterally:	Jouve 2004 (117)

	0: absent<br>1: present as in *Steneosaurus durobrivensis*	
193.	Basisphenoid exposure on lateral surface of braincase:<br>0: absent, basisphenoid not visible on lateral surface<br>1: present as in *Alligator mississippiensis*	Pol 1999 (163)
194.	Palatine-pterygoid contact on palate:<br>0: palatines overlie pterygoids as in *Protosuchus richardsoni*<br>1: palatines firmly sutured to pterygoids	Pol and Norell 2004a (165)
195.	Pterygoid participation in posterior border of suborbital fenestrae:<br>0: present<br>1: absent – excluded by posterolateral processes of palatines or medial expansion of ectopterygoids	Wilberg 2015a (324)
196.	Depressions on the ventral surface of the pterygoid within the internal choana (parachoanal fossae Pinheiro et al. 2008, or pterygoid depresssions; Andrade et al. 2006 – *inapplicable in taxa lacking pterygoid contribution to internal choana*):<br>0: absent<br>1: present	Wilberg 2015a (384)
197.	Anterior process of pterygoid ramus of quadrate contact with pterygoid:<br>0: not sutured<br>1: firmly sutured	Jouve 2009 (280)
198.	Infratemporal fenestra in ventral view:<br>0: largely hidden by the pterygoid flange<br>1: largely visible lateral to the pterygoid flange	Jouve 2004 (189)
199.	Anterior extent of ventral lamina of jugal relative to ectopterygoid:<br>0: extends anterior to the ectopterygoid<br>1: terminates anteriorly at the level of the ectopterygoid or posterior to	Jouve 2004 (68)

Occiptial

200.	Parietal contribution to occipital surface:<br>0: broad occipital portion<br>1: parietal narrows posteriorly, resulting in minor contribution as in *Gobiosuchus*	Clark 1994 (32)
201.	Parietal width on occipital surface relative to supraoccipital width:<br>0: widely exposed – much wider than the supraoccipital	Jouve 2009 (321)

	1: minor occipital portion – similar in size to supraoccipital	
202.	Posterodorsal margin of the skull roof in occipital view:<br>0: relatively flat surface or gently convex<br>1: sigmoidal, strongly W-shaped – dorsal margin of the supraoccipital is much higher than the dorsal margin of the squamosal as in *Metriorhynchus superciliosus*<br>2: V-shaped – dorsal margin of supraoccipital at the midline lies ventral to dorsal margin of squamosal as in *Mahajangasuchus insignis*<br>3: strongly convex as in *Dyrosaurus phosphaticus*	Jouve 2009 (323)
203.	Post-temporal fenestra size and elements contributing to fenestral margin (ORDERED):<br>0: large and enclosed by parietal, squamosal, and exoccipital, well separated from the supraoccipital as in *Dibothrosuchus elaphros*<br>1: large and enclosed by the squamosal and exoccipital, with its medial end located close to the lateral edge of the supraoccipital as in *Almadasuchus figarii*<br>2: small and with supraoccipital participating from its medial margin as in *Protosuchus richardsoni*	Pol et al. 2013 (74)
204.	Supraoccipital contribution to foramen magnum (ORDERED):<br>0: forms part of dorsal margin of foramen magnum, exoccipitals broadly separated<br>1: forms small part of dorsal margin of foramen magnum – exoccipitals approach midline but do not contact<br>2: exoccipitals in contact dorsal to foramen magnum, separating supraoccipital from foramen	Clark et al. 2003 (20)
205.	Supraoccipital shape:<br>0: more or less triangular<br>1: pentagonal as in *Sphenosuchus acutus*	Wu et al. 2001 (117)
206.	Mastoid antrum:<br>0: does not extend through supraoccipital<br>1: extending through transverse canal in supraoccipital to connect middle ear regions	Clark 1994 (63)
207.	Paroccipital process contact with squamosal:<br>0: in loose contact with squamosal laterally<br>1: paroccipital process laterally narrow and sutured to squamosal<br>2: paroccipital process very deep dorsoventrally, interlocked with squamosal as in *Dibothrosuchus elaphros*	Wu et al. 2001 (115)
208.	Unsculpted ventral projection of the squamosal enclosing the dorsal half of the paraoccipital process:	Jouve 2009 (99)

	0: absent<br>1: present as in *Rhabdoghathus aslerensis*	
209.	Bilateral posterior prominences ("occipital tuberosities") on posterior surface of exoccipitals (ORDERED):<br>0: absent – relatively flat<br>1: present, but small, as in *Chenanisuchus lateroculus*<br>2: strongly developed as in *Dyrosaurus phosphaticus*	Clark 1994 (64)
210.	Cranial nerves IX–XI foramina in otoccipital:<br>0: exiting through common large foramen vagi<br>1: cranial nerve IX exiting medial to nerves X and XI through a separate foramen	Clark 1994 (59)
211.	Foramen for the internal carotid artery (ORDERED – *inapplicable in taxa lacking a contact between quadrate and exoccipital to enclose internal carotid artery*):<br>0: small, similar in size to the openings for cranial nerves IX-XI<br>1: slightly enlarged, larger than other foramina on occipital surface as in *Pelagosaurus typus*<br>2: extremely enlarged, more than twice as large as other foramina as in *M. superciliosus*	Gasparini et al. 2006 (248)
212.	Ventrolateral extension of the exoccipital contacting the dorsal surface of the quadrate:<br>0: short<br>1: exoccipital broadly covers the dorsal surface of quadrate	Jouve 2009 (322)
213.	Medioventral projection of exoccipital in relation to ventral limit of basioccipital in occipital view:<br>0: terminates well dorsal<br>1: nearly reaches	Jouve 2009 (340)
214.	Paroccipital process, orientation in occipital view:<br>0: horizontal<br>1: dorsolaterally orientated, at a 45 degree angle as in *Cricosaurus suevicus*<br>2: ventral-edge horizontal, then terminal third sharply inclined dorsolaterally at a 45 degree angle as in *Dakosaurus andiniensis*<br>3: prominently arched ventrally as in *Dyrosaurus phosphaticus*	Young et al. 2012 (104)
215.	Exoccipital contribution to occipital condyle:<br>0: slight contribution<br>1: large contribution as in *Dyrosaurus phosphaticus*	Jouve 2004 (96)
216.	Orientation of occipital condyle:<br>0: posteriorly directed<br>1: posteroventrally directed as in *Notosuchus*	Ortega et al. 2000 (176)
217.	Ventral portion of basioccipital in occipital view:<br>0: thin, without well-developed bilateral tuberosities	Clark 1994 (57)

	1: ventral portion anteroposteriorly thick, rugous, with pendulous tubera formed primarily from basioccipital<br>2: pendulous tubera with large contribution from exoccipitals as in *Rhabdognathus aslerensis*	
218.	Basioccipital with laterally directed knobs:<br>0: absent or slightly developed<br>1: strongly developed as in *Lomasuchus palpebrosus*	Gasparini et al. 1991 (15)
219.	Ventral projection of the basioccipital in occipital view:<br>0: ventrally indistinct from exoccipital<br>1: distinct from exoccipital, ventrally offset	Jouve 2004 (142)
220.	Posterior surface of basioccipital ventral to the occipital condyle:<br>0: short and gently curved – dorsoventrally shorter than the occipital condyle<br>1: elongate, flat and nearly vertical – at least as high as occipital condyle	Jouve 2004 (197)

Braincase, basicranium and suspensorium

221.	Quadrate pneumaticity (ORDERED):<br>0: absent – quadrate lacks fenestra<br>1: present – single fenestra<br>2: present – three or more fenestrae present on dorsal and posteromedial surfaces	Clark 1994 (45)
222.	Dorsal primary head of quadrate contact with laterosphenoid:<br>0: not sutured with laterosphenoid<br>1: sutured with laterosphenoid	Clark 1994 (47)
223.	Contribution of squamosal to dorsal margin of the external otic aperture:<br>0: absent – dorsal margin formed by the quadrate, the squamosal does not participate<br>1: present – dorsal margin formed by squamosal	Jouve 2009 (131)
224.	Squamosal contact with the posterodorsal surface of the quadrate closing the otic recess posteriorly:<br>0: absent<br>1: present	Pol et al. 2013 (76)
225.	Anterior extension of the otic recess (ORDERED - *inapplicable in taxa lacking a squamosal contribution to external otic aperture*):<br>0: restricted to the squamosal<br>1: extends on the posterior region of the lateral surface of the postorbital<br>2: extends along the entire length of the postorbital, which has an anterior transverse lamina that separates the otic recess	Sereno and Larsson 2009 (69)

	from the orbit	
226.	Deep groove along ventral edge of pterygoid ramus of quadrate<br>0: absent – ventral edge flat<br>1: present as in *Protosuchus richardsoni*	Clark 1994 (50)
227.	Ventromedial part of quadrate contact with exoccipital on occiput:<br>0: absent<br>1: present – contacts exoccipital to enclose internal carotid artery and form passage for cranial nerves IX–XI	Clark 1994 (51)
228.	Quadrate contact with basisphenoid in ventral view:<br>0: absent<br>1: present	Wu et al. 2001 (104)
229.	Distal part of quadrate body:<br>0: distinct<br>1: indistinct due to ventromedial contact of quadrate body with otoccipital as in *Gobiosuchus*	Wu et al. 2001 (105)
230.	Ventral surface of quadrate:<br>0: slightly concave<br>1: strongly concave, with distinct, obliquely orientated crest as in *Dibothrosuchus*	Wu et al. 2001 (120)
231.	Mandibular condyle of quadrate dorsoventral position:<br>0: approximately level with occipital condyle<br>1: ventral to occipital condyle at approximately the same dorsoventral level as the lower tooth row<br>2: ventral to occipital condyle but below level of the lower tooth row	Wu and Sues 1996 (24)
232.	Quadrate condyles:<br>0: almost aligned<br>1: medial condyle expanded ventrally as in *Notosuchus terrestris*	Ortega et al. 2000 (53)
233.	Quadrate distal end:<br>0: with only one plane facing posteriorly<br>1: with two distinct faces in posterior view, a posterior one and a medial one bearing the foramen aereum	Pol 1999 (167)
234.	Quadrate major axis direction:<br>0: posteroventrally<br>1: ventrally or anteroventrally	Pol 1999 (166)
235.	Orientation of quadrate body distal to otoccipital-quadrate contact in posterior view:<br>0: ventrally<br>1: ventrolaterally	Pol and Norell 2004a (181)
236.	Cross section of distal end of quadrate:	Pol and Norell

	0: mediolaterally wide and anteroposteriorly thin<br>1: sub-quadrangular	2004a (164)
237.	Posterior edge of quadrate:<br>0: broad medial to tympanum, gently concave<br>1: posterior edge narrow dorsal to otoccipital contact, strongly concave	Clark 1994 (46)
238.	Ridge along dorsal section of quadrate-quadratojugal contact:<br>0: absent<br>1: present as in *Zaraasuchus shepardi*	Pol and Norell 2004b (185)
239.	Large depression on lateral surface of quadrate (quadrate depression) ventral to otic aperture:<br>0: absent<br>1: present as in *Striatosuchus*	Wilberg 2015b (236)
240.	Ventrolateral contact of otoccipital with quadrate:<br>0: very narrow as in *Protosuchus richardsoni*<br>1: broad	Clark 1994 (48)
241.	Cranio-quadrate canal (ORDERED):<br>0: laterally open<br>1: closed off by a thin lamina formed by squamosal, quadrate, and exoccipital – near lateral edge of skull<br>2: closed off by a thick lamina formed by squamosal, quadrate, and exoccipital	Clark 1994 (49)
242.	Dorsal lamina of exoccipital (anterior to the cranioquadrate canal) sutured to the quadrate or squamosal dorsally when the cranioquadrate canal is closed off anteriorly by a thin lamina (*inapplicable in taxa with an open cranioquadrate canal and taxa in which cranioquadrate canal is closed off by a thick lamina*):<br>0: absent - dorsal lamina of exoccipital is not sutured to the quadrate or squamosal dorsally<br>1: present - dorsal lamina of exoccipital is sutured to the quadrate or squamosal dorsally	Jouve 2009 (343)
243.	Anterior opening of cranio-quadrate passage in otic area (*inapplicable in taxa where cranio-quadrate canal is open laterally*):<br>0: not expanded (otic aperture oval in shape)<br>1: opening expanded forming a caudal notch as in *Crocodylus niloticus*	Ortega et al. 2000 (159)
244.	Laterally concave descending flange of otoccipital ventral to subcapsular process:	Clark 1994 (58)

	0: absent<br>1: present	
245.	Crista interfenestralis (between fenestra ovalis and fenestra pseudorotunda):<br>0: nearly vertical<br>1: horizontal	Clark 1994 (61)
246.	Lateral eustachian tubes:<br>0: not enclosed between basisphenoid and basioccipital<br>1: entirely enclosed	Clark 1994 (52)
247.	Basisphenoid rostrum (cultriform process) shape:<br>0: slender - not dorsoventrally expanded<br>1: dorsoventrally expanded	Clark 1994 (53)
248.	Length of basisphenoid rostrum:<br>0: short<br>1: extremely long anteriorly as in *Rhabdognathus*	Jouve 2005 (2)
249.	Basisphenoid rostrum sutured dorsally with laterosphenoid<br>0: absent – basisphenoid rostrum separate from laterosphenoid<br>1: present as in *Dyrosaurus phosphaticus*	Wilberg 2015a (342)
250.	Relative length of basisphenoid and basioccipital in ventral view:<br>0: basisphenoid shorter or equal to basioccipital<br>1: basisphenoid longer and transversely wider than basioccipital as in *Protosuchus*	Ortega et al. 2000 (68)
251.	Basisphenoid exposure in ventral view:<br>0: broadly exposed<br>1: narrowly exposed – nearly excluded from ventral view by pterygoid and basioccipital	Ortega et al. 2000 (67)
252.	Anteroposterior crest on basisphenoid:<br>0: absent – smooth<br>1: present – bears a single median crest<br>2: present – bears two crests<br>3: present – bears three crests	Jouve 2004 (139)
253.	Basisphenoid-pterygoid suture orientation:<br>0: transverse – nearly straight<br>1: basisphenoid tapers anteriorly between the pterygoids	Jouve 2009 (285)
254.	Shape of basisphenoid exposure in ventral view:<br>0: wider than long<br>1: longer than wide	Jouve 2009 (325)
255.	Basisphenoid exposure ventral to basioccipital in occipital view:	Jouve 2009 (331)

	0: absent or slightly exposed<br>1: widely exposed as in *Lomasuchus palpebrosus*	
256.	Anterior extent of basisphenoid in ventral view relative to level of trigeminal foramen (ORDRED):<br>0: basisphenoid terminates near or posterior to anteroposterior level of the trigeminal foramen as in *Pelagosaurus*<br>1: slightly exceeds trigeminal foramen as in *Peipehsuchus teleorhinus*<br>2: extends much further anteriorly, greatly elongated as in *Steneosaurus durobrivensis*	Young et al. 2012 (115)
257.	Basipterygoid process of the basisphenoid:<br>0: present – prominent, forming potentially movable joint with pterygoid<br>1: absent, with basisphenoid joint closed suturally	Clark 1994 (54)
258.	Basipterygoid processes (*inapplicable in taxa lacking basipterygoid processes*)<br>0: simple, without large cavity<br>1: greatly expanded, with large cavity	Clark et al. 2004 (26)
259.	Basisphenoid-exoccipital suture:<br>0: absent<br>1: interdigitated suture lateral to the lateral eustachian foramina	Pol et al. 2013 (96)
260.	Basioccipital, midline crest on basioccipital plate below occipital condyle:<br>0: absent<br>1: present	Turner and Sertich, 2010 (297)
261.	Prootic exposure on lateral surface of braincase:<br>0: widely exposed<br>1: very little exposure – obscured by expansion of quadrate and laterosphenoid	Wilberg 2015a (343)
262.	At maturity, prootic/laterosphenoid suture raised, forming a dorsoventrally directed crest (directly above foramen for cranial nerve V) separating fossae for muscle attachment (M. pseudotemporalis profundus and M. adductor mandibulae externus profundus; Holliday and Witmer, 2009; ORDERED) *this crest is greatly reduced or absent in small juveniles of taxa possessing the crest in larger specimens*:<br>0: absent	Wilberg 2015a (357)

	1: slightly raised as in *Metriorhynchus superciliosus*<br>2: present as a strong crest as in *Pelagosaurus typus*	
263.	Exit for trigeminal and middle cerebral vein in prootic:<br>0: single circular or ovate opening<br>1: bilobate (or hour-glass shaped) with anterior projection of prootic slightly dividing into dorsal (for middle cerebral vein) and ventral (for exit of trigeminal branches) portions as in *Pelagosaurus typus, Metriorhynchus westermani*,<br>2: fully divided into two openings by a bridge formed by prootic, dorsal opening interpreted as middle cerebral vein and ventral opening as exit for trigeminal as in *Steneosaurus bollensis*	Nesbitt 2011 (131)
264.	Otic capsule size (ORDERED):<br>0: not enlarged, protruding slightly into endocranial cavity as in *Crocodylus niloticus*<br>1: inflated but not meeting at midline within endocranial cavity as in *Chenanisuchus lateroculi*<br>2: highly inflated, meeting at midline within endocranial cavity, dividing cavity into dorsal and ventral chambers as in *Rhabdognathus keinensis*	Wilberg 2015b (261)
265.	Quadratojugal participation in craniomandibular joint:<br>0: absent<br>1: present	Ortega et al. 2000 (99)

Mandible

266.	External mandibular fenestra size (ORDERED):<br>0: absent<br>1: present – reduced to a thin slot as in *Dyrosaurus mahgribensis*<br>2: present – relatively large	Jouve 2004 (148)
267.	Dentary extending posteriorly beneath mandibular fenestra (*inapplicable in taxa lacking an external mandibular fenestra*):<br>0: present<br>1: absent	Clark 1994 (70)
268.	Dorsal edge of dentary:<br>0: straight<br>1: dorsally expanded at caniniform - showing a single concave arch posteriorly<br>2: edge sinusoidal, with two concave waves	Ortega et al. 1996 (1)
269.	Large occlusion pit on dentary lateral to seventh alveolus:<br>0: absent<br>1: present as in *Mahajangasuchus*	Buckley and Brochu 1999 (105)

270.	Groove along lateral margin of dentary ventral to toothrow separating ornamented region (ventrally) from unornamented region (dorsally):<br>0: absent<br>1: present as in *Terminonaris robusta*	Wilberg 2015a (346)
271.	Splenial contribution to symphysis in ventral view (ORDERED):<br>0: not involved in symphysis<br>1: slightly involved in symphysis<br>2: extensively involved in symphysis	Clark 1994 (77)
272.	Mandibular symphysis length:<br>0: short<br>1: long, dentary symphysis prolongs caudal to fourth alveolus	Ortega et al. 2000 (151)
273.	Shape of dentary symphysis in ventral view:<br>0: tapering anteriorly forming an angle<br>1: U-shaped, curving anteriorly<br>2: very broad with extensive transversely oriented anterior edge as in *Simosuchus*	Pol 1999 (212)
274.	Dorsal surface of mandibular symphysis:<br>0: flat or slightly concave<br>1: strongly concave and narrow, trough-shaped as in *Araripesuchus gomesii*	Pol and Apesteguía 2005 (184)
275.	Posteriorly directed peg at symphysis:<br>0: absent<br>1: present as in *Notosuchus terrestris*	Pol and Apesteguía 2005 (181)
276.	Ventral exposure of splenials:<br>0: absent<br>1: present	Ortega et al. 1996 (9)
277.	Thickness of splenial posterior to symphysis:<br>0: thin<br>1: robust dorsally	Ortega et al. 1996 (7)
278.	Orientation of dorsal edge of surangular anterior to glenoid fossa (not the same as coronoid process of surangular):<br>0: flat or concave<br>1: arched dorsally as in *Simosuchus clarki*	Clark 1994 (74)
279.	Longitudinal ridge along the dorsolateral surface of surangular:<br>0: absent<br>1: present as in *Zaraasuchus shepardi*	Pol and Norell 2004b (187)
280.	Shape of surangular-dentary suture in lateral view:<br>0: simple, with little or no interdigitation<br>1: complex, with interlocking prongs from both surangular and dentary (three posterior prongs from dentary and two from surangular) as in *Lomasuchus*	Turner and Buckley 2008 (289)

281.	Surangulodentary groove on lateral surface of surangular and dentary (surangular groove of Gasparini et al., 2006):<br>0: very poorly developed or absent<br>1: present as shallow but conspicuous groove<br>2: well developed, deeply excavated	Young et al. 2012 (135)
282.	Enlarged foramen at anterior end of surangulodentary groove (*inapplicable in taxa lacking distinct surangulodentary groove*):<br>0: absent<br>1: present	Gasparini et al. 2006 (245)
283.	Surangulodentary groove, extent (*inapplicable in taxa lacking distinct surangulodentary groove*):<br>0: groove is longer on the dentary than on the surangular<br>1: groove is as long on the dentary as on the surangular	Young et al. 2012 (136)
284.	Large foramen on lateral surface of surangular:<br>0: absent<br>1: present as in *Junggarsuchus sloani*	Clark et al. 2004 (43)
285.	Distinct coronoid process on surangular:<br>0: absent<br>1: present	Young et al. 2012 (142)
286.	Surangular in dorsal view:<br>0: does not extend beyond the orbit along the dorsal surface of the mandible<br>1: exceeds orbit	Young and Andrade 2009 (47)
287.	Surangular contribution to glenoid fossa:<br>0: forms lateral wall only<br>1: surangular contributes approximately one third or more as in *Dyrosaurus mahgribensis*	Buckley and Brochu 1999 (102)
288.	Orientation of posteriormost part of angular in lateral view:<br>0: laterally oriented: visible in lateral view<br>1: ventrally orientated, not visible laterally but ventrally	Wu et al. 2001 (110)
289.	Insertion area for M. pterygoideous posterior:<br>0: does not extend onto lateral surface of angular<br>1: extends onto lateral surface of angular	Clark 1994 (76)
290.	Sharp ridge on the surface of the angular:<br>0: absent<br>1: present on the ventralmost margin as in *Zaraasuchus shepardi*<br>2: present along the lateral surface as in *Shamosuchus djadochtaensis*	Pol and Norell 2004b (186)
291.	Coronoid length:<br>0: short	Ortega et al. 2000 (98)

	1: elongate, projecting further anteriorly than posterior-most dentary alveolus as in *Metriorhynchus superciliosus*<br>2: absent as in *Dyrosaurus mahgribensis*	
292.	Coronoid, participates on the external face of the mandible (*inapplicable in taxa lacking a coronoid*):<br>0: absent<br>1: present on lateral surface of coronoid process<br>2: present anteriorly between coronoid process and tooth row	Young et al. 2012 (146)
293.	Mandible geometry, relative positions of the dentary tooth-row and coronid process, and development of dorsal curvature of the caudal-end of the mandible:<br>0: gentle curvature in the dorsal margin of the mandible, from the coronoid process to the end of the tooth-row<br>1: strong curvature, raising the coronoid process considerably above the tooth-row	Young et al. 2012 (127)
294.	Mandible geometry, relative position of coronoid process with respect to retroarticular process and glenoid fossa (*inapplicable in taxa lacking a well-defined coronoid process*):<br>0: coronoid process approximately level with dorsal limit of retroarticular process and glenoid fossa<br>1: coronoid process ventral to both retroarticular process and glenoid fossa as in *Dakosaurus maximus*<br>2: coronoid process dorsal to both RA process and glenoid fossa as in *Mahajangasuchus*	Young et al. 2016 (154)
295.	Prearticular:<br>0: present<br>1: absent	Clark 1994 (72)
296.	Articular medial process medial to glenoid fossa (ORDERED):<br>0: absent<br>1: present – not articulating with otoccipital and basisphenoid<br>2: Present – articulating with otoccipital and basisphenoid as in *Protosuchus richardsoni*	Clark 1994 (73)
297.	Size of glenoid fossa of articular relative to articular surface of quadrate:<br>0: anteroposteriorly similar in length<br>1: slightly longer<br>2: much longer – close to 300% of the length of the articular surface of the quadrate	Wu and Sues 1996 (23)
298.	Posterior ridge on glenoid fossa of articular:<br>0: present	Pol and Apesteguía

	1: absent as in *Simosuchus clarki*	2005 (183)
299.	Retroarticular process shape:<br>0: extremely reduced or absent as in *Gracilisuchus*<br>1: very short, broad, and robust as in *Protosuchus richardsoni*<br>2: short, high, and quadrangular - with an extensive rounded, wide, and flat (or slightly concave) surface projected posteroventrally and facing dorsomedially as in *Notosuchus terrestris*<br>3: posterodorsally curving and elongate, triangular shaped and facing dorsally as in *Dyrosaurus mahgribensis*<br>4: posteroventrally projecting and paddle-shaped as in *Shamosuchus djadochtaensis*<br>5: posteriorly elongate and straight as in *Kaprosuchus saharicus*	Clark 1994 (71)
300.	Retroarticular process dorsal extent:<br>0: short, does not exceed to the articular glenoid<br>1: slightly exceeds the articular glenoid<br>2: extremely dorsally curved, greatly exceeds the articular glenoid cavity as in *Dyrosaurus mahgribensis*	Ortega et al. 2000 (93)
301.	Posteromedial process of the retroarticular process: (*inapplicable in taxa lacking a retroarticular process*)<br>0: absent<br>1: present and with pronounced posteromedial process as in *Sphenosuchus acutus*	Wu et al. 2001 (116)
302.	Medial articular shelf of retroarticular process: (*inapplicable in taxa lacking a retroarticular process*)<br>0: absent<br>1: present	Ortega et al. 2000 (141)
303.	Medial shelf of retroarticular process orientation: (*inapplicable in taxa lacking a medial articular shelf on the retroarticular process*)<br>0: vertical and facing medially<br>1: facing dorsally	Ortega et al. 2000 (147)
304.	Medial articular shelf of retroarticular process dorsoventral position (ORDERED): (*inapplicable in taxa lacking a medial articular shelf on the retroarticular process)*<br>0: dorsal in position<br>1: displaced ventrally to approximately mid-point of the retroarticular process as in *Terminonaris robusta*<br>2: extremely displaced ventrally to ventral portion of retroarticular process as in *Chenanisuchus lateroculi*	Jouve 2004 (167, 168)

305.	Longitudinal ridge on lateral surface of surangular beginning just posterolateral to glenoid fossa and extending onto retroarticular process (*inapplicable in taxa lacking a retroarticular process*):<br>0: absent<br>1: present as in *Hyposaurus rogersii*	NEW
306.	Posterior extension of surangular on retroarticular process (*inapplicable in taxa lacking a retroarticular process*):<br>0: extends along entire process, terminating at, or near, posterior limit of articular<br>1: terminates anterior to posterior limit of articular as in *Dyrosaurus maghribensis*	NEW
307.	Anterior margin of mandible (dentary), in dorsal view:<br>0: outer margin converging towards tip or parallel<br>1: distinct notched spatulate shape as in *Steneosaurus bollensis*<br>2: broadens anteriorly, but anterior margin straight as in *Sarcosuchus imperator*	Young et al. 2012 (131)
308.	Anterior of mandible (dentary), interalveolar space size:<br>0: anterior interalveolar spaces are variable in size, ranging from being larger than the proceeding and preceding alveolus to being half the size<br>1: all anterior interalveolar spaces are less than half the length of the adjacent alveoli	Young et al. 2012 (131)
309.	Posteroventral edge of mandibular ramus:<br>0: straight or convex<br>1: strongly deflected ventrally as in *Simosuchus clarki*	Wu et al. 2001 (112)

Dentition and alveolar morphologies

310.	Orientation of premaxillary tooth row:<br>0: curves posterolaterally from midline (“arched”)<br>1: angled (with slight curvature) posterolaterally at approximately 120 degree angle as in *Terminonaris robusta*<br>2: transverse as in *Simosuchus clarki*	Sereno et al. 2001 (69)
311.	Number of premaxillary teeth (ORDERED):<br>0: five<br>1: four<br>2: three<br>3: two	Wu and Sues 1996 (27)
312.	Position of first and second premaxillary alveoli:<br>0: separated like adjacent teeth<br>1: nearly confluent	Sereno et al. 2001 (56)

313.	Size of 4th premaxillary alveolus (when 4 premaxillary alveoli present) *inapplicable in taxa with any other number of premaxillary alveoli*:<br>0: similar in size to other premaxillary alveoli<br>1: 4th extremely reduced as in *Cerrejonisuchus*	NEW
314.	Position of last premaxillary tooth relative to first maxillary tooth:<br>0: anterior or slightly anteromedial<br>1: anterolateral as in *Sarcosuchus imperator*	Sereno et al. 2001 (70)
315.	Premaxillary tooth row, dorsoventral position relative to maxillary row:<br>0: level<br>1: ventrally offset as in *Sarcosuchus imperator*	Sereno et al. 2001 (71)
316.	Number of teeth partially supported by both the premaxilla and maxilla:<br>0: none<br>1: one	Turner and Sertich 2010 (296)
317.	Number of maxillary teeth (ORDERED):<br>0: more than twenty<br>1: eight to twenty<br>2: seven<br>3: six<br>4: five<br>5: four or fewer teeth	Wu and Sues 1996 (30)
318.	Compression of maxillary tooth crowns:<br>0: absent or nearly so<br>1: present – obliquely disposed (asymmetric), tear-drop shaped as in *Notosuchus terrestris*<br>2: present – labiolingually compressed - orientated parallel to the longitudinal axis of skull	Pol 1999 (151)
319.	Maxillary teeth waves (pattern of size variation):<br>0: absent, no tooth size variation<br>1: single wave with the largest alveolus placed near middle of maxillary tooth row as in *Araripesuchus patagonicus*<br>2: enlarged maxillary teeth curved in two waves (festooned) as in *Crocodylus niloticus*	Clark 1994 (79)
320.	Position of first enlarged maxillary tooth:<br>0: no prominent tooth	Ortega et al. 2000 (156)

	1: second or third alveoli enlarged<br>2: fourth or fifth alveoli enlarged	
321.	Position of last maxillary tooth relative to anterior edge of suborbital fenestra:<br>0: last maxillary tooth posterior to anterior edge of suborbital fenestra<br>1: last maxillary tooth anterior to anterior edge of suborbital fenestra	Ortega et al. 2000 (18)
322.	Mid to posterior maxillary teeth, crown–root junction:<br>0: unconstricted<br>1: constricted	Buckley et al. 2000 (117)
323.	Cusps of posterior maxillary and dentary teeth:<br>0: not multicusped<br>1: multicusped	Gomani, 1997 (46)
324.	Morphology of cusps on multicusped teeth: (*inapplicable in taxa lacking multicusped teeth*)<br>0: One main cusp with smaller cusps arranged in one row as in *Simosuchus clarki*<br>1: One main cusp with smaller cusps arranged in more than one row, forming lingual cingulum at base of middle and posterior teeth as in *Malawisuchus mwakasyungutiensis*<br>2: multiple small cusps along edges of occlusal surface as in *Edentosuchus*	Gomani 1997 (47)
325.	Posterior teeth with rings of undulating enamel:<br>0: absent<br>1: present as in *Dakosaurus andiniensis*	Gasparini et al. 2006 (250)
326.	Maxillary dental implantation:<br>0: teeth in isolated alveoli<br>1: teeth in a dental groove as in *Simosuchus clarki*	Oretga et al. 2000 (19)
327.	Edge of the maxillary tooth alveoli relative to palatal process of maxilla in lateral view:<br>0: lower or at the same level as the palate<br>1: higher than region between tooth row (palate sits lower than alveoli in lateral view)	Hua and Jouve 2004 (165)
328.	Maxillary teeth crown facets:<br>0: either lacking or indistinct<br>1: with facets in three distinct planes as in *Geosaurus giganteus*	Young et al. 2012 (164)
329.	Size of anterior dentary teeth opposite premaxilla–maxilla contact relative to other dentary teeth:<br>0: no more than twice length<br>1: more than twice the length	Clark 1994 (80)
330.	Size of dentary teeth, posterior to tooth opposite premaxilla–	Clark 1994 (81)

	maxilla contact:<br>0: equal in size<br>1: enlarged opposite smaller teeth on maxillary tooth row	
331.	Third and fourth dentary alveoli:<br>0: third smaller than fourth – alveoli separated<br>1: third and fourth dentary alveoli roughly equal in size – nearly confluent<br>2: third much larger than 4th as in *Kaprosuchus saharicus*	Jouve 2009 (327)
332.	Size and position of seventh dentary tooth relative to adjacent teeth (*inapplicable in taxa possessing fewer than 8 mandibular teeth*) ORDERED:<br>0: similar in size and spacing to adjacent teeth<br>1: shifted towards eighth tooth, creating a diastema between the 6th and 7th alveolus, but not significantly reduced in size as in *Cerrejonisuchus*, *Phosphatosaurus* (accepts enlarged m3 and reduced m4)<br>2: small and set very close to the eighth tooth as in *Dyrosaurus mahgribensis*	Jouve 2004 (153)
333.	Tooth crown serrations:<br>0: present<br>1: absent	Gasparini et al. 1993 (31)
334.	Tooth serrations (sensu Andrade et al. 2010 – *inapplicable in taxa lacking tooth crown serrations*):<br>0: macroziphodont<br>1: microziphodont	Wilberg 2015b (326)
335.	Occlusion, relation between maxillary and dentary series:<br>0: in-line or interlocked<br>1: maxillary dentition overbites dentary dentition	Young et al. 2012 (173)

Axial skeleton

336.	Atlas intercentrum:<br>0: broader than long<br>1: as long as broad	Clark 1994 (89)
337.	Atlas intercentrum (hypocentrum) length relative to odontoid process:<br>0: long – >15% of odontoid process length<br>1: short – subequal to odontoid process length (±5%)	Young et al. 2012 (176)
338.	Anteroposterior development of neural spine in axis:<br>0: well developed – covering the length of the neural arch<br>1: poorly developed – located over the posterior half of the neural arch as in *Notosuchus terrestris*	Pol 1999 (168)

339.	Prezygapophyses of axis:<br>0: not exceeding anterior margin of neural arch<br>1: exceeding the anterior margin of the neural arch	Pol 1999 (169)
340.	Axis neural arch diapophysis:<br>0: absent<br>1: present	Young et al. 2012 (177)
341.	Shape of vertebral centra:<br>0: roughly cylindrical – centrum expanded slightly at articular surfaces<br>1: spool-shaped – middle portion constricted, centrum expanding greatly near articular surfaces	Buscalioni and Sanz 1988 (35)
342.	Cervical vertebrae (ORDERED):<br>0: amphicoelous or amphiplatyan<br>1: incipiently procoelous – "shallow procoelous"<br>2: procoelous	Clark 1994 (92)
343.	Prezygapophyseal process of anterior cervical vertebrae:<br>0: anterodorsally projected and straight or slightly recurved<br>1: or dorsally projected and strongly recurved	Pol et al. 2012 (296)
344.	Neural spine on posterior cervical vertebrae (ORDERED):<br>0: as broad as those on anterior cervical vertebrae<br>1: posterior spines anteroposteriorly narrow, rod-like<br>2: all spines rod-like	Clark 1994 (90)
345.	Hypapophyses on cervicodorsal vertebrae (ORDERED):<br>0: absent<br>1: well-developed hypapophyses on cervical vertebrae only<br>2: present in cervicals and first two dorsals<br>3: present through third dorsal<br>4: present through fourth dorsal	Clark 1994 (91)
346.	Dorsal vertebrae (ORDERED):<br>0: amphicoelous or amphiplatyan<br>1: incipiently procoelous – "shallow procoelous"<br>2: procoelous	Clark 1994 (93)
347.	Thoracic vertebrae, shallow fossa on the anterior margin of the diapopohysis immediately lateral to the parapophysis:<br>0: present<br>1: absent	Young et al. 2012 (186)
348.	Distinct rounded depression on the dorsal surface of neural arches of the anterior to mid dorsal vertebrae, located between the base of the neural spine and the postzygapophyseal process:	Pol et al. 2012 (302)

	0: absent<br>1: present	
349.	Relative position of the transverse process and the postzygapophysis in mid dorsal vertebrae:<br>0: postzygapophysis located dorsally to the transverse process<br>1: postzygapophysis leveled with the transverse process	Pol et al. 2012 (303)
350.	‘‘Insertion’’ of a sacral vertebra between the first and second primordial sacral vertebrae:<br>0: absent - two sacrals present<br>1: present – three sacrals present with third “inserted” between primordial 1st and 2nd sacral	Buscalioni and Sanz, 1998 (44)
351.	Caudal vertebrae<br>0: all amphicoelous or amphiplatyan<br>1: first caudal vertebra biconvex, with other caudal vertebrae procoelous as in *Alligator mississippiensis*<br>2: first caudal vertebra biconvex, with other caudal vertebrae semiprocoelous, amphicoelous, or amphiplatyan<br>3: all caudal vertebrae procoelous (last sacral procoelous as in *Fruitachampsa*)	Clark 1994 (94)
352.	Height of neural arch of caudal vertebrae relative to centrum length:<br>0: less than two times the length of centrum<br>1: more than two times the length of the centrum as in *Dyrosaurus maghribensis*	Jouve 2009 (303)
353.	Vertebral morphology near distal end of tail:<br>0: distal vertebrae isomorphic to poorly heteromorphic, non-hypocercal<br>1: heteromorphic, bent ventrally, defining lower lobe of tail fin	Young et al. 2012 (192)
354.	Axis rib:<br>0: holocephalous (rib elongate, with one articular head)<br>1: dichocephalous (rib triradiate, with two articular heads)	Young et al. 2012 (193)
355.	Axis rib tuberculum:<br>0: wide with broad dorsal tip<br>1: narrow with acute dorsal tip	Young et al. 2012 (194)
356.	Sacral ribs:<br>0: short, robust, and slightly bent lateroventrally<br>1: long, gracile, and strongly bent ventrally as in *Metriorhynchus superciliosus*	Jouve 2009 (302)
357.	Orientation of sacral ribs:<br>0: horizontal<br>1: arched ventrally, at least in the first sacral	Young et al. 2012 (195)
358.	Shape of posterior chevrons (=haemal arches) in anterior view:	Young et al.

	0: either 'V' or 'Y'-shaped, no distinct anterodorsal process<br>1: posterior chevrons have a 'W'-shape when observed in anterior view, formed by a anterodorsal process rising between the 'Y'-shape	2012 (197)

Appendicular skeleton

359.	Anterior and posterior margins of scapula in lateral view:<br>0: approximately symmetrical<br>1: anterior edge more strongly concave than posterior edge<br>2: posterior margin slightly concave and anterior margin straight as in *Susisuchus anatoceps*	Clark 1994 (82)
360.	Scapular blade width relative to scapulocoracoid articular surface:<br>0: scapular blade reduced, narrower than the scapulocoracoid length as in *Metriorhynchus superciliosus*<br>1: scapular blade equal to or broader than scapulocoracoid articulation length, but less than 200% of the length of the scapulocoracoid articulation as in *Alligator mississippiensis*<br>2: scapular blade very broad and greater than 200% the length of the scapulocoracoid articulation as in *Mahajangasuchus insignis*	Buckley and Brochu 1999 (106)
361.	Coracoid length relative to scapula:<br>0: up to two thirds as long as scapula<br>1: scapula and coracoid approximately equal length	Clark 1994 (83)
362.	Post-glenoid process of coracoid (ORDERED):<br>0: short, knob-like as in *Postosuchus*<br>1: elongate tapering postglenoidal process posteromedially as in *Sphenosuchus acutus*<br>2: elongate ventromedial process expanded ventrally as in *Protosuchus richardsoni*	Clark et al. 2004 (29)
363.	Glenoid surface of coracoid:<br>0: extended on a subhorizontal plane as in *Postosuchus*<br>1: extended on a vertical plane as in *Protosuchus richardsoni*<br>2: extended on an oblique plane, and the glenoid lip facing outwards and posteroventrally as in *Alligator mississippiensis*	Ortega et al. 2000 (122)
364.	Humeral shaft:<br>0: straight<br>1: sigmoidal, with a pronounced posterior curvature of shaft on proximal area of humerus	Ortega et al. 2000 (180)
365.	Distal portion of humeral shaft in cross section:<br>0: rounded<br>1: flattened as in *Metriorhynchus superciliosus, Zoneait*	Jouve 2009 (298)
366.	Length of the humerus relative to length of femur (ORDERED):	Jouve 2009

	0: more than two-thirds<br>1: nearly two-thirds<br>2: nearly one-third<br>3: much less than one-third	(328)
367.	Humerus length relative to scapula:<br>0: much longer than scapula<br>1: shorter than or subequal to scapula as in *Metriorhynchus superciliosus*	Jouve 2009 (329)
368.	Deltopectoral crest of humerus:<br>0: present and robust<br>1: very reduced, nearly continuous with proximal articulation surface as in *Cricosaurus suevicus*	Wilberg 2015a (347)
369.	Ulna length relative to humerus:<br>0: subequal<br>1: more than one-quarter shorter	Jouve 2009 (330)
370.	Ulnar shaft in cross section:<br>0: ovate/round as in other long bones<br>1: flattened	Wilberg 2015a (371)
371.	Radius and ulna length relative to width:<br>0: elongate bones (much longer than wide)<br>1: length and width subequal forming plate-like elements as in *Cricosaurus suevicus*	Wilberg 2015a (348)
372.	Radiale length vs. width (considering its proximal width as reference):<br>0: longer than wide as in *Dibothrosuchus elaphros*<br>1: as long as wide as in *Postosuchus*	Ortega et al. 2000 (127)
373.	Radiale elongation (ORDERED):<br>0: not elongated<br>1: elongated<br>2: greatly elongated, being at least 30% the length of the humerus or femur as in *Almadasuchus*	Benton and Clark 1988 (e)
374.	Proximal and distal ends of radiale:<br>0: proximal end expanded symmetrically, similar to distal end<br>1: proximal head wider than distal one, more expanded proximolaterally than proximomedially	Buscalioni and Sans 1988 (54)
375.	Forelimb (humerus + ulna) vs. hindlimb (femur + tibia) length (ORDERED):<br>0: forelimb greatly reduced, much shorter than hindlimb<br>1: forelimb slightly shorter than hindlimb<br>2: forelimb as long as hind limb as in *Dyrosaurus maghribensis*	Young and Andrade 2009 (109)
376.	Illium:<br>0: large, anteroposteriorly longer than dorsoventral height<br>1: small, dorsoventrally higher than anteroposterior length	Jouve 2009 (299)

377.	Length of preacetabular process of ilium relative to postacetabular process process (*inapplicable in taxa lacking a postacetabular process*):<br>0: similar in length or slightly shorter<br>1: much shorter: one-quarter or less	Clark 1994 (84)
378.	Iliac blade:<br>0: with posterior and anterior laminae subequal in height<br>1: with posterior lamina higher than anterior one	Ortega et al. 2000 (77)
379.	Iluim postacetabular process:<br>0: present<br>1: extremely reduced/absent as in *Metriorhynchus superciliosus*	Wilberg 2015b (368)
380.	Extent of ilium posterior process (*inapplicable in taxa lacking a postacetabular process of the ilium*):<br>0: elongate and robust as in *Alligator mississippiensis, Steneosaurus bollensis*<br>1: anteroposteriorly reduced and fan-shaped as in *Steneosaurus leedsi*	Wilberg 2015b (369)
381.	Posterior end of the postacetabular process (*inapplicable in taxa lacking a postacetabular process of the ilium*):<br>0: tapering posteriorly and ending in an acute or rounded tip<br>1: subrectangular-shaped with the posterior end vertically oriented – dorsoventral height being at least 60% of the height at the origin of the postacetabular process as in *Notosuchus terrestris*	Pol et al. 2012 (326)
382.	Orientation of the ventral margin of the postacetabular process (*inapplicable in taxa lacking a postacetabular process of the ilium*):<br>0: posterodorsally directed<br>1: horizontally or slightly posteroventrally deflected as in *Notosuchus terrestris*	Pol et al. 2012 (327)
383.	Development and orientation of the rugose surface for the insertion of the M. iliotibialis that forms the supracetabular crest (ORDERED):<br>0: reduced, barely present<br>1: mediolaterally narrow and facing dorsally or slightly laterodorsally<br>2: mediolaterally broad, forming a wide and markedly rugose attachment surface facing laterodorsally	Pol et al. 2012 (116)

	3: mediolaterally broad and rugose that is highly deflected laterally forming a remarkably deep acetabulum	
384.	Expanded distal end of pubis:<br>0: absent, pubis rod-like<br>1: present	Clark 1994 (85)
385.	Pubis contribution to acetabulum (ORDERED):<br>0: forming anterior half of ventral edge of acetabulum<br>1: contacting ilium but partially excluded from acetabulum by anterior process of ischium<br>2: pubis completely excluded from acetabulum by anterior process of ischium	Clark 1994 (86)
386.	Length from proximal articular facet of femur to distal end of fourth trochanter:<br>0: more than one-third of total femoral length<br>1: one-third or less of total femoral length	Ortega et al. 2000 (161)
387.	Distal end of femur articular facet for fibula:<br>0: large lateral facet<br>1: very small facet	Clark 1994 (87)
388.	Flange for coccygeofemoralis musculature on anterior margin of femur:<br>0: absent – femur linear<br>1: present as in *Mahajangasuchus insignis*	Buckley and Brochu 1999 (102)
389.	Femur, medial distal condyle:<br>0: tapers to a point on the medial portion in distal view<br>1: smoothly rounded in distal view	Nesbitt 2011 (320)
390.	Femur, distal surface between the lateral and medial condyles:<br>0: nearly flat or flat<br>1: groove separating the medial condyle from the lateral condyle	Nesbitt 2011 (321)
391.	Pseudointernal trochanter (sensu Walker, 1970) in the posterolateral proximal femur for insertion of the *M. pubo-ischio-femoralis externus* (PIFE) muscle:<br>0: absent<br>1: present	Pol et al. 2013 (92)
392.	Tibia, length (ORDERED):<br>0: long (>45% of femur length)<br>1: reduced (31-45% of femur length)<br>2: very reduced (< 30% of femur length)	Young et al. 2012 (225)
393.	Calcaneum tuber (ORDERED):<br>0: well developed – with long neck (subequal in length to main body of calcaneum ±5%), distal end wider than main body of calcaneum & projects inwards the body at >80°	Young and Andrade 2009 (74)

	1: poorly developed – short neck (< half length of calcaneum main body), distal end < half the width of calcaneum main body width & projects out straight from calcaneum<br>2: absent/vestigial – no defined tuber, the posterior edge of calcaneum one smooth, gentle curve	
394.	Phalanges of fifth pedal digit:<br>0: present<br>1: absent	Clark 1994 (88)

Dermal armor

395.	Dorsal osteoderms:<br>0: present<br>1: absent	Wilberg 2015b (382)
396.	Shape of dorsal osteoderms in dorsal view (*inapplicable in taxa lacking dorsal osteoderms*):<br>0: rounded, ovate<br>1: rectangular, wider than long<br>2: square<br>3: rectangular, much wider than long (width > 200% length) as in *Sarcosuchus imperator*	Clark 1994 (95)
397.	Anterolateral process on dorsal osteoderms (*inapplicable in taxa lacking dorsal osteoderms*):<br>0: absent – osteoderms with straight anterior edge<br>1: present as a discrete convexity as in *Gavialis gangeticus*<br>2: present as a well developed articular peg	Clark 1994 (96)
398.	Dorsal primary osteoderm arrangement (ORDERED – *inapplicable in taxa lacking dorsal osteoderms*):<br>0: two parallel, longitudinal rows<br>1: four longitudinal rows<br>2: more than four rows	Clark 1994 (97)
399.	Longitudinal keels on dorsal surface of osteoderms (*inapplicable in taxa lacking dorsal osteoderms*):<br>0: present<br>1: absent	Clark 1994 (101)
400.	Continuity of dorsal armour (*inapplicable in taxa lacking dorsal osteoderms*):<br>0: dorsal amour continues from neck to tail<br>1: dorsal armor shows a distinct narrowing or gap at the cervico-thoracic junction	Ortega et al. 2000 (109)
401.	Dorsal paravertebral osteoderm curvature (*inapplicable in taxa lacking dorsal osteoderms*):<br>0: flat or weakly arched	Nesbitt 2011 (404)

	1: with a distinct ventral bend near lateral margin as in *Postosuchus*, *Araripesuchus*	
402.	Dorsal surface of osteoderms ornamented with anterolaterally and anteromedially directed ridges (fleur de lys pattern of Osmolska et al., 1997 – *inapplicable in taxa lacking dorsal osteoderms*):<br>0: absent<br>1: present as in *Gobiosuchus kielanae*	Pol and Norell 2004b (188)
403.	Sacral osteoderms relative to dorsal osteoderms immediately preceding sacrum (*inapplicable in taxa lacking dorsal osteoderms*):<br>0: similar in size or smaller<br>1: larger as in *Platysuchus multiscrobiculatus*	Wilberg 2015a (372)
404.	Cervical region surrounded by lateral and ventral osteoderms sutured to the dorsal elements:<br>0: absent<br>1: present as in *Gobiosuchus*	Pol and Norell 2004b (189)
405.	Tail osteoderms (*inapplicable in taxa lacking dorsal osteoderms*):<br>0: dorsal osteoderms only<br>1: completely surrounded by osteoderms	Clark 1994 (99)
406.	Ventral trunk osteoderms:<br>0: absent<br>1: present	Clark 1994 (100)
407.	Appendicular osteoderms:<br>0: absent<br>1: present	Pol and Norell 2004b (190)

VI) Supplemental References

Andrews, C. W. 1913. A descriptive catalogue of the marine reptiles of the Oxford Clay, Part Two. London: British Museum (Natural History). 206 pp.

Barbosa J.A., A.W.A. Kellner, and M.S.S. Viana. 2008. A new dyrosaurid crocodylomorph and evidences for faunal turnover at the K-P transition in Brazil. Proceedings of the Royal Society B 275:1385–1391.

Benton, M.J. and J.M. Clark. 1988. Archosaur phylogeny and the relationships of the Crocodylia. Pages 295-338 *in* The phylogeny and classification of the tetrapods, Volume 1 (M. J. Benton, ed.). Clarendon Press, Oxford, England.

Brochu, C.A. 1997a. Morphology, fossils, divergence timing, and the phylogenetic relationships of *Gavialis*. Syst. Biol. 46:479–522.

Brochu, C. A. 1997b. A review of '*Leidyosuchus'* (Crocodyliformes, Eusuchia) from the Cretaceous through Eocene of North America. J. Vert. Paleontol. 17:679–697.

Brochu, C.A. 1999. Phylogenetics, taxonomy, and historical biogeography of Alligatoroidea. Soc. Vert. Paleontol. Mem. 6:9–100.

Brochu CA. 2001. Crocodylian snouts in space and time: phylogenetic approaches toward adaptive radiation. *Amererican Zoologist* 41: 564–585.

Brochu C.A., M.L. Bouaré, F. Sissoko, E.M. Roberts, and M.A. O'Leary. 2002. A dyrosaurid crocodyliform braincase from Mali. J. Paleontol. 76:1060–1071.

Buckley, G.A., and C.A. Brochu. 1999. An enigmatic new crocodile from the upper Cretaceous of Madagascar. Spec. Pap. Palaeontol. 60:149–175.

Buckley, G.A., C.A. Brochu, D.W. Krause, and D. Pol. 2000. A pug-nosed crocodyliform from the Late Cretaceous of Madagascar. Nature 405:941–944.

Buffetaut E. 1979. *Sokotosuchus ianwilsoni* and the evolution of the dyrosaurid crocodilians. Nigerian Field Monographs 1: 31–41.

Buffetaut, E. and S. Hutt. 1980. *Vectisuchus leptognathus*, n. g. n. sp., a slender-snouted goniopholid crocodilian from the Wealden of the Isle of Wight. Neues Jahrb. Geol P.-M. 1980:385–390.

Buscalioni, A.D. and J. L. Sanz. 1988. Phylogenetic relationships of the Atoposauridae (Archosauria, Crocodylomorpha). Hist. Biol. 1:233–250.

Buscalioni A.D., and J.L. Sanz. 1990b. *Montsecosuchus depereti* (Crocodylomorpha, Atoposauridae), new denomination for *Alligatorellus depereti* Vidal, 1915 (Early Cretaceous, Spain): redescription and phylogenetic relationships. J. Vert. Paleontol. 10:244–254.

Buscalioni, A.D., F. Ortega, D.B. Weishampel, and C.M. Jianu. 2001. A revision of the crocodyliform *Allodaposuchus precedens* from the Upper Cretaceous of the Hateg Basin, Romania. Its relevance in the phylogeny of Eusuchia. . J. Vert. Paleontol. 21:74–86.

Carvalho, I.S., L.C.B. Ribeiro, and L.S. Avilla. 2004. *Uberabasuchus terrificus sp. nov.*, a new Crocodylomorpha from the Bauru Basin (Upper Cretaceous), Brazil. Gondwana Res. 7:975–1002.

Clark, J.M. 1986. Phylogenetic relationships of the crocodylomorph archosaurs. Unpublished Ph.D. Dissertation. University of Chicago 556 pp.

Clark JM. 1994. Patterns of evolution in Mesozoic Crocodyliformes. In: Fraser NC, Sues H-D, eds. *In the Shadow of the Dinosaurs. Early Mesozoic Tetrapods*. New York: Cambridge University Press, 84–97.

Clark, J.M. 2011. A new shartegosuchid crocodyliform from the Upper Jurassic Morrison Formation of western Colorado. Zool. J. Linn. Soc.-Lond. 163:S152–S172.

Clark J.M., and M.A. Norell. 1992. The Early Cretaceous Crocodylomorph *Hylaeochampsa vectiana* from the Wealden of the Isle of Wight. Am. Mus. Novit. 3032:1–19.

Clark, J.M. and H.–D. Sues. 2002. Two new basal crocodylomorph archosaurs from the Lower Jurassic and the monophyly of the Sphenosuchia. Zool. J. Linn. Soc.-Lond. 136:77–95.

Clark, J.M., H.–D. Sues, and D.S. Berman. 2000 A new specimen of *Hesperosuchus agilis* from the Upper Triassic of New Mexico and the interrelationships of basal crocodylomorph archosaurs. J. Vert. Paleontol. 20:683–704.

Clark, J.M., X. Xu, C.A. Forster, and Y. Wang. 2004. A Middle Jurassic 'sphenosuchian' from China and the origin of the crocodylian skull. Nature 430:1021–1024.

Colbert E.H. and Mook C.C. 1951**.** The ancestral crocodilian *Protosuchus*. Bull. Am. Mus Nat. Hist. 97:143–182.

Denton, R.K., J.L. Dobie, and D.C. Parris. The marine crocodilian *Hyposaurus* in North America In: Calloway JM, Nicholls BL, eds. *Ancient Marine Reptiles*. New York: Academic Press, 375–397.

Erickson, B.R. 1976. Osteology of the early Eusuchian crocodile *Leidyosuchus formidabilis*, sp. nov. Monogr. Sci. Mus. Minn. (Paleontol.) 2:1–61.

Fiorelli, L. and J.O. Calvo. 2008. New remains of *Notosuchus terrestris* Woodward, 1896 (Crocodyliformes: Mesoeucrocodylia) from the late Cretaceous of Neuquén, Argentina. Arch. Mus. Nac. (Rio de J.) 66:83–124.

Fortier, D., D. Perea, and C. Schultz. 2011. Redescription and phylogenetic relationships of *Meridiosaurus vallisparadisi*, a pholidosaurid from the Late Jurassic of Uruguay. Zool. J. Linn. Soc. 163:S257–S272.

Fraas E. 1902. Die Meer-Krocodilier (Thalattosuchia) des oberen Jura unter specieller Berücksichtigung von Dacosaurus und Geosaurus. Paleontogr. 49: 1–72.

Gao Y. 2001. A new species of *Hsisosuchus* (Mesoeucrocodylia) from Dashanpu, Zigong Municipality, Sichuan Province. Vertebrata PalAsiatica 39: 177–184. [translated by W. Downs]

Gasparini Z.B. 1971. Los Notosuchia del Cretácico de América del Sur como un nuevo infraorden de los Mesosuchia (Crocodilia). Ameghiniana 8:83–103.

Gasparini, Z.B. and G. Chong Diaz. 1977. *Metriorhynchus casamiquelai* n. sp. (Crocodilia, Thalattosuchia), a marine crocodile from the Jurassic (Callovian) of Chile, South America. Neues Jahrb. Geol. P.-A. 153:341–360.

Gasparini Z.B., L.M. Chiappe, and M. Fernandez. 1991. A new Senonian Peirosaurid (Crocodylomorpha) from Argentina and synopsis of the South American Cretaceous crocodilians. J. Vert. Paleontol. 11: 316–333.

Gasparini, Z., D. Pol, and L.A. Spaletti. 2006. An unusual marine crocodyliform from the Jurassic-Cretaceous boundary of Patagonia. Science 311:70–73.

Georgi, J.A. and D.W. Krause. 2010. Postcranial axial skeleton of *Simosuchus clarki* (Crocodyliformes: Notosuchia) from the Late Cretaceous of Madagascar. Soc. Vert. Paleontol. Mem. 10:99–121.

Gomani E.M. 1997. A crocodyliform from the early Cretaceous Dinosaur beds, northern Malawi. J. Vert. Paleontol. 17:280–294.

Hastings, A.K., J.I. Bloch, E.A. Cadena, and C.A. Jaramillo. 2010. A new small short-snouted dyrosaurid (Crocodylomorpha, Mesoeucrocodylia) from the Paleocene of northeastern Colombia. J. Ver. Paleontol. 30:139–162.

Hastings, A.K., J.I. Bloch, and C.A. Jaramillo. 2014. A new blunt-snouted dyrosaurid, *Anthracosuchus balrogus* gen. et sp. nov. (Crocodylomorpha, Mesoeucrocodylia), from the Paleocene of Colombia. Hist. Bio. 27:998–1020.

Hill, R.V. 2010. Osteoderms of *Simosuchus clarki* (Crocodyliformes: Notosuchia) from the Late Cretaceous of Madagascar. Soc. Vert. Paleontol. Mem. 10:154-176.

Hua, S. and S. Jouve. 2004. A primitive marine gavioaloid from the Paleocene of Morocco. J. Vert. Paleontol. 24:342–350.

Hua S., P. Vignaud, F. Atrops, and A. Clément. 2000. *Enaliosuchus macrospondylus* Koken, 1883 (Crocodylia, Metriorhynchidae)du Valanginien de Barret-le-Bas (Hautes Alpes, France): un cas unique de remontée des narines externs parmi les crocodiliens. Géobios 33:467–474.

Jouve S. 2004. Etude des Crocodyliformes fini Crétacé-Paléogène du Bassin des Oulad Abdoun (Maroc) et comparaison avec les faunes africaines contemporaines: systématique, phylogénie et paléobiogéographie. Unpublished DPhil Thesis. Paris: Muséum National d'Histoire Naturelle.

Jouve, S. 2005. A new description of the skull of *Dyrosaurus phosphaticus* (Thomas, 1893) (Mesoeucrocodylia: Dyrosauridae) from the Lower Eocene of North Africa. Can. J.Earth Sci. 42:323–337.

Jouve, S. 2007. Taxonomic revision of the dyrosaurid assemblage (Crocodyliformes: Mesoeucrocodylia) from the Paleocene of the Iullemmeden Basin, West Africa. J. Paleontol. 81:163–175.

Jouve, S. 2009. The skull of *Teleosaurus cadomensis* (Crocodylomorpha; Thalattosuchia), and phylogenetic analysis of Thalattosuchia. J. Vert. Paleontol. 29:88–102.

Jouve, S., M. Iarochéne, B. Bouya, and M. Amaghzaz. 2005. A short-snouted dyrosaurid (Crocodyliformes, Mesoeucrocodylia) from the Palaeocene of Morocco. Palaeontol. 48:359–369.

Jouve S., B. Bouya, and M. Amaghzaz. 2008. A long-snouted dyrosaurid (Crocodyliformes, Mesoeucrocodylia) from the Paleocene of Morocco: Phylogenetic and paleobiogeographic implications. Palaeontol. 51:381–294.

Kley, N.J., J.J.W. Sertich, A.H. Turner, D.W. Krause, P.M. O'Connor, and J.A. Georgi. 2010. Craniofacial morphology of *Simosuchus clarki* (Crocodyliformes: Notosuchia) from the Late Cretaceous of Madagascar. Soc. Vert. Paleontol. Mem. 10:13–98.

Lapparent de Broin, F. 2002. *Elosuchus*, a new genus of crocodile from the Lower Cretaceous of the North of Africa. C. R. Palevol. 1:275–285.

Larsson, H.C.E., and B. Gado. 2000. A new Early Cretaceous crocodyliform from Niger. Neues Jahrb. Geol P.-A. 217:131–141.

Lauprasert K., G. Cuny, E. Buffetaut, V. Suteethorn, and K. Thirakhupt. 2007. *Siamosuchus phuphokensis*, a new goniopholidid from the Early Cretaceous (ante-

Aptian) of northeastern Thailand. Bulletin de la Société géologique de France 178: 201–216.

Leucona, A. and J.B. Desojo. 2011. Hind limb osteology of *Gracilisuchus stipanicicorum* (Archosauria: Pseudosuchia). Earth Env. Sci. T. R. So. 102:105–128.

Li J., X.-C. Wu, and X. Li. 1994**.** New material of *Hsisosuchus chungkingensis* from Sichuan, China. Vertebrat. Palasiat*.* 32:107–126.

Martin, J.E., U. Deesri, R. Liard, A. Wattanapituksakul, S. Suteethorn, K. Lauprasert, H. Tong, E. Buffetaut, V. Suteethorn, G. Suan, P. Telouk, and V. Balter. 2015. Strontium isotopes and the long-term residency of thalattosuchians in the freshwater environment. Paleobio. 42:143–56.

Martinelli, A.G. 2003. New cranial remains of the bizarre notosuchid *Comahuesuchus brachybuccalis* (Archosauria, Crocodyliformes) from the Late Cretaceous of Rio Negro Province (Argentina). Ameghiniana 40:559–572.

Mercier J. 1933. Contribution à l'étude des Métriorhynchidés (crocodiliens). Annals. Paléont. 22:99–119.

Mook, C.C. 1933. A new species of *Teleorhinus* from the Benton shales. Am. Mus. Novit. 702:1-11

Montefeltro, F.C., H.C.E. Larsson, and M.C. Langer. 2011. A new baurusuchid (Crocodyliformes, Mesoeucrocodylia) from the Late Cretaceous of Brazil and the phylogeny of Baurusuchidae. PLoS ONE 6: e21916. doi:10.1371/journal.pone.0021916

Mueller-Töwe, I.J. 2006. Anatomy, phylogeny, and palaeoecology of the basal thalattosuchians (Mesoeucrocodylia) from the Liassic of Central Europe. Unpublished Ph.D. Dissertation. Universität Mainz 422 pp.

Nash, D.S. 1975. The morphology and relationships of a crocodilian, *Orthosuchus stormbergi*, from the upper Triassic of Lesotho. Ann. S. Afr. Mus. 67:227–329.

Nesbitt, S.J. 2011. The early evolution of archosaurs: Relationships and the origin of major clades. Bull. Am. Mus. Nat. Hist. 352:1–292.

Norell, M.A. and J.M. Clark. 1990. A reanalysis of *Bernissartia fagesii*, with comments on its phylogenetic position and its bearing on the origin and diagnosis of Eusuchia. Bull. Inst. R. Sci. Nat. Belg. Sci. Terre 60:115–128.

Ortega, F., Z. Gasparini, A.D. Buscalioni, and J.O. Calvo. 2000. A new species of *Araripesuchus* (Crocodylomorpha, Mesoeucrocodylia) from the Lower Cretaceous of Patagonia (Argentina). J. Vert. Paleontol. 20:57–76.

Osmolska, H., S. Hua, and E. Buffetaut. 1997. *Gobiosuchus kielanae* (Protosuchia) from the Late Cretaceous of Mongolia: anatomy and relationships. Acta Palaeontol. Pol. 42:257–289.

Pinheiro, A.E.P., R.J. Bertini, M.B. Andrade, and R.G. Neto. 2008. A new specimen of *Striatosuchus maxhechti* (Baurusuchidae, Crocodyliformes) from the Adamantina Formation (Upper Cretaceous), southeastern Brazil. Rev. Bras. Paleontol. 11:37–50.

Pol, D. 1999. El esqueleto postcraneano de *Notosuchus terrestris* (Archosauria: Crocodyliformes) del Cretácico Superior de la Cuenca Neuquina y su información

filogenética. Tesis de Licenciatura, Facultad de Ciencias Exactas y Naturales, Universidad de Buenos Aires, Argentina, 158 pp.

Pol, D. 2003. New remains of *Sphagesaurus huenei* (Crocodylomorpha: Mesoeucrocodylia) from the Late Cretaceous of Brazil. J. Vert. Paleontol. 23:817–831.

Pol, D. 2005. Postcranial remains of *Notosuchus terrestris* Woodward (Archosauria: Crocodyliformes) from the upper Cretaceous of Patagonia, Argentina. Ameghiniana 42:1–21.

Pol, D. and S. Apesteguía. 2005. New *Araripesuchus* remains from the Early Late Cretaceous (Cenomanian) of Patagonia. Am. Mus. Novit. 3490: 1-38.

Pol, D. and Z. Gasparini. 2009. Skull anatomy of *Dakosaurus andiniensis* (Thalattosuchia: Crocodylomorpha) and the phylogenetic position of Thalattosuchia. J. Syst. Palaeontol. 7:163–197.

Pol, D. and M.A. Norell. 2004a. A new crocodyliform from Zos Canyon, Mongolia. Am. Mus. Novit. 3445:1–36.

Pol, D. and M.A. Norell. 2004b. A new gobiosuchid crocodyliform taxon from the Cretaceous of Mongolia. Am. Mus. Novit. 3458:1–31.

Pol, D., A.H. Turner, and M.A. Norell. 2009. Morphology of the Late Cretaceous crocodylomorph *Shamosuchus djadochtaensis* and a discussion of neosuchian phylogeny as related to the origin of Eusuchia. Bull. Am. Mus. Nat. Hist. 324:1–103.

Pol. D., O.W.M. Rauhut, A. Leucona, J.M. Leardi, X. Xu, and J.M. Clark. 2013. A new fossil from the Jurassic of Patagonia reveals the early basicranial evolution and the origins of Crocodyliformes. Biol. Rev. Camb. Philos. Soc. 4:862–872.

Pritchard, A.C., A.H. Turner, E.R. Allen, and M.A. Norell. 2013. Osteology of a North American goniopholidid (*Eutretauranosuchus delfsi*) and palate evolution in Neosuchia. Am. Mus. Novit. 3783:1–56.

Riff, D. and A.W.A. Kellner. 2011. Baurusuchid crocodyliforms as theropod mimics: clues from the skull and appendicular morphology of *Stratiotosuchus maxhechti* (Upper Cretaceous of Brazil). Zool. J. Linn. Soc.-Lond. 163:S37–S56.

Romer, A.S. 1972. The Chañares (Argentina) Triassic reptile fauna. XIII. An early Ornithosuchid pseudosuchian, *Gracilisuchus stipanicicorum*, gen. et sp. nov. Breviora 389:1–24.

Salisbury S.W., P. Willis, and P.M. Sander. 1999. The crocodilian *Goniopholis simus* from the Lower Cretaceous of North-Western Germany. Spec. Pap. Palaeontol. 60: 121–148.

Salisbury, S.W., E. Frey, D.M. Martill, and M.-C. Buchy. 2003. A new crocodilian from the Lower Cretaceous Crato Formation of northeastern Brazil. Palaeontogr. Abt. A 270:3–47.

Schwarz, D. 2002. A new species of *Goniopholis* from the Upper Jurassic of Portugal. Palaeontology 45, 185–208.

Schwarz, D. and S.W. Salisbury. 2005. A new species of *Theriosuchus* (Atoposauridae, Crocodylomorpha) from the Late Jurassic (Kimmeridgian) of Guimarota, Portugal. Geobios. 38:779–802.

Sereno, P.C., H.C.E. Larsson, C.A. Sidor, and B. Gado. 2001. The giant crocodyliform *Sarcosuchus* from the Cretaceous of Africa. Science 294:1516–1519.

Sereno, P.C., C.A. Sidor, H.C.E. Larsson, and B. Gado. 2003. A new notosuchian from the Early Cretaceous of Niger. J. Vert. Paleontol. 23:477–482.

Sertich, J.J.W. and J.R. Groenke. 2010. Appendicular skeleton of *Simosuchus clarki* (Crocodyliformes: Notosuchia) from the Late Cretaceous of Madagascar. Soc. Vert. Paleontol. Mem. 10:122–153.

Smith, D.K., E.R. Allen, R.K. Sanders, and K.L. Stadtman. 2010. A new specimen of *Eutretauranosuchus* (Crocodyliformes: Goniopholididae) from Dry Mesa, Colorado. J. Vert Paleontol 30: 1466–1477.

Turner, A. H. and G. A. Buckley. 2008. *Mahajangasuchus insignis* (Crocodyliformes: Mesoeucrocodylia) cranial anatomy and new data on the origin of the eusuchian-style palate. J. Vert. Paleontol. 28:382–408.

Turner, A.H. and J.J.W. Sertich. 2010. Phylogenetic history of *Simosuchus clarki* (Crocodyliformes: Notosuchia) from the Late Cretaceous of Madagascar. Soc. Vert. Paleontol. Mem. 10:177–236.

Tykoski R.S., T.B. Rowe, R.A. Ketcham, and M.W. Colbert. 2002. *Calsoyasuchus valliceps*, a new Crocodyliform from the Early Jurassic Kayenta Formation of Arizona. J. Vert. Paleontol. 22:593–611.

Walker A.D. 1990. A revision of *Sphenosuchus acutus* Haughton, a crocodylomorph reptile from the Elliot Formation (Late Triassic or Early Jurassic) of South Africa. Phil. T. R. Soc. B 330:1–120.

Weinbaum, J. C. 2002. Osteology and relationships of *Postosuchus kirkpatricki* (Archosauria: Crurotarsi). Unpublished M.S. thesis, Texas Tech University, Lubbock, Texas, USA. 78 pp.

Weinbaum, J. C. 2011. The skull of *Postosuchus kirkpatricki* (Archosauria: Paracrocodyliformes) from the Upper Triassic of the United States. PaleoBios 30:18–44.

Wilberg, E.W. 2015a. A new metriorhynchoid (Crocodylomorpha, Thalattosuchia) from the Middle Jurassic of Oregon and the evolutionary timing of marine adaptations in thalattosuchian crocodylomorphs. J. Vert. Paleontol. 35:e902846.

Wilberg, E.W. 2015b. What's in an outgroup? The impact of outgroup choice on the phylogenetic position of Thalattosuchia (Crocodylomorpha) and the origin of Crocodyliformes. Syst. Biol. 64: 621–637.

Wilberg EW. 2017. Investigating patterns of crocodyliform cranial disparity through the Mesozoic and Cenozoic. Zool. J.Linn. Soc. 181:189–208.

Wu X-C, and S. Chatterjee. 1993. *Dibothrosuchus elaphros*, a crocodylomorph from the lower Jurassic of China and the phylogeny of the Sphenosuchia. J. Vert. Paleontol. 13**:** 58–89.

Wu X.-C., and H.-D. Sues. 1996. Anatomy and phylogenetic relationships of *Chimerasuchus paradoxus*, an unusual crocodyliform reptile from the lower Cretaceous of Hubei, China J. Vert. Paleontol. 16:688–702.

Wu X.-C., W. Brinkman, and J.C. Lu. 1994**.** A new species of *Shantungosuchus* from the Lower Cretaceous of Inner Mongolia (China), with comments on *S.*

chuhsienensis Young, 1961 and the phylogenetic position of the genus. J. Vert. Paleontol. 14: 210–229.

Wu X.-C., D.B. Brinkman, and A.P. Russell. 1996. *Sunosuchus junggarensis* sp.nov. (Archosauria: Crocodyliformes) from the Upper Jurassic of Xinjiang, People's Republic of China. Can. J. Earth Sci. 33:606–630.

Wu X.-C., H.-D. Sues, and Z.M. Dong. 1997**.** *Sichuanosuchus shuhanensis*, a new ?Early Cretaceous protosuchian (Archosauria: Crocodyliformes) from Sichuan (China), and the monophyly of Protosuchia. J. Vert. Paleontol. 17**:** 89–103.

Wu X.-C., A.P. Russel, and S.L. Cumbaa. 2001a. *Terminonaris* (Archosauria: Crocodyliformes): New material from Saskatchewan, Canada, and comments on its phylogenetic relationships. J. Vert. Paleontol. 21:492–514.

Wu X-C, Cheng Z-W, Russell AP. 2001b. Cranial anatomy of a new crocodyliform (Archosauria: Crocodylomorpha) from the Lower Cretaceous of Song-Liao Plain, northeastern China. Can. J. Earth Sci. 38:1653–1663.

Young, M.T., and M.B. Andrade. 2009. What is *Geosaurus*? Redescription of *Geosaurus giganteus* (Thalattosuchia: Metriorhynchidae) from the Upper Jurassic of Bayern, Germany. Zool.l J. Linn. Soc.-Lon. 157:551–585.

Young, M.T., M.B. Andrade, S. Etches, and B.L. Beatty. 2013. A new mteriorhynchid crocodylomorph from the Lower Kimmeridge Clay Formation (Late Jurassic) of England, with implications for the evolution of dermatocranium ornamentation in Geosaurini. Zool.l J. Linn. Soc.-Lon. 169: 820–848.

Zaher, H., D. Pol, A.B. Carvalho, C. Riccomini, D. Campos, and W. Nava. 2006. Redescription of the cranial morphology of *Mariliasuchus amarali*, and its

phylogenetic affinities (Crocodyliformes, Notosuchia). Am. Mus. Novit. 3512:1–40.
